# Electrochemical Palladium‐Catalyzed Oxidative Carbonylation‐Cyclization of Enallenols

**DOI:** 10.1002/anie.202212131

**Published:** 2022-11-04

**Authors:** Jianwei Zhang, Biswanath Das, Oscar Verho, Jan‐E. Bäckvall

**Affiliations:** ^1^ Department of Organic Chemistry, Arrhenius Laboratory Stockholm University 10691 Stockholm Sweden; ^2^ Department of Medicinal Chemistry Uppsala Biomedical Center, BMC Uppsala University 75236 Uppsala Sweden

**Keywords:** Anodic Oxidation, Electrocatalysis, Enallenol, Palladium, Undivided Cell

## Abstract

Herein, we report an electrochemical oxidative palladium‐catalyzed carbonylation‐carbocyclization of enallenols to afford γ‐lactones and spirolactones, which proceeds with excellent chemoselectivity. Interestingly, electrocatalysis was found to have an accelerating effect on the rate of the tandem process, leading to a more efficient reaction than that under chemical redox conditions.

## Graphical abstract

With electricity as green oxidant, a catalytic amount of benzoquinone (BQ) was used as an electron‐transfer mediator (ETM) in palladium‐based electrocatalysis to provide highly efficient access to γ‐lactones and spirolactone skeletons in an undivided cell. This represents the first successful example of an electrochemical oxidative palladium‐catalyzed reaction of allenes.

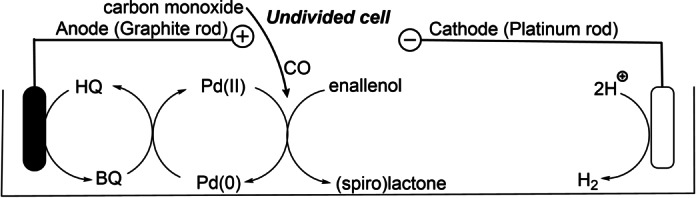

The past decade has witnessed an extensive development of electrochemistry as a powerful tool in organic synthesis.^[1]^ In particular, the combination of transition‐metal‐catalyzed C−H activation and electrochemical oxidation has been successfully used in catalytic oxidative C−H functionalizations. These transition metal‐catalyzed anodic oxidations have unique advantages in regulating the metal valence and regenerating the crucial catalytic species, avoiding the stoichiometric use of expensive or toxic chemical oxidants. Significant contributions have been reported by the groups of Kakiuchi, Mei, Lin, Ackermann, Lei, Xu, and others.[^2^, ^3^] Meanwhile, several investigations on palladium‐catalyzed electrochemical C−H functionalization have been reported.^[4]^ A divided cell is typically required in these electrochemical systems because Pd^II^ species are prone to cathodic reduction, which leads to catalyst deactivation. There are only few successful cases reported on the use of undivided cells in Pd‐catalyzed electrochemical C−H functionalizations (Scheme 1).^[5]^

**Scheme 1:**
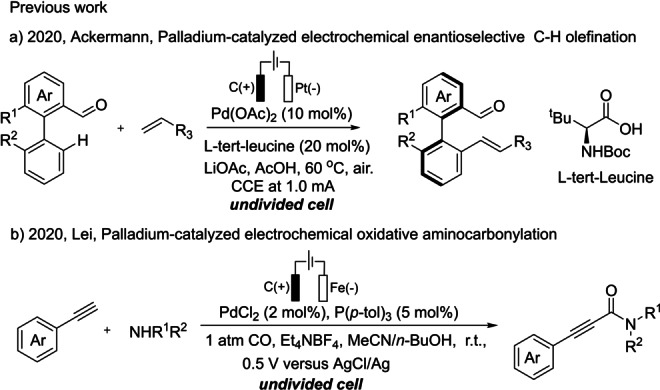
Examples of Pd‐catalyzed electrochemical C−H functionalization.

In recent years, our group has developed a number of palladium‐catalyzed oxidative carbonylation reactions of allenes under atmospheric pressure of carbon monoxide involving a multistep tandem process.^[6]^ These reactions provide an efficient way to construct complex molecules with high atom and step economy. In most cases, benzoquinone (BQ) has been used as stoichiometric oxidant to regenerate the active Pd^II^ species to close the catalytic cycle. Particularly, with the use of electron transfer mediators (ETMs), catalytic amounts of BQ would be enough to realize these transformations under aerobic conditions.^[7]^ As mentioned above, electrochemical reactions, as a green and atom‐economic strategy, have become an effective alternative to the conventional chemical oxidants in the past few years. Based on these state‐of‐the‐art methods, we hypothesized that the reoxidation of Pd^0^ species could be realized by an anodic event, utilizing catalytic amounts of BQ as an ETM, along with cathodic hydrogen evolution (Scheme 2). In an undivided cell, the key to success is that active Pd^II^ species are not reduced at the cathode. Following our growing focus on green and sustainable chemistry, herein we describe the first example of an electrochemical oxidative Pd‐catalyzed carbonylation‐carbocyclization of enallenols.

**Scheme 2:**
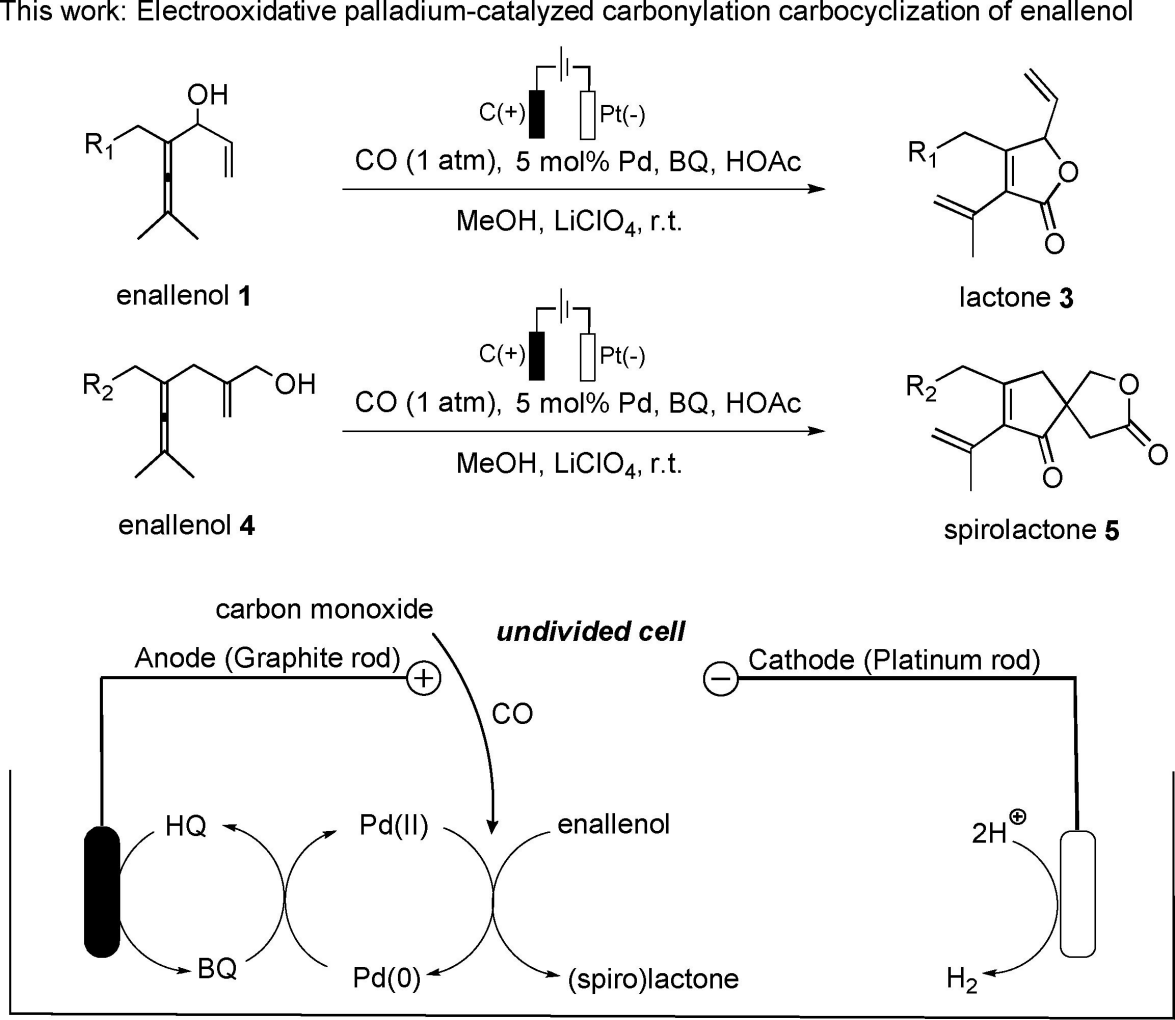
Electrooxidative Pd‐catalyzed C−H functionalization.

Lactones and spirolactones represent important molecular skeletons, which appear in numerous natural alkaloids and bioactive compounds. Thus, it is of high significance to develop new and efficient synthetic methodologies that provide access to these core structures.^[8]^ Our initial attempt of electrochemical oxidative Pd‐catalyzed carbonylation‐carbocyclization began with the reaction of enallenol **1** **a** in the presence of carbon monoxide (CO). We were delighted to find that γ‐lactone **3** **a** was obtained in 37 % yield with Pd(OAc)_2_ as catalyst (entry 1, Table 1).

**Table 1:** Optimization of electrochemical oxidative palladium‐catalyzed carbonylation carbocyclization of enallenols.^[a]^

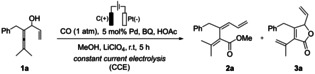				
				
Entry	Pd‐Cat.	Current [mA]	Yield (**2** **a** %)^[b]^	Yield (**3** **a** %)^[b]^
1	Pd(OAc)_2_	4.0	trace	37
2	Pd(TFA)_2_	3.5	6	68
3	Pd(TFA)_2_	4.0	19	61
4	Pd(TFA)_2_	4.5	25	52
5	Pd(TFA)_2_	6.6	33	35
6^[c]^	Pd(TFA)_2_	3.5	8	75
7^[d]^	Pd(TFA)_2_	3.5	20	37
8^[e]^	Pd(TFA)_2_	4.0	trace	83
9^[f]^	Pd(TFA)_2_	4.0	NR	NR
10^[g]^	Pd(TFA)_2_	4.0	NR	NR
11^[h]^	Pd(TFA)_2_	4.0	29	42
12^[i]^	Pd(TFA)_2_	4.0	NR	NR
13^[j]^	Pd(TFA)_2_	0	12	50
14^[k]^	Pd(TFA)_2_	4.0	–	51

[a] Unless otherwise noted the reaction was conducted in methanol solvent (10 mL) at room temperature using **1** **a** (0.2 mmol), BQ (20 mol %) and acetic acid (HOAc, 1.1 μL, 10 mol %) in the presence of Pd source (5 mol %), with LiClO_4_ (2.0 equiv) as electrolyte, which generated a cell voltage of 0.9–1.3 V. [b] isolated yield. [c] HOAc (2.2 μL, 20 mol %) was used as additive. [d] Without HOAc. [e] LiClO_4_ (4.0 equiv) as electrolyte. [f] Acetonitrile (10 mL) as solvent. [g] A mixture solvent of methanol and water (1 : 1, *v*/*v*). [h] N‐Bu_4_NClO_4_ (4.0 equiv) was used as electrolyte. [i] Without BQ, 70 % of starting material recovered. [j] Without power supply, BQ (1.1 equiv) was used as oxidant. [k] 2,6‐dimethylbenzoquinone was used in place of BQ as ETM.

Motivated by this initial result, we set out to optimize the electrochemical reaction conditions for the chemoselective formation of γ‐lactone **3** **a**. A change of Pd(OAc)_2_ to Pd(TFA)_2_ (TFA=trifluoroacetate) improved the yield of **3** **a** to >60 % (entries 2 and 3) An increased current strength resulted in a lower chemoselectivity for the desired γ‐lactone, with more side product being formed from the electrocatalysis (entries 4 and 5). Moreover, acetic acid proved to be an effective additive for the chemoselective formation of γ‐lactone (cf. entries 6 and 7). When 4.0 equivalent of LiClO_4_ was used, the yield of **3** **a** increased to 83 % (entry 8). Further optimization attempts indicated that other reaction media did not improve the electrolysis performance (entries 9, 10). With the tetrabutylammonium perchlorate (*n*‐Bu_4_NClO_4_) in place of LiClO_4_, the electrocatalysis worked less efficiently, providing the γ‐lactone product in only 42 % yield (entry 11), due to the higher cell voltage (1.8 V) generated at the current employed (4 mA), which with LiClO_4_ generated only 0.9–1.3 V. Control experiments confirmed the necessity of benzoquinone (BQ) as redox mediator (entry 12) and without catalytic amounts of BQ, none of the desired γ‐lactone was observed. Without current, using stoichiometric amount of BQ (1.1 equiv), only 50 % yield of **3** **a** was obtained in a less selective reaction (entry 13). The performance of another ETM, 2,6‐dimethylbenzoquinone, was also tested, which afforded the γ‐lactone in 51 % yield (entry 14). Under the optimal condition, the Faradaic yield for the standard reaction is 45 % (the calculation detail seen the Supporting Information).

With these optimized reaction conditions in hand, we investigated the substrate scope of enallenols **1** (Scheme 3). Enallenols with aliphatic and aromatic substituents at the R^1^ position smoothly underwent the carbonylation‐carbocyclization to afford the γ‐lactone products (**3** **a**–**3** **e**) in good yields. Also, when large alkyl substituents were introduced at the R^1^ position, the desired γ‐lactones (**3** **f**, **3** **h** and **3** **i**) were obtained in good yields with excellent chemoselectivity. However, the enallenol with a more bulky *tert*‐butyl substituent (**1** **g**), did not undergo the tandem process to give the corresponding γ‐lactone. In this case, the starting material was recovered in 86 % yield. On the other hand, when an ester group was installed on the allene skeleton, the enallenols (**1** **j** and **1** **k**) were also tolerated under the electrocatalytic conditions and afforded lactones **3** **j** and **3** **k** in 81 % and 73 % yield, respectively. In addition to methyl substituents on the allene moiety, substrates with a cyclopentylidene group worked well to furnish the desired γ‐lactones (**3** **k** and **3** **l**). Moreover, with a terminal methyl substituent at the assisting olefin, γ‐lactone **3** **m** was obtained in only 20 % yield, due to the enhanced steric hindrance for the coordination of the assisting olefin to the metal center. It is noteworthy that the electrolysis of these substrates was complete within 3 h at room temperature, which is a significantly shorter reaction time than that of the corresponding non‐electrochemical reactions with stoichiometric chemical oxidant,[^6g^, ^6j^] This observation indicates the superiority of electrocatalysis over the non‐electrochemical oxidation for this tandem process.

**Scheme 3:**
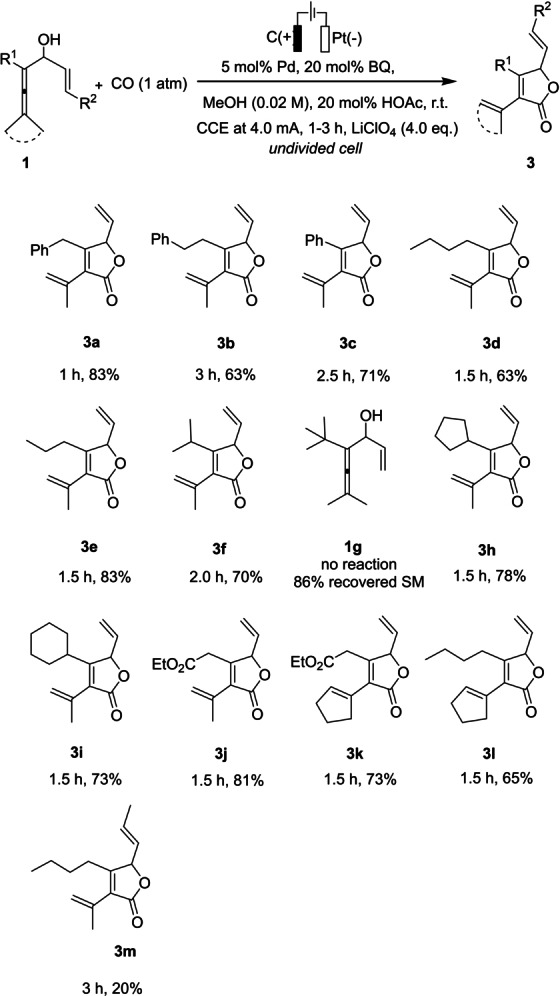
Scope for selective formation of γ‐lactones **3**.

Under the optimized reaction conditions, we further studied the electrochemical formation of spirolactone **5** (Scheme 4). To our delight, the optimal electrochemical condition was also applicable to the chemoselective synthesis of spirolactone **5**. After 3 h of electrolysis of enallenol **4** **a** with methyl substituents on the allene unit, the corresponding spirolactone **5** **a** was obtained as the sole product in 80 % yield, highlighting the excellent chemoselectivity of this reaction under electrochemical conditions. In addition to the dimethyl substituted allene, cyclobutylidene and cyclohexylidene enallenols **4** **b** and **4** **c** provided the desired spirolactone products **5** **b** and **5** **c**, respectively, in good yields. Also, when a benzyl group was installed on the allene moiety (**4** **d**), a high yield was obtained for the desired spirolactone **5** **d**. Notably, 1‐methyl‐1‐phenyl‐substituted enallenol **4** **e** furnished spirocycle **5** **e** in 85 % yield. Enallenol **4** **f** with a free hydroxy group was also found to undergo intramolecular carbocyclization, giving the corresponding spirolactone **5** **f** in moderate yield.

**Scheme 4:**
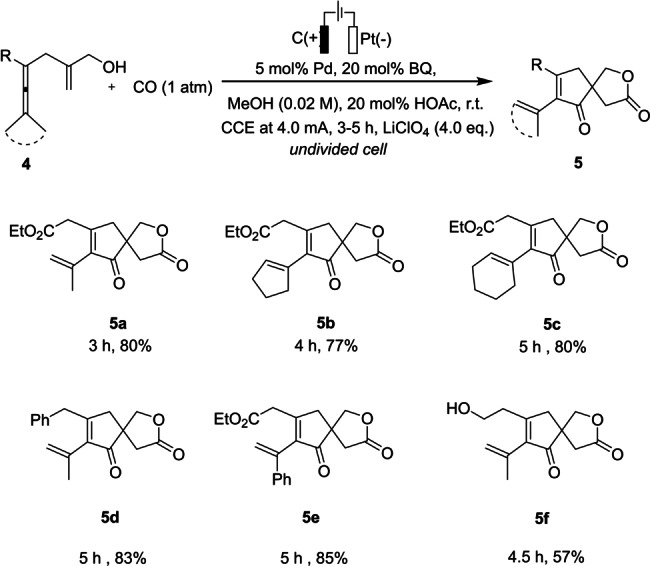
Scope for selective formation of spirolactones **5**.^[9]^

The synthetic utility of the electrochemical palladium‐catalyzed methodology was further demonstrated by the gram‐scale preparation of γ‐lactone **3** **a** (Scheme 5). At a higher concentration (0.04 M) compared to the optimal conditions (0.02 M), with 50 mol % of acetic acid as an additive, the desired γ‐lactone **3** **a** was obtained in 75 % yield. These slightly modified reaction conditions also enabled the gram‐scale electrosynthesis of enallenol **1** **a** (5 mmol) to furnish the target product **3** **a** in 68 % yield.

**Scheme 5:**
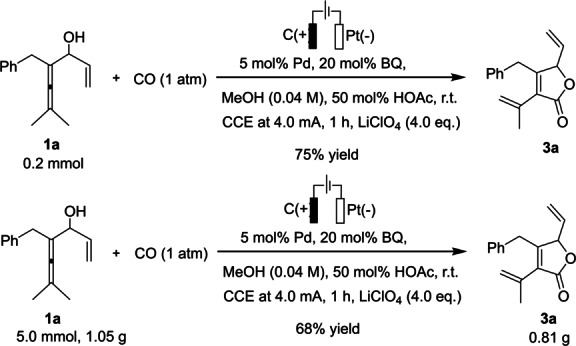
Gram‐scale electrolysis.

On the basis of the experimental results and our previous work on the oxidative Pd^II^‐catalyzed carbonylation‐carbocyclization of allene derivatives,^[6]^ a plausible catalytic cycle is presented for the chemoselective formation of γ‐lactones in Scheme 6. At the start of the reaction, the allene and the assisting olefin simultaneously coordinate to the Pd^II^ center, which promotes allene attack with C−H bond cleavage followed by CO coordination to form Int‐**A**. The subsequent CO insertion into the Pd−C bond of Int‐**A** would afford Int‐**B**. Lactonization of Int‐**B** would provide the γ‐lactone products. Finally, the Pd^0^ is reoxidized to Pd^II^ by BQ generated by the oxidation of hydroquinone (HQ) on the surface of the anode.

**Scheme 6:**
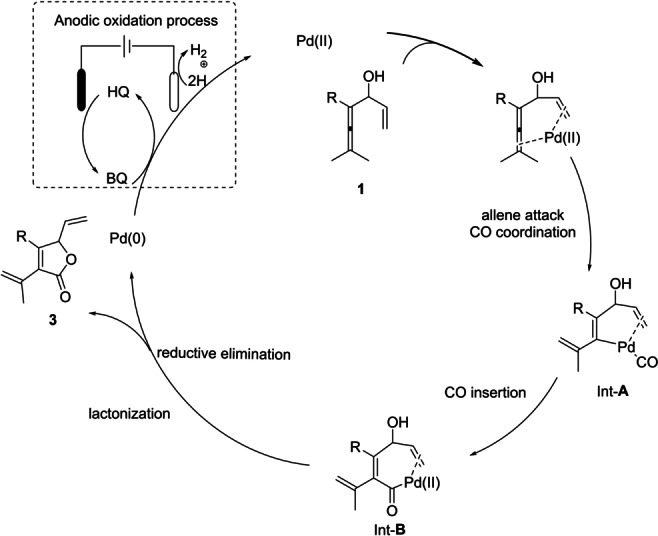
Proposed catalytic cycle.

In conclusion, we have reported on the first electrochemical oxidative Pd‐catalyzed carbonylation‐carbocyclization of enallenols **1** and **4** to afford γ‐lactones **3** and spirolactones **5**, respectively. Both reactions showed a broad substrate scope and excellent tolerance towards different functional groups. As a green and sustainable strategy, avoiding the stoichiometric use of chemical oxidant, the electrocatalytic process has an accelerating effect on this tandem process. Notably, the examples of oxidative electrochemical palladium‐catalyzed cyclizations in undivided cells such as the one described herein are scarce, and most often a divided cell is required for a successful outcome. Studies on further applications of this electrocatalytic system are currently on‐going in our laboratory.

## Conflict of interest

The authors declare no conflict of interest.

## Supporting information

As a service to our authors and readers, this journal provides supporting information supplied by the authors. Such materials are peer reviewed and may be re‐organized for online delivery, but are not copy‐edited or typeset. Technical support issues arising from supporting information (other than missing files) should be addressed to the authors.

Supporting Information

## Data Availability

Research data are not shared.
