# Supplementary material for: Electrochemical Palladium‐Catalyzed Oxidative Carbonylation‐Cyclization of Enallenols
Source: Angew Chem Int Ed Engl. 2022 Nov 4;61(49):e202212131. doi: 10.1002/anie.202212131 (PMC10098644; doi:10.1002/anie.202212131)
Supplement: Supplementary file 1 — Supporting Information [file ANIE-61-0-s001.pdf]

Angewandte Chemie
Eine Zeitschrift der Gesellschaft Deutscher Chemiker

Supporting Information

Electrochemical Palladium-Catalyzed Oxidative Carbonylation-Cyclization of Enallenols

*J. Zhang, B. Das, O. Verho, J.-E. Bäckvall**

Supporting information

Table of contents

General information	S2
Typical procedure for the preparation of starting materials	S3
The optimization of electrochemical reaction condition using CO gas	S11
Typical procedure for the electro-oxidative synthesis of **3** using CO gas	S13
Typical procedure for the electro-oxidative synthesis of **5** using CO gas	S18
References	S20
^{1}H-NMR and ^{13}C-NMR spectrums for compounds	S21

General information

Unless otherwise noted, all reagents were used as received from commercial suppliers. $Pd(OAc)_2$ was obtained from Pressure Chemicals and used without further purification. $Pd(TFA)_2$ was purchased from Sigma-Aldrich. THF and diethyl ether were obtained from a VAC Solvent Purifier. The Graphite electrode and the platinum plated electrode were obtained from IKA. Electrochemical experiments were performed using a AXIOMET AX-3003P potentiostat which can provide a constant input current during standard experiments. Reactions were monitored using thin-layer chromatography (SiO_2). TLC plates were visualized with UV light (254 nm) or $KMnO_4$ stain. Flash chromatography was carried out with 60Å (particle size 35-70 μm) normal flash silica gel. NMR spectra were recorded at 400 MHz (^{1}H) and at 101 MHz (^{13}C), respectively. Chemical shifts (δ) are reported in ppm, using the residual solvent peak in $CDCl_3$ (H = 7.26 and C = 77.0 ppm) as internal standard, and coupling constants (*J*) are given in Hz.

Typical procedure for the preparation of starting materials

Enallenols 1a~1i and 1l~1m were prepared according to reported literature.[1]

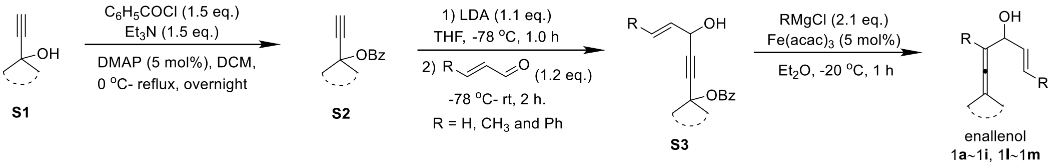

To a solution of **S1** (5.0 mmol), Et_3N (1.47 mL, 7.5 mmol) and 4-dimethylaminopyridine (DMAP, 31.3 mg, 0.25 mmol) in DCM (20 mL) was added benzoyl chloride (1.06 g, 7.5 mmol) dropwise at 0 °C. The mixture was refluxed at 50 °C for 12 h, then cooled to room temperature and carefully quenched with NH_4Cl (sat. aq., 20 mL). The organic layer was separated, and the aqueous layer was extracted with DCM (2 × 30 mL). The combined organic layers were dried over Na_2SO_4, filtered, and concentrated in vacuo. The residue was purified via column chromatography on silica gel (eluent: petroleum ether/diethyl ether = 20/1) to afford **S2**, which was used as the starting material in the next step.

To a solution of **S2** (4.0 mmol) in dry THF (20 mL) was added lithium diisopropylamide (LDA, 4.4 mL, 4.4 mmol, 1.0 M/THF) dropwise at -78 °C under Ar atmosphere. The mixture was stirred for 10 min at this temperature. After that, the appropriate aldehyde (4.8 mmol) was added, and the reaction mixture was allowed to stir at room temperature for 2 h. After the reaction was completed, the mixture was carefully quenched with H_2O (10 mL), and diluted with Et_2O (20 mL). The organic layer was separated, and the aqueous layer was extracted with Et_2O (2 × 30 mL). The combined organic layers were dried over Na_2SO_4, filtered, and concentrated in vacuo. The residue was purified via column chromatography on silica gel (eluent: petroleum ether/diethyl ether = 2/1) to afford **S3**, which was used as the starting material in the next step.

Compound **S3** (3.6 mmol) and $Fe(acac)_3$ (63.6 mg, 0.18 mmol) in dry Et_2O (20 mL) under Ar atmosphere was stirred at -20 °C for 10 min, and then RMgCl (3.8 mL, 7.6 mmol, 2.0 M/Et_2O) was added dropwise. The reaction mixture was stirred for another 40 min at this temperature, and then slowly quenched with HCl (aq., 1.0 M, 15 mL), extracted with Et_2O (2 × 30 mL) and the combined organic layers were dried over Na_2SO_4, filtered, and

concentrated in vacuo. The residue was purified via column chromatography on silica gel (eluent: petroleum ether/diethyl ether = 8/1) to afford enallenol **1**.

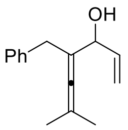

1a

4-Benzyl-6-methylhepta-1,4,5-trien-3-ol (**1a)**. Colorless oil. ^{1}H NMR (400 MHz, $CDCl_3$) δ 7.31 – 7.25 (m, 2H), 7.23 – 7.14 (m, 3H), 5.83 (ddd, *J* = 17.0, 10.2, 6.7 Hz, 1H), 5.31 – 5.21 (m, 1H), 5.21 – 5.12 (m, 1H), 4.45 – 4.35 (m, 1H), 3.37 (d, *J* = 15.0 Hz, 1H), 3.24 (d, *J* = 15.1 Hz, 1H), 1.79 (d, *J* = 5.5 Hz, 1H), 1.66 (d, *J* = 5.6 Hz, 6H). ^{13}C NMR (101 MHz, $CDCl_3$) δ 197.9, 139.9, 139.5, 129.1, 128.3, 126.2, 115.7, 105.7, 101.5, 72.8, 36.5, 20.8, 20.8.

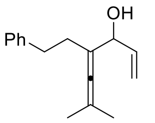

1b

6-Methyl-4-phenethylhepta-1,4,5-trien-3-ol (**1b)**. Colorless oil. ^{1}H NMR (400 MHz, $CDCl_3$) δ 7.31 – 7.25 (m, 2H), 7.22 – 7.14 (m, 3H), 5.80 (ddd, *J* = 17.0, 10.2, 6.6 Hz, 1H), 5.26 (dt, *J* = 17.1, 1.4 Hz, 1H), 5.14 (dt, *J* = 10.3, 1.3 Hz, 1H), 4.40 (dd, *J* = 6.7, 5.3 Hz, 1H), 2.73 (t, *J* = 7.8 Hz, 2H), 2.36 – 2.18 (m, 2H), 1.81 (d, *J* = 5.4 Hz, 1H), 1.68 (d, *J* = 1.3 Hz, 6H). ^{13}C NMR (101 MHz, $CDCl_3$) δ 196.5, 142.2, 139.6, 128.5, 128.4, 125.9, 115.6, 105.7, 102.3, 73.6, 34.1, 30.6, 21.0, 21.0.

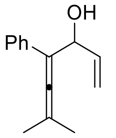

1c

6-Methyl-4-phenylhepta-1,4,5-trien-3-ol (**1c)**. Colorless oil. ^{1}H NMR (400 MHz, $CDCl_3$) δ 7.46 – 7.38 (m, 2H), 7.32 (dd, *J* = 8.5, 6.9 Hz, 2H), 7.25 – 7.17 (m, 1H), 6.01 (ddd, *J* = 17.1, 10.3, 5.8 Hz, 1H), 5.36 (dt, *J* = 17.1, 1.4 Hz, 1H), 5.17 (dt, *J* = 10.4, 1.4 Hz, 1H), 5.13 – 5.05 (m, 1H), 1.90 (d, *J* = 6.5 Hz, 1H), 1.85 (d, *J* = 2.4 Hz, 6H). ^{13}C NMR (101 MHz, $CDCl_3$) δ 200.3, 139.5, 136.0, 128.5, 127.0, 126.9, 115.5, 108.0, 102.8, 71.3, 20.6, 20.5.

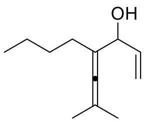

1d

4-(2-Methylprop-1-en-1-ylidene)oct-1-en-3-ol (**1d)**. Colorless oil. ^{1}H NMR (400 MHz, $CDCl_3$) δ 5.79 (dddd, *J* = 17.1, 10.2, 6.7, 0.7 Hz, 1H), 5.31 – 5.19 (m, 1H), 5.13 (dq, *J* = 10.2, 1.1 Hz, 1H), 4.36 (t, *J* = 6.1 Hz, 1H), 1.92 (q, *J* = 7.5 Hz, 2H), 1.84 (d, *J* = 5.4 Hz, 1H), 1.73 (d, *J* = 3.8 Hz, 6H), 1.44 – 1.27 (m, 4H), 0.95 – 0.85 (m, 3H). ^{13}C NMR (101 MHz, $CDCl_3$) δ 196.1, 139.8, 115.4, 106.4, 101.8, 73.5, 30.1, 28.8, 22.5, 21.1, 14.2.

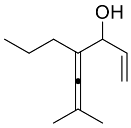

1e

6-Methyl-4-propylhepta-1,4,5-trien-3-ol (**1e)**. Colorless oil. ^{1}H NMR (400 MHz, $CDCl_3$) δ 5.80 (ddd, *J* = 17.0, 10.2, 6.7 Hz, 1H), 5.25 (d, *J* = 17.1 Hz, 1H), 5.13 (d, *J* = 10.3 Hz, 1H), 4.42 – 4.31 (m, 1H), 1.96 – 1.87 (m, 2H), 1.84 (d, *J* = 5.4 Hz, 1H), 1.73 (d, *J* = 3.8 Hz, 6H), 1.48 – 1.36 (m, 2H), 0.91 (t, *J* = 7.3 Hz, 3H). ^{13}C NMR (101 MHz, $CDCl_3$) δ 196.1, 139.8, 115.4, 106.2, 101.8, 73.5, 31.3, 21.1, 21.1, 14.0.

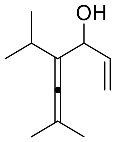

1f

4-Isopropyl-6-methyl-hepta-1,4,5-trien-3-ol (**1f**). Colorless oil. ^{1}H NMR (400 MHz, $CDCl_3$) δ 5.79 (ddd, *J* = 17.1, 10.3, 6.8 Hz, 1H), 5.25 (d, *J* = 17.1 Hz, 1H), 5.12 (d, *J* = 10.2 Hz, 1H), 4.43 (t, *J* = 6.2 Hz, 1H), 2.14 (p, *J* = 6.7 Hz, 1H), 1.81 (d, *J* = 5.7 Hz, 1H), 1.74 (d, *J* = 4.9 Hz, 6H), 1.01 (dd, *J* = 12.0, 6.7 Hz, 6H). ^{13}C NMR (101 MHz, $CDCl_3$) δ 195.3, 140.1, 115.3, 113.6, 103.3, 72.4, 28.6, 22.8, 22.5, 21.1, 21.1.

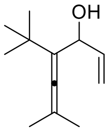

1g

4-(Tert-butyl)-6-methylhepta-1,4,5-trien-3-ol (**1g)**. Colorless oil. ^{1}H NMR (400 MHz, $CDCl_3$) δ 5.86 (ddd, *J* = 16.8, 10.2, 6.5 Hz, 1H), 5.22 (dt, *J* = 17.1, 1.4 Hz, 1H), 5.06 (dt, *J* = 10.2, 1.3 Hz, 1H), 4.59 – 4.48 (m, 1H), 1.72 (d, *J* = 8.9 Hz, 6H), 1.58 – 1.53 (m, 2H), 1.08 (s, 9H). ^{13}C NMR (101 MHz, $CDCl_3$) δ 197.4, 141.5, 116.3, 114.1, 101.5, 70.4, 29.9, 21.0, 20.6.

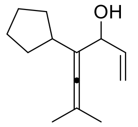

1h

4-Cyclopentyl-6-methylhepta-1,4,5-trien-3-ol (**1h)**. Colorless oil. ^{1}H NMR (400 MHz, $CDCl_3$) δ 5.81 (ddd, *J* = 17.0, 10.2, 6.8 Hz, 1H), 5.26 (dt, *J* = 17.1, 1.4 Hz, 1H), 5.13 (dt, *J* = 10.2, 1.4 Hz, 1H), 4.41 (dd, *J* = 6.8, 3.2 Hz, 1H), 2.31 (p, *J* = 7.7 Hz, 1H), 1.87 – 1.77 (m, 3H), 1.73 (d, *J* = 4.1 Hz, 6H), 1.65 – 1.48 (m, 5H), 1.43 – 1.32 (m, 2H). ^{13}C NMR (101 MHz, $CDCl_3$) δ 195.0, 140.1, 115.3, 111.6, 102.7, 73.1, 39.8, 33.0, 32.6, 25.1, 25.0, 21.1.

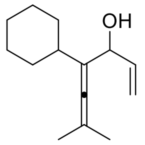

1i

4-Cyclohexyl-6-methyl-hepta-1,4,5-trien-3-ol (**1i)**. Colorless oil. ^{1}H NMR (400 MHz, $CDCl_3$) δ 5.79 (ddd, *J* = 17.0, 10.2, 6.8 Hz, 1H), 5.25 (d, *J* = 17.1 Hz, 1H), 5.11 (d, *J* = 10.2 Hz, 1H), 4.46 – 4.39 (m, 2H), 1.81 (d, *J* = 5.8 Hz, 2H), 1.72 (s, 5H), 1.68 – 1.58 (m, 1H), 1.30 – 1.02 (m, 7H). ^{13}C NMR (101 MHz, $CDCl_3$) δ 196.0, 140.1, 115.2, 112.5, 102.7, 72.3, 38.3, 33.4, 33.0, 26.6, 26.6, 26.4, 21.2.

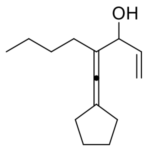

1l

4-(Cyclopentylidene-methylene)oct-1-en-3-ol (**1l)**. Colorless oil. ^{1}H NMR (400 MHz, $CDCl_3$) δ 5.80 (ddd, *J* = 17.0, 10.2, 6.7 Hz, 1H), 5.26 (dt, *J* = 17.1, 1.4 Hz, 1H), 5.13 (dt, *J* = 10.2, 1.3 Hz, 1H), 4.43 – 4.33 (m, 1H), 2.37 (tt, *J* = 6.2, 2.0 Hz, 4H), 1.94 (q, *J* = 7.4 Hz, 2H), 1.89 (d, *J* = 5.4 Hz, 1H), 1.75 – 1.62 (m, 4H), 1.46 – 1.27 (m, 4H), 0.89 (t, *J* = 7.1 Hz, 3H). ^{13}C NMR (101 MHz, $CDCl_3$) δ 191.3, 139.9, 115.4, 110.6, 109.0, 73.7, 31.6, 31.6, 30.0, 29.0, 27.2, 22.5, 14.2.

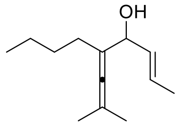

1m

(E)-5-(2-Methyl-prop-1-en-1-ylidene)non-2-en-4-ol (**1m**). Colorless oil. ^{1}H NMR (400 MHz, $CDCl_3$) δ 5.75 – 5.63 (m, 1H), 5.40 (ddq, *J* = 15.2, 7.7, 1.6 Hz, 1H), 4.29 (dd, *J* = 7.6, 4.9 Hz, 1H), 1.96 – 1.82 (m, 3H), 1.77 – 1.67 (m, 9H), 1.43 – 1.27 (m, 4H), 0.89 (t, *J* = 7.1 Hz, 3H). ^{13}C NMR (101 MHz, $CDCl_3$) δ 195.5, 132.9, 127.7, 107.0, 101.9, 73.1, 30.1, 29.0, 22.5, 21.2, 21.1, 17.8, 14.2.

Synthesis of enallenols 1j and 1k

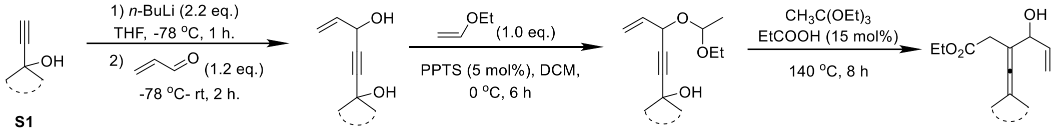

To a solution of **S1** (5.0 mmol) in dry THF (20 mL) was added *n*-BuLi (11.0 mL, 11.0 mmol, 1.0 M/hexane) dropwise at -78 °C under Ar atmosphere. The reaction was stirred for 1 h at this temperature, and then, acrylaldehyde (336 mg, 6.0 mmol) was added dropwise, and the mixture was stirred at room temperature for 2 h. Then the mixture was carefully quenched with H_2O (5 mL), and diluted with Et_2O (10 mL). The organic layer was separated, and the aqueous layer was extracted with Et_2O (2 × 30 mL). The combined organic layers were dried over Na_2SO_4, filtered, and concentrated in vacuo. The residue was purified via column chromatography on silica gel (eluent: petroleum ether/diethyl ether = 2/1) to afford **S4**, which was used as the starting material in the next step.

To a solution of **S4** (4.5 mmol) in DCM (10 mL) was added pyridinium p-toluenesulfonate (PPTS, 58.0 mg, 0.23 mmol) and ethoxyethene (324 mg, 4.5 mmol) at 0 °C. The mixture was stirred at 0 °C for 6 h, and then concentrated in vacuo and purified via column chromatography on silica gel (eluent: petroleum ether/diethyl ether = 4/1) to afford **S5**, which was used as the starting material in the next step.

A dry round-bottomed flask was equipped with a distillation receiver and a condenser. Triethyl orthoacetate (8 mL), propanoic acid (44.4 mg. 0.45 mmol), and **S5** (4.0 mmol) were added sequentially. After the reaction was stirred at 140 °C for 8 h, the mixture was cooled down to 0 °C in an ice bath, and Et_2O (20 mL) and HCl (aq., 1 M, 10 mL) were added slowly. The organic layer was separated quickly, and the aqueous layer was

extracted with Et_2O (2 × 20 mL). The combined organic layers were dried over Na_2SO_4, filtered, evaporated and purified via column chromatography on silica gel (eluent: petroleum ether/diethyl ether = 3/1) to afford the desired enallenol.

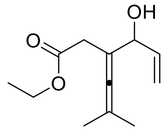

1j

Ethyl 3-(1-hydroxyallyl)-5-methylhexa-3,4-dienoate (**1j**). Colorless oil. ^{1}H NMR (400 MHz, $CDCl_3$) δ 5.83 (ddd, *J* = 17.2, 10.4, 5.8 Hz, 1H), 5.30 (dt, *J* = 17.2, 1.5 Hz, 1H), 5.16 (dt, *J* = 10.3, 1.5 Hz, 1H), 4.60 (ddt, *J* = 6.2, 5.0, 1.4 Hz, 1H), 4.14 (q, *J* = 7.2 Hz, 2H), 3.05 (d, *J* = 15.8 Hz, 1H), 2.97 (d, *J* = 15.9 Hz, 1H), 2.54 (d, *J* = 4.9 Hz, 1H), 1.72 (d, *J* = 1.1 Hz, 6H), 1.26 (t, *J* = 7.1 Hz, 3H). ^{13}C NMR (101 MHz, $CDCl_3$) δ 199.8, 172.7, 138.9, 115.6, 100.0, 98.2, 73.6, 61.0, 35.7, 20.6, 20.6, 14.3.

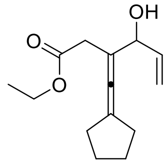

1k

Ethyl 3-(cyclopentylidene-methylene)-4-hydroxyhex-5-enoate (**1k**). Colorless oil. ^{1}H NMR (400 MHz, $CDCl_3$) δ 5.84 (ddd, *J* = 17.3, 10.4, 5.9 Hz, 1H), 5.30 (dt, *J* = 17.2, 1.6 Hz, 1H), 5.15 (dt, *J* = 10.4, 1.5 Hz, 1H), 4.61 (d, *J* = 5.8 Hz, 1H), 4.20 – 4.07 (m, 2H), 3.03 (q, *J* = 15.8 Hz, 2H), 2.38 (ddd, *J* = 7.6, 5.2, 3.0 Hz, 4H), 1.74 – 1.63 (m, 4H), 1.26 (td, *J* = 7.2, 0.6 Hz, 3H). ^{13}C NMR (101 MHz, $CDCl_3$) δ 195.2, 172.8, 139.0, 115.5, 108.7, 100.7, 73.7, 61.0, 35.8, 31.4, 31.3, 27.2, 14.3.

Enallenols 4a~4f were prepared according to a reported literature procedure.[2]

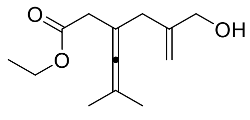

4a

Ethyl 3-(2-(hydroxymethyl)allyl)-5-methyl-hexa-3,4-dienoate (**4a**). Light yellow oil. ^{1}H NMR (400 MHz, $CDCl_3$) δ 5.08 (s, 1H), 4.94 (s, 1H), 4.18 – 4.06 (m, 4H), 2.92 (s, 2H), 2.80 (s, 2H), 1.67 (s, 6H), 1.25 (t, *J* = 7.2 Hz, 3H). ^{13}C NMR (101 MHz, $CDCl_3$) δ 201.4, 172.1, 146.4, 111.9, 96.5, 93.1, 65.3, 60.8, 38.6, 38.1, 20.5, 14.3.

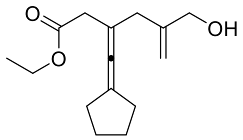

4b

Ethyl 3-(cyclopentylidene-methylene)-5-(hydroxymethyl)hex-5-enoate (**4b)**. Light yellow oil. ^{1}H NMR (400 MHz, $CDCl_3$) δ 5.07 (s, 1H), 4.93 (s, 1H), 4.19 – 4.05 (m, 4H), 2.94 (s, 2H), 2.82 (s, 2H), 2.38 – 2.26 (m, 4H), 1.87 (s, 1H), 1.72 – 1.59 (m, 4H), 1.32 – 1.20 (m, 3H). ^{13}C NMR (101 MHz, $CDCl_3$) δ 196.9, 172.1, 146.4, 111.9, 105.2, 95.7, 65.4, 60.8, 38.8, 38.2, 31.1, 27.1, 14.3.

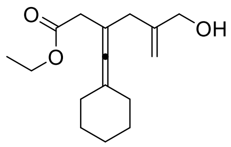

4c

Ethyl 3-(cyclohexylidene-methylene)-5-(hydroxymethyl)hex-5-enoate (**4c)**. Light yellow oil. ^{1}H NMR (400 MHz, $CDCl_3$) δ 5.08 (d, *J* = 1.5 Hz, 1H), 4.93 (d, *J* = 0.9 Hz, 1H), 4.20 – 4.05 (m, 4H), 2.94 (s, 2H), 2.81 (d, *J* = 1.1 Hz, 2H), 2.08 (td, *J* = 6.2, 2.1 Hz, 4H), 1.65 – 1.44 (m, 6H), 1.25 (t, *J* = 7.1 Hz, 3H). ^{13}C NMR (101 MHz, $CDCl_3$) δ 197.8, 172.1, 146.5, 111.9, 104.1, 93.1, 65.4, 60.8, 38.9, 38.3, 31.6, 27.8, 26.2, 14.3.

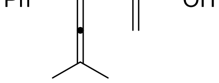

4d

4-Benzyl-6-methyl-2-methylene-hepta-4,5-dien-1-ol (**4d)**. Colorless oil. ^{1}H NMR (400 MHz, $CDCl_3$) δ 7.33 – 7.26 (m, 2H), 7.20 (td, *J* = 6.7, 1.7 Hz, 3H), 5.10 (d, *J* = 1.6 Hz, 1H), 4.93 (d, *J* = 1.4 Hz, 1H), 4.08 (s, 2H), 3.24 (s, 2H), 2.68 (d, *J* = 1.1 Hz, 2H), 1.64 (s, 6H). ^{13}C NMR (101 MHz, $CDCl_3$) δ 201.1, 146.8, 140.2, 129.1, 128.2, 126.1, 111.4, 99.0, 95.5, 65.5, 39.4, 37.3, 20.8.

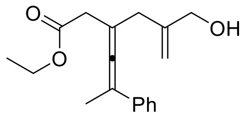

4e

Ethyl 3-(2-(hydroxymethyl)allyl)-5-phenyl-hexa-3,4-dienoate (**4e)**. Light yellow oil. ^{1}H NMR (400 MHz, $CDCl_3$) δ 7.46 – 7.37 (m, 2H), 7.32 (dd, *J* = 8.5, 6.9 Hz, 2H), 7.25 – 7.15 (m, 1H), 5.13 (d, *J* = 1.6 Hz, 1H), 5.00 (d, *J* = 1.3 Hz, 1H), 4.17 – 4.05 (m, 4H), 3.09 (s, 2H), 3.01 – 2.88 (m, 2H), 2.09 (s, 3H), 1.89 – 1.80 (m, 1H), 1.23 (t, *J* = 7.1 Hz, 3H). ^{13}C NMR (101 MHz, $CDCl_3$) δ 204.0, 171.7, 145.9, 137.2, 128.4, 126.9, 126.0, 112.5, 101.8, 97.5, 65.3, 61.0, 38.4, 37.7, 17.1, 14.3.

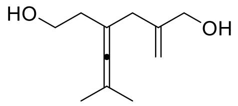

4f

4-(2-Methyl-prop-1-en-1-ylidene)-2-methylenehexane-1,6-diol (**4f)**. Colorless oil. ^{1}H NMR (400 MHz, $CDCl_3$) δ 5.09 (d, *J* = 1.6 Hz, 1H), 4.94 (d, *J* = 1.3 Hz, 1H), 4.09 (s, 2H), 3.69 (d, *J* = 3.7 Hz, 2H), 2.75 (s, 2H), 2.17 (t, *J* = 6.0 Hz, 2H), 1.70 (s, 6H), 1.59 (s, 1H). ^{13}C NMR (101 MHz, $CDCl_3$) δ 199.6, 146.6, 111.7, 96.8, 96.4, 65.5, 61.2, 38.5, 35.3, 20.9.

The optimization of electrochemical reaction condition using CO gas:[a]

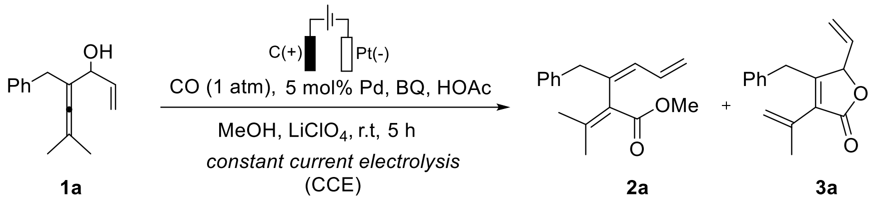

Entry	Pd-Cat.	Current (mA)	Yield(**2a**%)[b]	Yield(**3a**%)[b]
1	$Pd(OAc)_2$	4.0	trace	37
2	$Pd(TFA)_2$	3.5	6	68
3	$Pd(TFA)_2$	4.0	19	61
4	$Pd(TFA)_2$	4.5	25	52
5	$Pd(TFA)_2$	6.6	33	35
6[c]	$Pd(TFA)_2$	3.5	8	75
7[d]	$Pd(TFA)_2$	3.5	20	37
8[e]	$Pd(TFA)_2$	4.0	trace	83
9[f]	$Pd(TFA)_2$	4.0	NR	NR
10[g]	$Pd(TFA)_2$	4.0	NR	NR
11[h]	$Pd(TFA)_2$	4.0	29	42
12[i]	$Pd(TFA)_2$	4.0	NR	NR
13[j]	$Pd(TFA)_2$	0	12	50
14[k]	$Pd(TFA)_2$	4.0	-	51

[a] The reaction was conducted in methanol solvent (10 mL) at room temperature using **1a** (0.2 mmol), BQ (20 mol%) and acetic acid (HOAc, 1.1 µL, 10 mol%) in the presence of Pd source (5 mol%), graphite plate anode, platinum plate cathode, with $LiClO_4$ (2.0 equiv) as electrolyte, which generated cell voltage of 0.9~1.3 V. [b] isolated yield. [c] HOAc (2.2 µL, 20 mol%) was used as additive. [d] without HOAc. [e] $LiClO_4$ (4.0 equiv) as electrolyte. [f] Acetonitrile (10 mL) as solvent. [g] a mixture solvent of methanol and water (1:1, *v/v*). [h] *n*-Bu_4NClO_4 (4.0 equiv) was used as electrolyte. [i] without BQ, 70% of starting material recovered. [j] without power supply, BQ (1.1 equiv) was used as oxidant. [k] 2,6-dimethylbenzoquinone was used in place of BQ as ETM.

The calculation details for the faradaic yield

The quantity of electric charge (Q) provided at 4.0 mA for 5.0 h was calculated:

$Q = I \times t = 4.0\ \text{mA} \times 10^{-3} \times 5.0\ \text{h} \times 3600\ \text{s} = 72$ Coulomb

The quantity of electrons was calculated: (F, faraday constant, 9.65×10^{4} C·mol^{-1})

$n = \frac{Q}{F} = \frac{72\ \text{C}}{9.65 \times 104\ \text{C·mol}-1} = 7.46 \times 10^{-4}$ mol

The quantity of electrons transferred for the desired product was calculated:

$n = 0.2\ \text{mmol} \times 10^{-3} \times 0.83 \times 2 = 3.32 \times 10^{-4}$ mol

The faradaic yield was calculated:

$Yield = \frac{3.32 \times 10^{-4}}{7.46 \times 10^{-4}} = 45\%$

Typical procedure for the electro-oxidative synthesis of 3 using CO gas

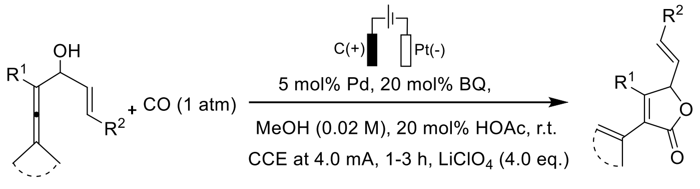

In a 25 mL Schlenk tube was charged $Pd(TFA)_2$ (3.3 mg, 0.01 mmol), BQ (4.3 mg, 0.04 mmol) and $LiClO_4$ (84 mg, 0.8 mmol, 4.0 equiv), 9.0 mL of methanol was added to dissolve the mixture. As shown in Figure S1, the Schlenk tube was sealed with a septum and then purged with CO gas using a balloon for three times under the Schlenk line. A solution of the enallenol (0.2 mmol) and acetic acid (2.2 uL, 0.04 mmol, 0.2 equiv) in 1.0 mL of methanol was injected into the Schlenk tube via a syringe. A two-electrode setup was used having a graphite plate as anode (surface area = $2cm^2$) and a platinum plate as cathode (surface area = $2cm^2$). The constant current electrolysis was performed at 4.0 mA. After the electrolysis, the reaction mixture was washed with 20 mL of water, and extracted with dichloromethane (15 mL×2). The combined organic layers were dried over Na_2SO_4, filtered and evaporated under low vacuum on the rotavapor. The residue was purified via column chromatography on silica gel (*n*-pentane/diethyl ether = 30/1, *v*/*v*) to afford the desired product **3**.

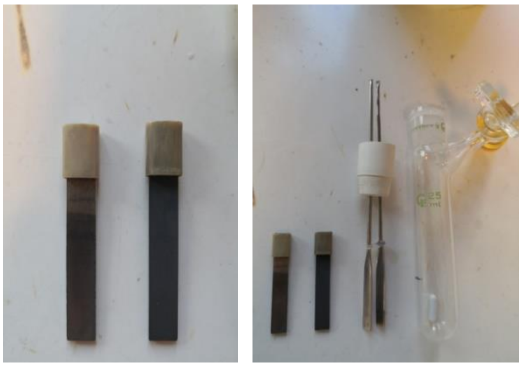

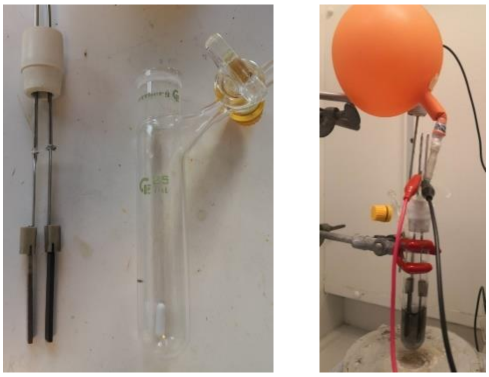

Figure S1. A graphic illustration of the home-made electrolysis set-up

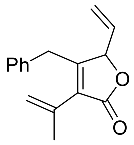

3a

4-Benzyl-3-(prop-1-en-2-yl)-5-vinylfuran-2(5H)-one (**3a**). Colorless oil. Isolated yield 83%. ^{1}H NMR (400 MHz, $CDCl_3$) δ 7.31 (q, *J* = 8.3 Hz, 3H), 7.11 (d, *J* = 6.9 Hz, 2H), 5.61 (ddd, *J* = 16.7, 10.4, 7.6 Hz, 1H), 5.43 – 5.30 (m, 3H), 5.19 (s, 1H), 5.04 (d, *J* = 7.7 Hz, 1H), 4.10 (d, *J* = 15.1 Hz, 1H), 3.47 (d, *J* = 15.2 Hz, 1H), 2.08 (s, 3H). ^{13}C NMR (101 MHz, $CDCl_3$) δ 172.1, 160.6, 136.6, 135.1, 130.1, 132.4, 129.1, 128.8, 127.3, 121.3, 119.1, 82.8, 32.9, 22.0.

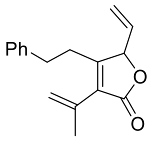

3b

4-Phenethyl-3-(prop-1-en-2-yl)-5-vinylfuran-2(5H)-one (**3b**). Colorless oil. Isolated yield 63%. ^{1}H NMR (400 MHz, $CDCl_3$) δ 7.30 (t, *J* = 7.2 Hz, 2H), 7.23 (t, *J* = 7.3 Hz, 1H), 7.14 (d, *J* = 6.8 Hz, 2H), 5.66 – 5.51 (m, 1H), 5.51 – 5.38 (m, 2H), 5.26 (t, *J* = 1.7 Hz, 1H), 5.10 – 4.99 (m, 2H), 2.99 – 2.87 (m, 1H), 2.87 – 2.78 (m, 2H), 2.55 (dt, *J* = 13.0, 7.4 Hz, 1H), 1.96 (s, 3H). ^{13}C NMR (101 MHz, $CDCl_3$) δ 172.1, 161.5, 140.0, 135.3, 132.6, 129.9, 128.9, 128.4, 126.9, 121.3, 118.7, 83.4, 34.3, 28.7, 21.8.

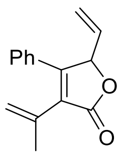

3c

4-Phenyl-3-(prop-1-en-2-yl)-5-vinylfuran-2(5H)-one (**3c**). Colorless oil. Isolated yield 71%. ^{1}H NMR (400 MHz, $CDCl_3$) δ 7.41 (d, *J* = 2.1 Hz, 5H), 5.70 – 5.59 (m, 2H), 5.54 – 5.45 (m, 1H), 5.35 (dt, *J* = 5.1, 1.5 Hz, 2H), 5.33 – 5.26 (m, 1H), 1.85 (s, 3H). ^{13}C NMR (101 MHz, $CDCl_3$) δ 172.0, 158.1, 134.5, 132.5, 131.4, 130.2, 128.8, 128.4, 128.3, 120.8, 120.5, 82.3, 21.8.

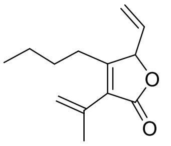

3d

4-Butyl-3-(prop-1-en-2-yl)-5-vinylfuran-2(5H)-one (**3d**). Colorless oil. Isolated yield 63%. ^{1}H NMR (400 MHz, $CDCl_3$) δ 5.62 (ddd, *J* = 17.1, 9.8, 7.4 Hz, 1H), 5.55 – 5.48 (m, 1H), 5.41 (d, *J* = 9.7 Hz, 1H), 5.29 – 5.24 (m, 1H), 5.18 (d, *J* = 7.4 Hz, 1H), 5.07 (s, 1H), 2.58 (ddd, *J* = 13.8, 9.7, 6.5 Hz, 1H), 2.24 (ddd, *J* = 13.9, 9.4, 5.4 Hz, 1H), 2.01 (s, 3H), 1.49 – 1.32 (m, 4H), 0.92 (t, *J* = 7.2 Hz, 3H). ^{13}C NMR (101 MHz, $CDCl_3$) δ 172.3, 163.0, 135.3, 132.7, 129.0, 121.0, 118.6, 83.2, 30.3, 26.7, 22.8, 21.9, 13.8.

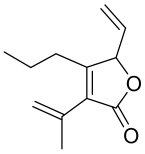

3e

3-(Prop-1-en-2-yl)-4-propyl-5-vinylfuran-2(5H)-one (**3e**). Colorless oil. Isolated yield 83%. ^{1}H NMR (400 MHz, $CDCl_3$) δ 5.62 (ddd, *J* = 17.1, 9.8, 7.4 Hz, 1H), 5.52 (d, *J* = 15.8 Hz, 1H), 5.41 (d, *J* = 9.7 Hz, 1H), 5.26 (t, *J* = 1.7 Hz, 1H), 5.18 (d, *J* = 7.4 Hz, 1H), 5.06 (s, 1H), 2.54 (ddd, *J* = 13.8, 9.6, 6.8 Hz, 1H), 2.24 (ddd, *J* = 14.4, 9.5, 5.5 Hz, 1H), 2.01 (s, 3H), 1.57 – 1.41 (m, 2H), 0.95 (t, *J* = 7.4 Hz, 3H). ^{13}C NMR (101 MHz, $CDCl_3$) δ 172.3, 162.7, 135.3, 132.7, 129.3, 121.0, 118.6, 83.2, 28.9, 22.0, 21.6, 14.2.

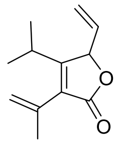

3f

4-Isopropyl-3-(prop-1-en-2-yl)-5-vinylfuran-2(5H)-one (**3f**). Colorless oil. Isolated yield 70%. ^{1}H NMR (400 MHz, $CDCl_3$) δ 5.67 (ddd, *J* = 17.4, 8.9, 8.0 Hz, 1H), 5.55 (d, *J* = 17.0 Hz, 1H), 5.42 (d, *J* = 9.8 Hz, 1H), 5.31 – 5.21 (m, 2H), 4.98 (s, 1H), 3.01 (p, *J* = 7.1 Hz, 1H), 1.99 (s, 3H), 1.23 –

1.14 (m, 6H). ^{13}C NMR (101 MHz, $CDCl_3$) δ 172.4, 167.5, 135.8, 133.5, 129.1, 121.2, 118.6, 82.8, 28.0, 22.3, 22.2, 20.6.

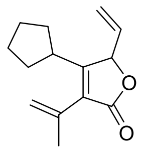

3h

4-Cyclopentyl-3-(prop-1-en-2-yl)-5-vinylfuran-2(5H)-one (**3h**). Colorless oil. Isolated yield 78%. ^{1}H NMR (400 MHz, $CDCl_3$) δ 5.66 (ddd, J = 17.3, 9.7, 7.9 Hz, 1H), 5.59 – 5.51 (m, 1H), 5.46 – 5.38 (m, 1H), 5.30 – 5.24 (m, 1H), 5.21 (d, J = 7.9 Hz, 1H), 4.98 (s, 1H), 2.62 (tt, J = 12.2, 3.3 Hz, 1H), 1.99 (s, 3H), 1.81 – 1.64 (m, 5H), 1.57 – 1.42 (m, 2H), 1.31 – 1.16 (m, 3H). ^{13}C NMR (101 MHz, $CDCl_3$) δ 172.4, 165.5, 135.9, 133.3, 129.7, 121.0, 118.5, 83.1, 39.3, 32.6, 31.7, 25.9, 25.6, 22.3.

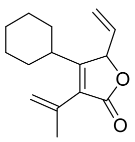

3i

4-Cyclohexyl-3-(prop-1-en-2-yl)-5-vinylfuran-2(5H)-one (**3i**). Colorless oil. Isolated yield 73%. ^{1}H NMR (400 MHz, $CDCl_3$) δ 5.66 (ddd, J = 17.3, 9.7, 7.9 Hz, 1H), 5.59 – 5.51 (m, 1H), 5.46 – 5.38 (m, 1H), 5.30 – 5.24 (m, 1H), 5.21 (d, J = 7.9 Hz, 1H), 4.98 (s, 1H), 2.62 (tt, J = 12.2, 3.3 Hz, 1H), 1.99 (s, 3H), 1.81 – 1.64 (m, 5H), 1.57 – 1.42 (m, 2H), 1.31 – 1.16 (m, 3H). ^{13}C NMR (101 MHz, $CDCl_3$) δ 172.5, 166.7, 135.9, 133.4, 129.2, 121.2, 118.5, 83.1, 38.8, 32.2, 30.9, 26.4, 26.3, 25.9, 22.5.

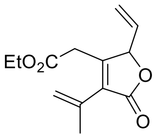

3j

Ethyl 2-(5-oxo-4-(prop-1-en-2-yl)-2-vinyl-2,5-dihydrofuran-3-yl)acetate (**3j**). Colorless oil. Isolated yield 81%. ^{1}H NMR (400 MHz, $CDCl_3$) δ 5.62 (ddd, J = 16.8, 9.3, 7.4 Hz, 1H), 5.57 – 5.49 (m, 1H), 5.47 – 5.39 (m, 2H), 5.30 (t, J = 1.6 Hz, 1H), 5.12 (s, 1H), 4.18 (q, J = 7.1 Hz, 2H), 3.64 – 3.55 (m, 1H), 3.28 (d, J = 16.2 Hz, 1H), 2.03 (s, 3H), 1.28 (t, J = 7.2 Hz, 3H). ^{13}C NMR (101 MHz, $CDCl_3$) δ 171.5, 168.4, 153.8, 135.0, 132.3, 121.7, 119.5, 83.6, 61.8, 32.8, 21.5, 14.3.

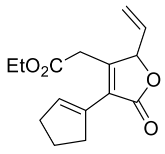

3k

Ethyl 2-(4-(cyclopent-1-en-1-yl)-5-oxo-2-vinyl-2,5-dihydrofuran-3-yl)acetate (**3k**). Colorless oil. Isolated yield 73%. ^{1}H NMR (400 MHz, $CDCl_3$) δ 6.60 (s, 1H), 5.64 – 5.54 (m, 1H), 5.56 – 5.48 (m, 1H), 5.45 – 5.39 (m, 1H), 5.36 (d, *J* = 7.0 Hz, 1H), 4.18 (q, *J* = 7.1 Hz, 2H), 3.67 (d, *J* = 16.2 Hz, 1H), 3.32 (d, *J* = 16.2 Hz, 1H), 2.68 (tq, *J* = 6.3, 2.0 Hz, 2H), 2.47 (dt, *J* = 5.2, 2.7 Hz, 2H), 1.95 (q, *J* = 7.4 Hz, 2H), 1.27 (t, *J* = 7.1 Hz, 3H). ^{13}C NMR (101 MHz, $CDCl_3$) δ 171.9, 168.6, 151.0, 136.2, 132.6, 126.0, 121.8, 83.4, 61.8, 34.2, 33.0, 23.3, 14.3.

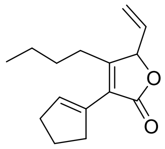

3l

4-Butyl-3-(cyclopent-1-en-1-yl)-5-vinylfuran-2(5H)-one (**3l**). Colorless oil. Isolated yield 65%. ^{1}H NMR (400 MHz, $CDCl_3$) δ 6.52 (t, *J* = 2.4 Hz, 1H), 5.60 (ddd, *J* = 17.1, 9.7, 7.4 Hz, 1H), 5.54 – 5.46 (m, 1H), 5.40 (d, *J* = 9.5 Hz, 1H), 5.14 (d, *J* = 7.4 Hz, 1H), 2.71 – 2.58 (m, 3H), 2.51 – 2.43 (m, 2H), 2.27 (ddd, *J* = 13.8, 9.6, 5.7 Hz, 1H), 1.94 (q, *J* = 7.6 Hz, 2H), 1.51 – 1.43 (m, 2H), 1.40 – 1.33 (m, 2H), 0.92 (t, *J* = 7.2 Hz, 3H). ^{13}C NMR (101 MHz, $CDCl_3$) δ 172.7, 160.8, 134.6, 133.1, 132.9, 123.0, 120.9, 83.1, 34.4, 33.0, 30.9, 27.0, 23.3, 23.0, 13.9.

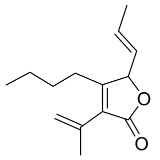

3m

(E)-4-Butyl-5-(prop-1-en-1-yl)-3-(prop-1-en-2-yl)furan-2(5H)-one (**3m**). Colorless oil. Isolated yield 20%. ^{1}H NMR (400 MHz, $CDCl_3$) δ 6.04 – 5.90 (m, 1H), 5.31 – 5.18 (m, 2H), 5.14 (d, *J* = 8.5 Hz, 1H), 5.06 (s, 1H), 2.55 (ddd, *J* = 13.8, 9.6, 6.4 Hz, 1H), 2.22 (ddd, *J* = 14.2, 9.4, 5.5 Hz, 1H), 2.01 (s, 3H), 1.81 – 1.75 (m, 3H), 1.49 – 1.33 (m, 4H), 0.91 (t, *J* = 7.2 Hz, 3H). ^{13}C NMR (101 MHz, $CDCl_3$) δ 172.4, 163.5, 135.5, 133.6, 129.0, 125.8, 118.4, 83.2, 30.3, 26.7, 22.8, 22.0, 18.0, 13.8.

Typical procedure for the electro-oxidative synthesis of 5 using CO gas

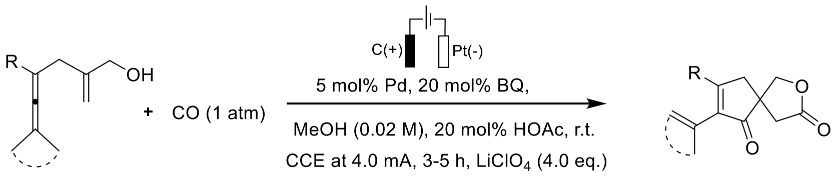

In a 25 mL Schlenk tube was charged Pd(TFA)$_2$ (3.3 mg, 0.01 mmol), BQ (4.3 mg, 0.04 mmol) and LiClO$_4$ (84 mg, 0.8 mmol, 4.0 equiv), 9.0 mL of methanol was added to dissolve the mixture. The Schlenk tube was sealed with a septum and then purged with CO gas using a balloon for three times under the Schlenk line. A solution of the enallenol **4** (0.2 mmol) and acetic acid (2.2 uL, 0.04 mmol, 0.2 equiv) in 1.0 mL of methanol was injected into the Schlenk tube via a syringe. The constant current electrolysis was performed at 4.0 mA. After the electrolysis, the reaction mixture was washed with 20 mL of water, and extracted with dichloromethane (15 mL×2). The combined organic layers were dried over Na$_2$SO$_4$, filtered and evaporated under low vacuum on the rotavapor. The residue was purified via column chromatography on silica gel (*n*-pentane/diethyl ether = 30/1, *v*/*v*) to afford the desired product **5**.

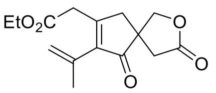

5a

Ethyl 2-(3,9-dioxo-8-(prop-1-en-2-yl)-2-oxaspiro[4.4]non-7-en-7-yl)acetate (**5a**). Colorless oil. Isolated yield 80%. ^{1}H NMR (400 MHz, CDCl$_3$) δ 5.25 (s, 1H), 4.87 (d, *J* = 0.9 Hz, 1H), 4.33 (d, *J* = 8.9 Hz, 1H), 4.20 (t, *J* = 7.1 Hz, 2H), 4.16 – 4.08 (m, 1H), 3.62 – 3.47 (m, 2H), 3.03 – 2.90 (m, 2H), 2.83 (d, *J* = 18.6 Hz, 1H), 2.40 (d, *J* = 17.3 Hz, 1H), 1.92 (s, 3H), 1.28 (t, *J* = 7.1 Hz, 3H). ^{13}C NMR (101 MHz, CDCl$_3$) δ 204.5, 174.9, 168.8, 163.3, 144.0, 136.2, 118.4, 76.4, 61.7, 51.3, 45.3, 39.0, 37.1, 21.8, 14.3.

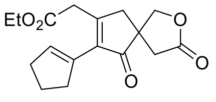

5b

Ethyl 2-(8-(cyclopent-1-en-1-yl)-3,9-dioxo-2-oxaspiro[4.4]non-7-en-7-yl)acetate (**5b**). Colorless oil. Isolated yield 77%. ^{1}H NMR (400 MHz, CDCl$_3$) δ 6.18 (s, 1H), 4.32 (d, *J* = 8.9 Hz, 1H), 4.19

(q, J = 7.2 Hz, 2H), 4.11 (d, J = 8.9 Hz, 1H), 3.67 – 3.52 (m, 2H), 2.97 (dd, J = 17.9, 3.4 Hz, 2H), 2.83 (d, J = 18.7 Hz, 1H), 2.60 (ddd, J = 10.0, 4.7, 2.2 Hz, 2H), 2.46 (dt, J = 7.3, 4.2 Hz, 2H), 2.38 (d, J = 17.2 Hz, 1H), 1.92 (p, J = 7.5 Hz, 2H), 1.28 (t, J = 7.1 Hz, 3H). ^{13}C NMR (101 MHz, $CDCl_3$) δ 204.9, 175.0, 168.8, 162.0, 138.1, 134.7, 133.5, 76.6, 61.8, 51.3, 45.7, 39.2, 37.7, 34.6, 33.1, 23.5, 14.3.

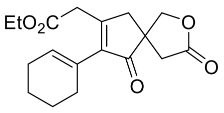

5c

Ethyl 2-(8-(cyclohex-1-en-1-yl)-3,9-dioxo-2-oxaspiro[4.4]non-7-en-7-yl)acetate (**5c**). Colorless oil. Isolated yield 80%. ^{1}H NMR (400 MHz, $CDCl_3$) δ 5.60 (s, 1H), 4.31 (d, J = 8.8 Hz, 1H), 4.19 (q, J = 7.1 Hz, 2H), 4.14 – 4.06 (m, 1H), 3.58 – 3.44 (m, 2H), 2.95 (d, J = 17.2 Hz, 2H), 2.80 (d, J = 18.4 Hz, 1H), 2.38 (d, J = 17.3 Hz, 1H), 2.19 – 2.04 (m, 4H), 1.75 – 1.56 (m, 4H), 1.28 (t, J = 7.1 Hz, 3H). ^{13}C NMR (101 MHz, $CDCl_3$) δ 205.0, 175.1, 169.0, 162.6, 144.5, 130.1, 129.6, 76.5, 61.7, 51.2, 45.2, 39.1, 37.2, 27.4, 25.4, 22.5, 21.9, 14.3.

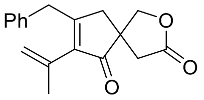

5d

8-Benzyl-7-(prop-1-en-2-yl)-2-oxaspiro[4.4]non-7-ene-3,6-dione (**5d)**. Colorless oil. Isolated yield 83%. ^{1}H NMR (400 MHz, $CDCl_3$) δ 7.40 – 7.27 (m, 3H), 7.18 – 7.07 (m, 2H), 5.33 (s, 1H), 4.98 (d, J = 0.9 Hz, 1H), 4.30 (d, J = 8.9 Hz, 1H), 4.00 (dd, J = 8.9, 0.8 Hz, 1H), 3.86 (s, 2H), 2.93 (d, J = 17.2 Hz, 1H), 2.70 (d, J = 18.6 Hz, 1H), 2.57 (d, J = 18.7 Hz, 1H), 2.28 (d, J = 17.2 Hz, 1H), 2.00 (s, 3H). ^{13}C NMR (101 MHz, $CDCl_3$) δ 204.8, 175.0, 170.5, 142.2, 136.7, 136.5, 129.2, 128.8, 127.4, 118.1, 76.4, 51.1, 44.4, 39.1, 37.8, 22.4.

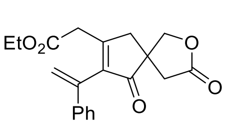

5e

Ethyl 2-(3,9-dioxo-8-(1-phenylvinyl)-2-oxaspiro[4.4]non-7-en-7-yl)acetate (**5e**). Colorless oil. Isolated yield 85%. ^{1}H NMR (400 MHz, $CDCl_3$) δ 7.34 – 7.28 (m, 3H), 7.22 (dd, J = 7.4, 2.3 Hz, 2H), 5.83 (s, 1H), 5.29 (s, 1H), 4.39 (d, J = 8.9 Hz, 1H), 4.27 – 4.18 (m, 1H), 4.12 (q, J = 7.1 Hz, 2H), 3.41 (d, J = 3.6 Hz, 2H), 3.13 – 3.03 (m, 2H), 3.02 – 2.90 (m, 2H), 2.48 (d, J = 17.9 Hz, 1H), 1.23 (t, J = 7.1 Hz, 3H). ^{13}C NMR (101 MHz, $CDCl_3$) δ 174.8, 168.5, 165.9, 138.6, 128.8, 128.5, 126.2, 119.1, 76.4, 61.7, 51.7, 45.3, 39.1, 37.1, 14.2.

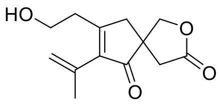

5f

8-(2-Hydroxyethyl)-7-(prop-1-en-2-yl)-2-oxaspiro[4.4]non-7-ene-3,6-dione (**5f**). Colorless oil. Isolated yield 57%. ^{1}H NMR (400 MHz, $CDCl_3$) δ 5.25 (s, 1H), 4.88 (d, *J* = 0.9 Hz, 1H), 4.34 (d, *J* = 8.8 Hz, 1H), 4.12 (d, *J* = 8.9 Hz, 1H), 3.90 (q, *J* = 6.1 Hz, 2H), 3.02 – 2.91 (m, 2H), 2.88 – 2.76 (m, 3H), 2.38 (d, *J* = 17.2 Hz, 1H), 1.93 (s, 3H), 1.55 (t, *J* = 4.9 Hz, 1H). ^{13}C NMR (101 MHz, $CDCl_3$) δ 204.7, 175.4, 170.5, 170.4, 143.1, 136.5, 117.9, 76.6, 60.4, 51.1, 45.1, 39.2, 34.5, 22.3.

Reference:

1 (a) Li, M.-B.; Posevins, D.; Geoffroy, A.; Zhu, C.; Bäckvall, J.-E. *Angew. Chem. Int. Ed.* **2020**, *59*, 1992-1996. (b) Li, M.-B.; Inge, A. K.; Posevins, D.; Gustafson, K. P. J.; Qiu, Y.; Bäckvall, J.-E., *J. Am. Chem. Soc.*, **2018**, *140*, 14604–14608.

2 Qiu, Y.; Yang, B.; Jiang, T.; Zhu, C.; Bäckvall, J.-E. *Angew. Chem. Int. Ed.* **2017**, *56*, 3221-3225.

1H-NMR and 13C-NMR spectrums for compounds

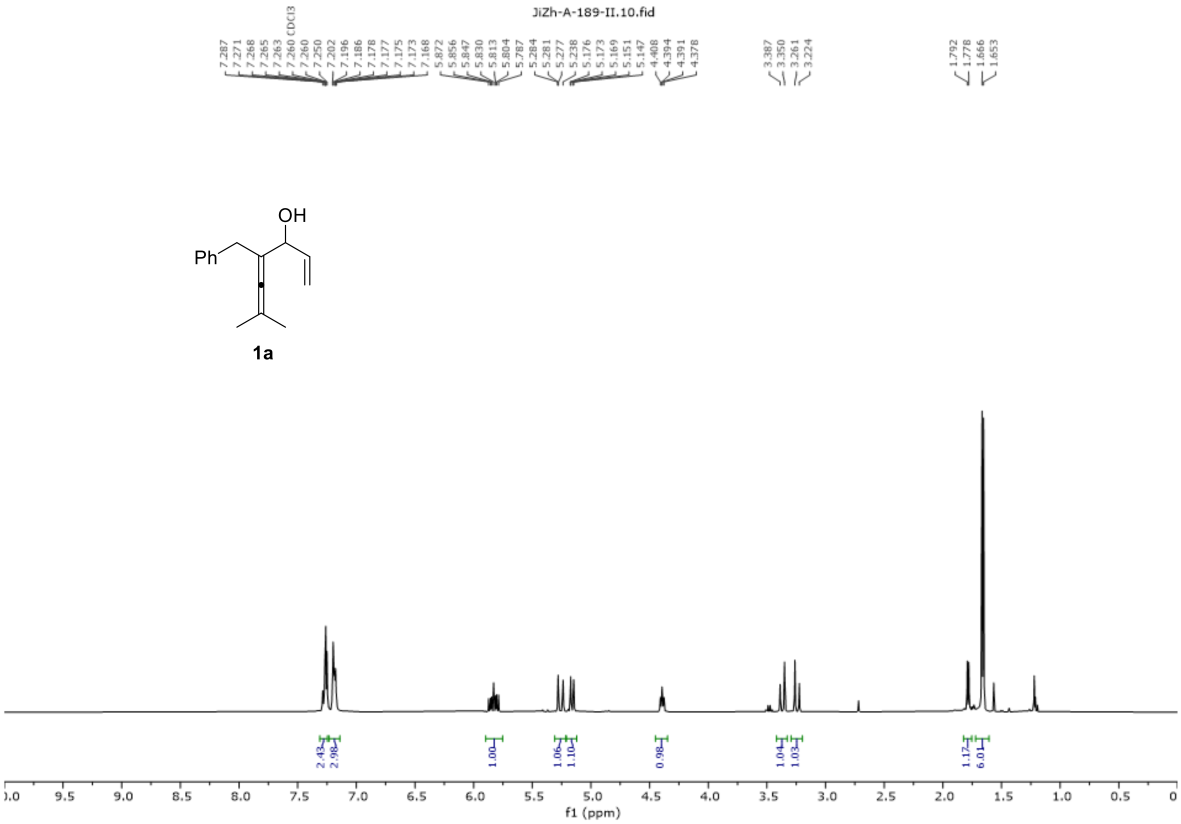

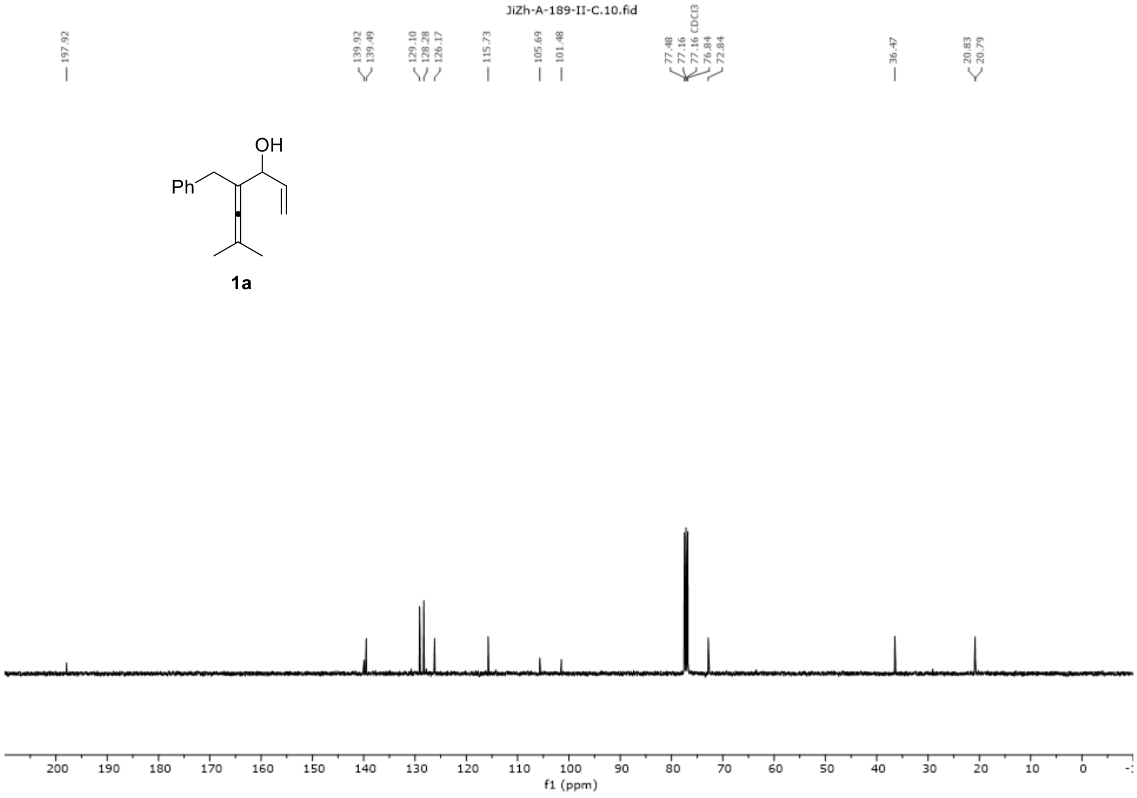

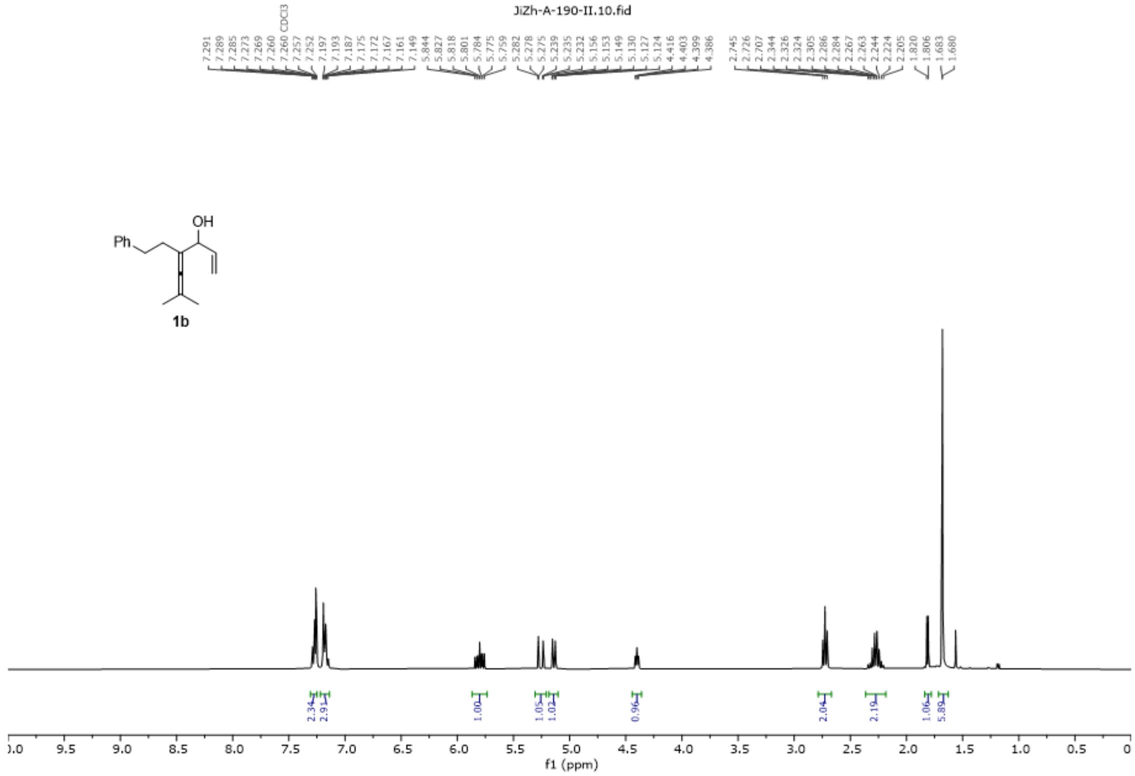
JiZh-A-190-II.10.fid
f1 (ppm)

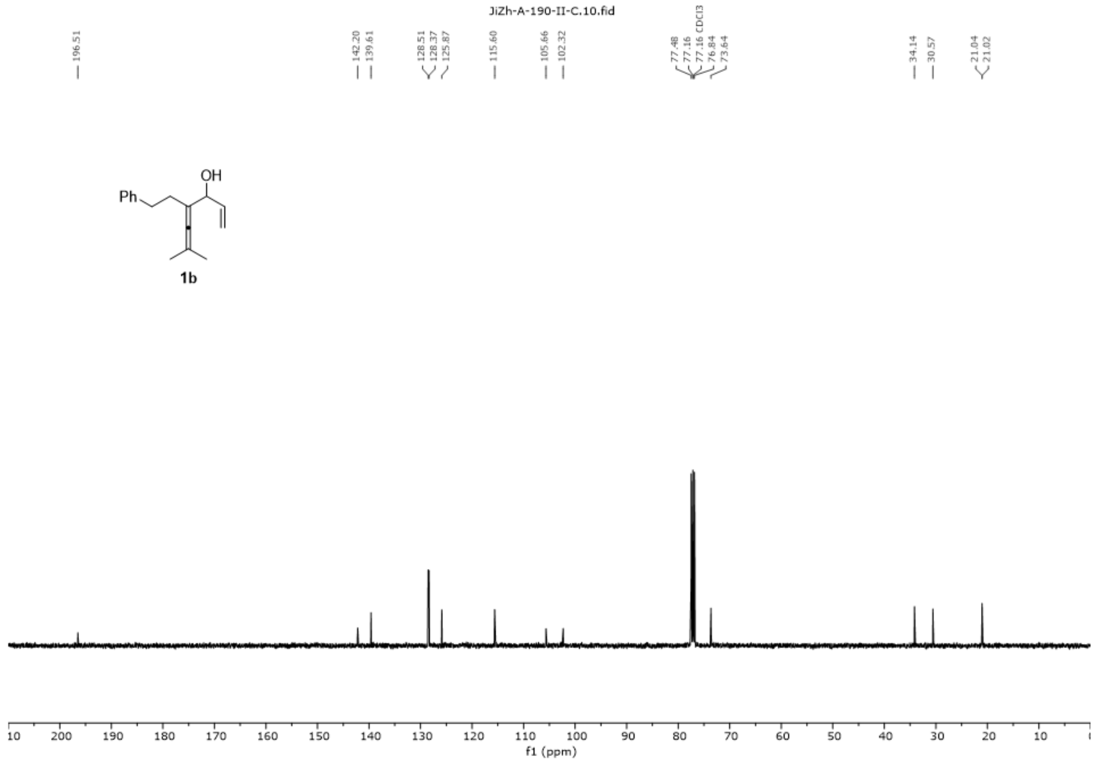
JiZh-A-190-II-C.10.fid
f1 (ppm)

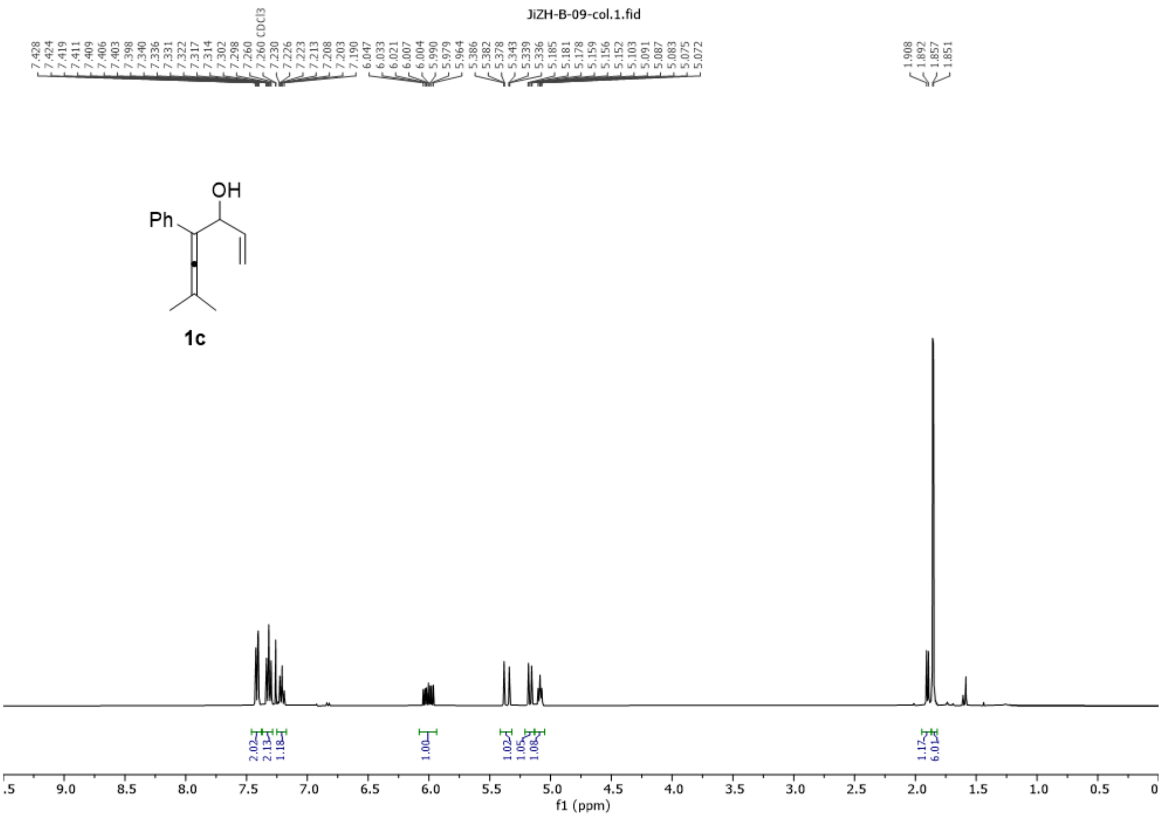

1c

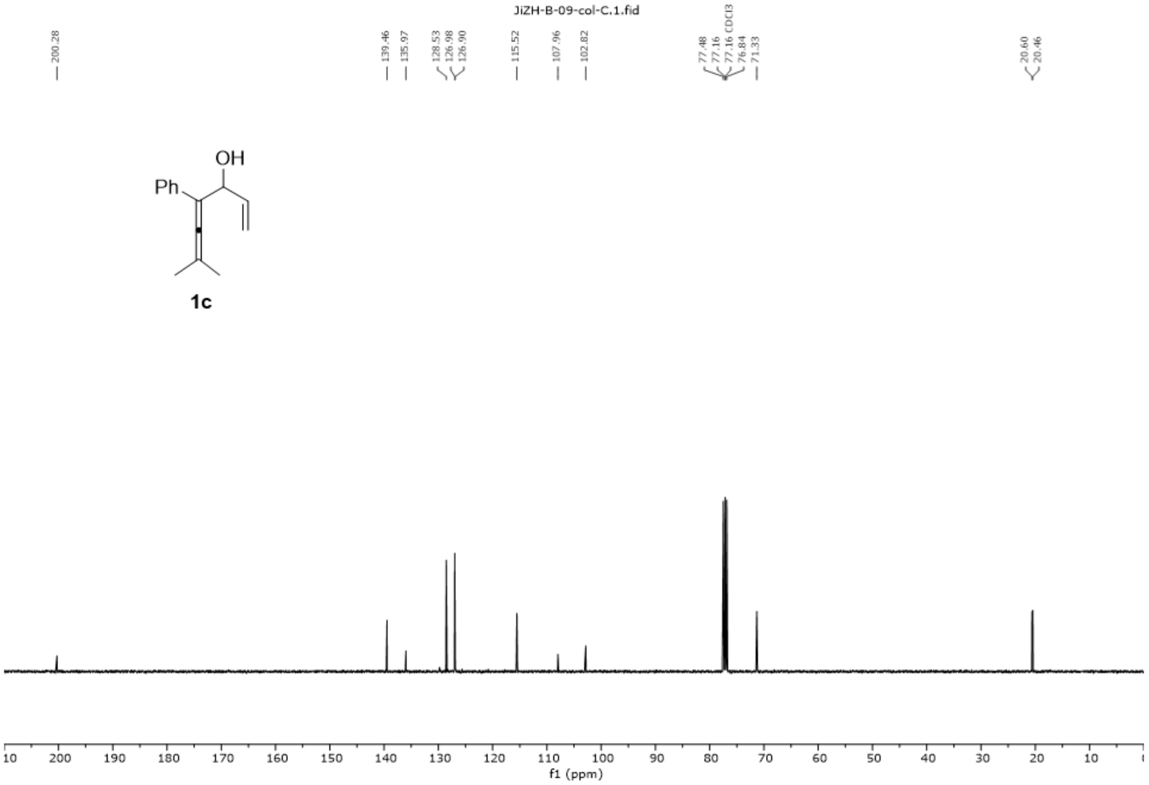

1c

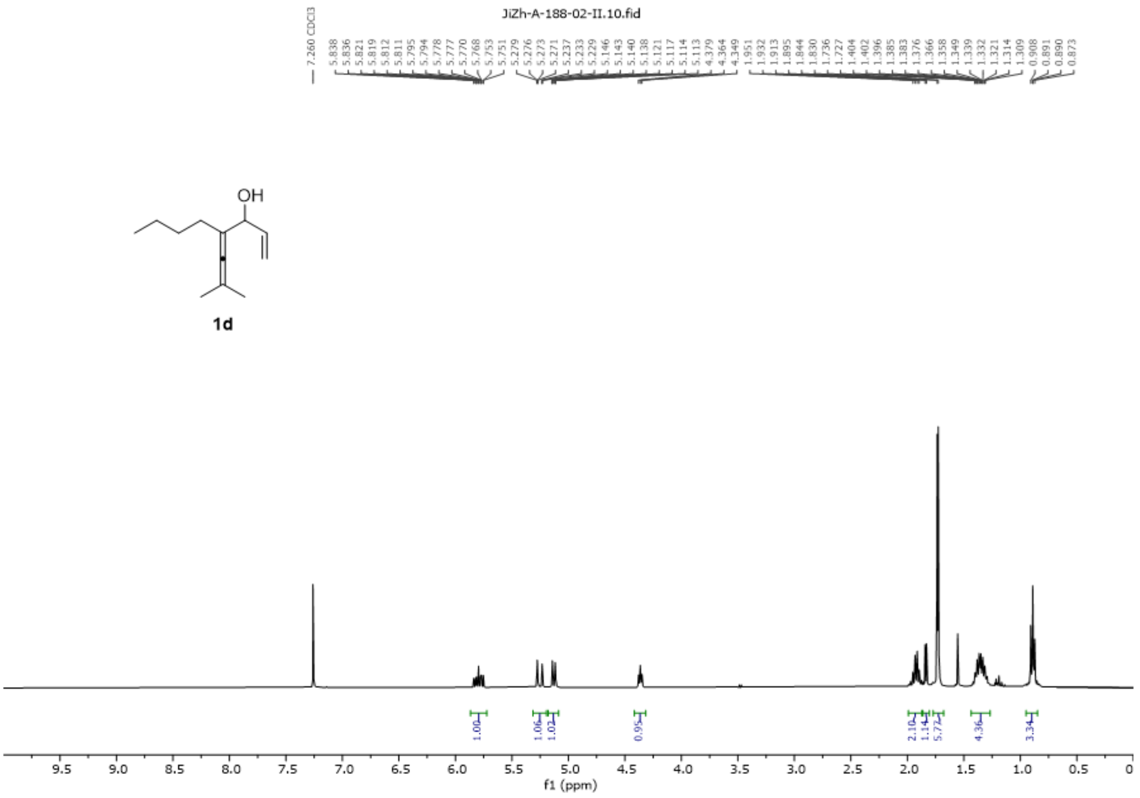
JiZh-A-188-02-II.10.fid
7.260 CDCl3
5.838 5.836 5.821 5.819 5.812 5.811 5.795 5.794 5.778 5.777 5.770 5.768 5.753 5.751
5.279 5.276 5.273 5.271 5.237 5.233 5.229 5.146 5.143 5.140 5.138 5.121 5.117 5.114 5.113 4.379 4.364 4.349
1.951 1.932 1.913 1.895 1.844 1.830 1.736 1.727 1.404 1.402 1.396 1.385 1.383 1.376 1.366 1.358 1.349 1.339 1.332 1.321 1.314 1.309 0.908 0.891 0.890 0.873
OH
1d
1.00 1.06 1.02 0.95 2.10 1.14 5.72 4.36 3.34
f1 (ppm)

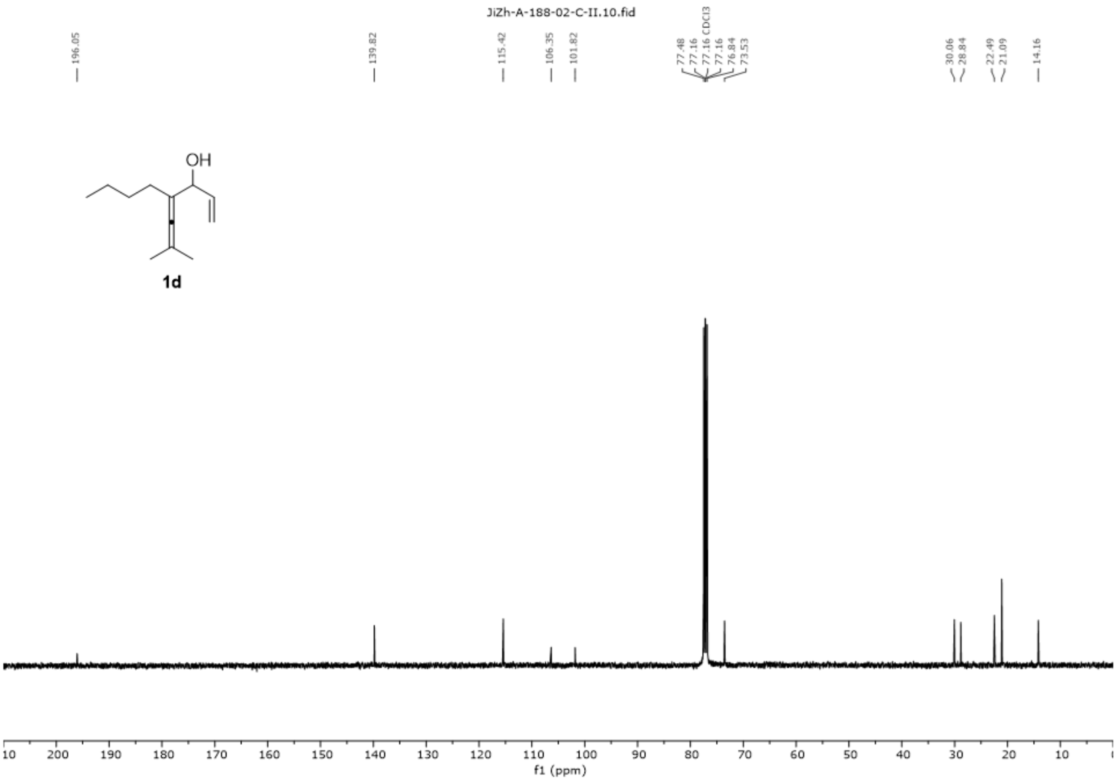
JiZh-A-188-02-C-II.10.fid
196.05
139.82
115.42
106.35
101.82
77.48 77.16 CDCl3 77.16 76.84 73.53
30.06 28.84
22.49 21.09
14.16
OH
1d
f1 (ppm)

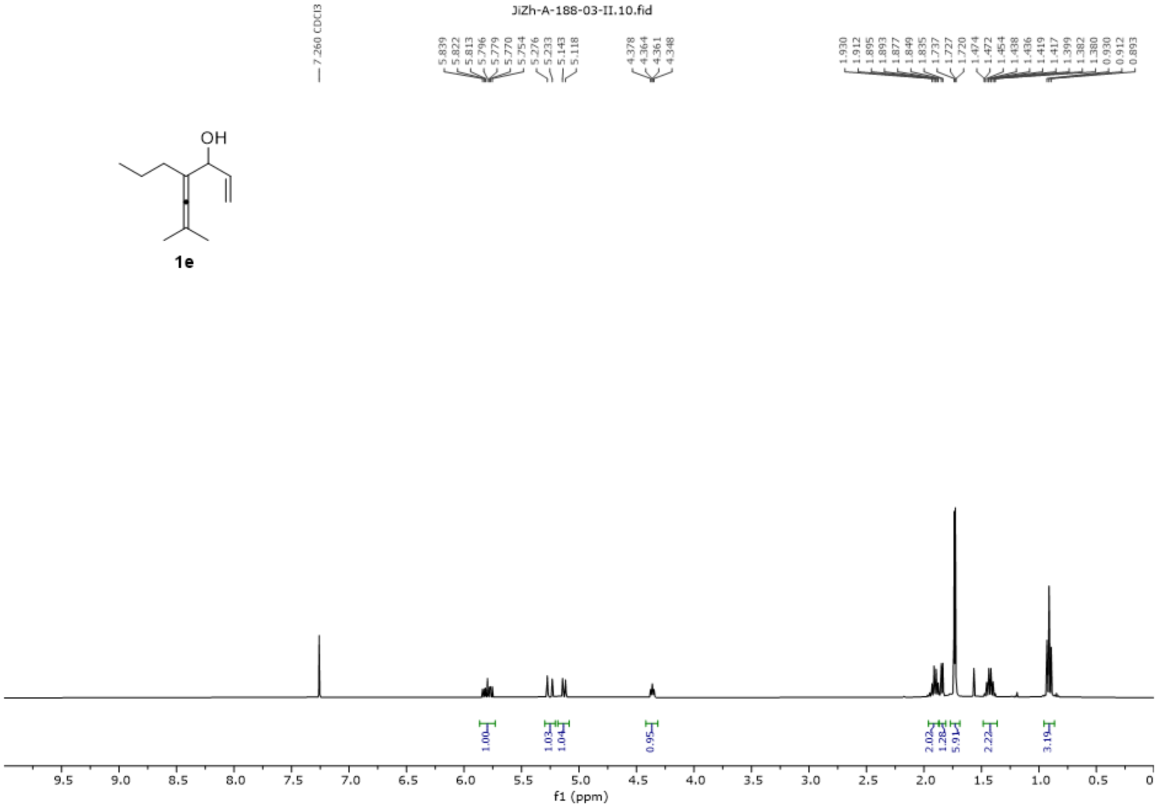
JiZh-A-188-03-II.10.fid
7.260 CDCl3
5.839 5.822 5.813 5.796 5.779 5.770 5.754 5.276 5.233 5.143 5.118
4.378 4.364 4.361 4.348
1.930 1.912 1.895 1.893 1.877 1.849 1.835 1.737 1.727 1.720 1.474 1.472 1.454 1.438 1.436 1.419 1.417 1.399 1.382 1.380 0.930 0.912 0.893
OH
1e
1.00 1.03 1.04 0.95 2.02 1.28 5.91 2.22 3.19
f1 (ppm)

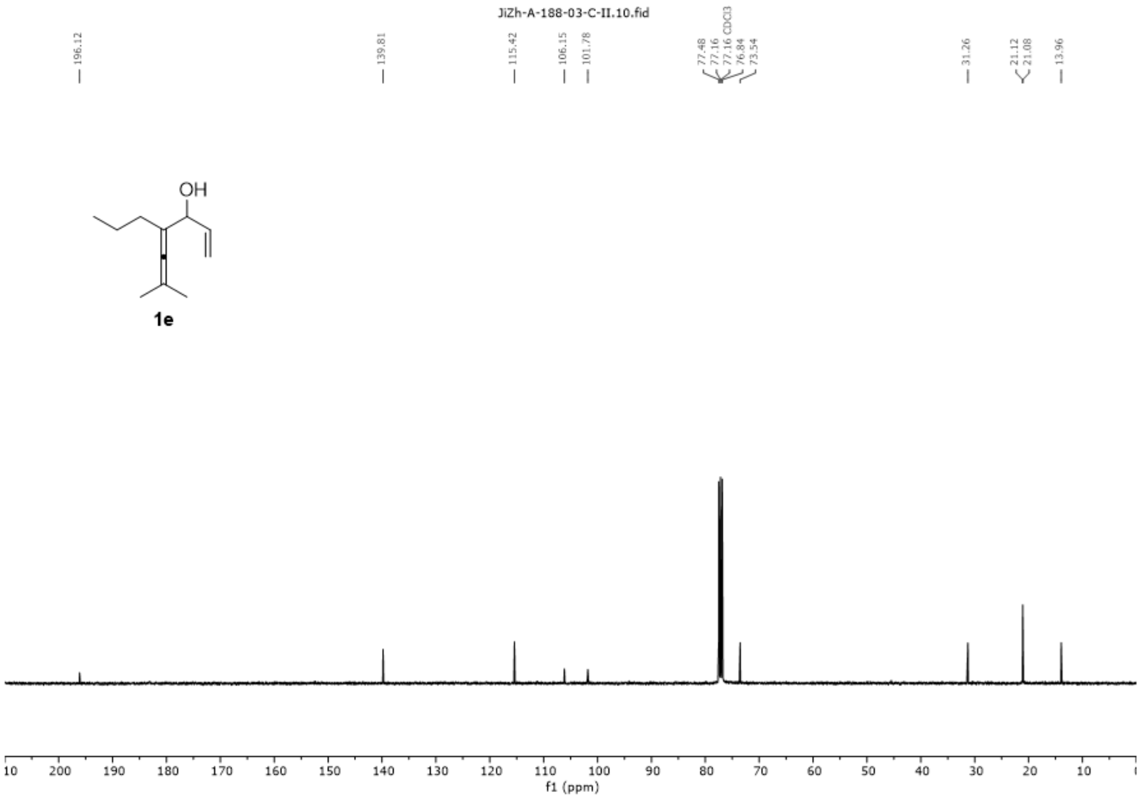
JiZh-A-188-03-C-II.10.fid
196.12
139.81
115.42
106.15
101.78
77.48 77.16 77.16 CDCl3 76.84 73.54
31.26
21.12 21.08
13.96
OH
1e
f1 (ppm)

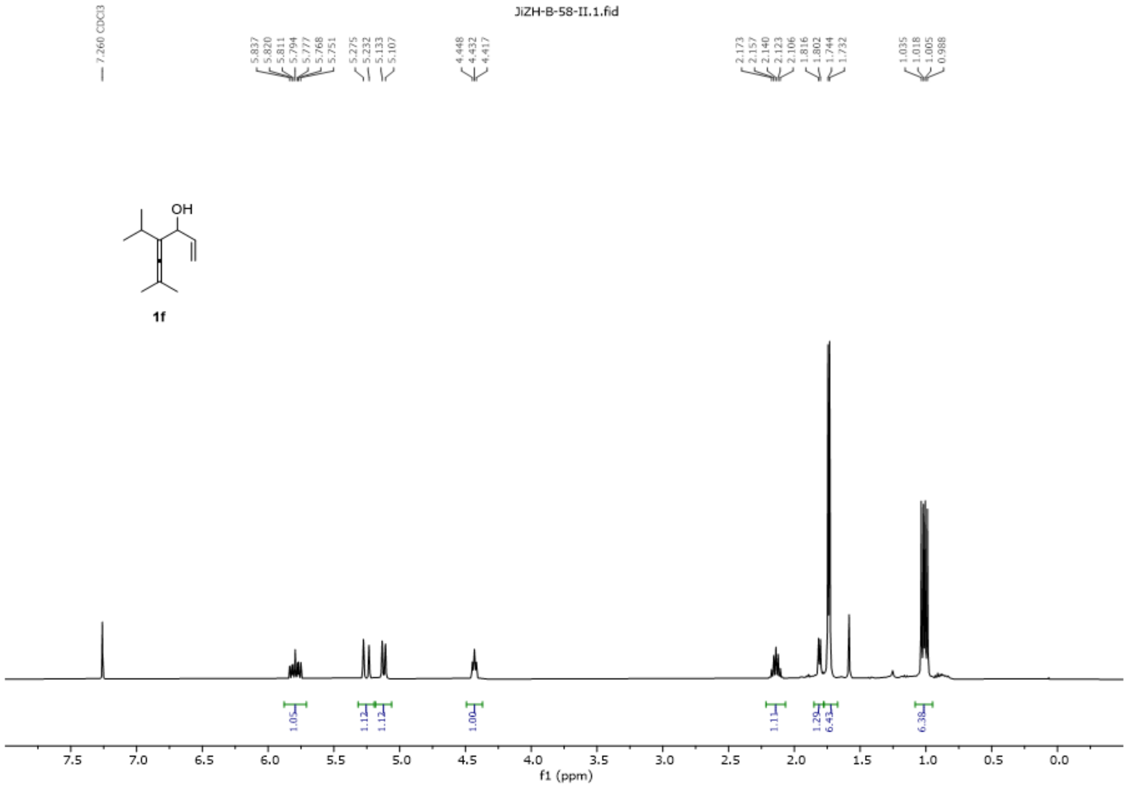
JiZH-B-58-II.1.fid
OH
1f
f1 (ppm)

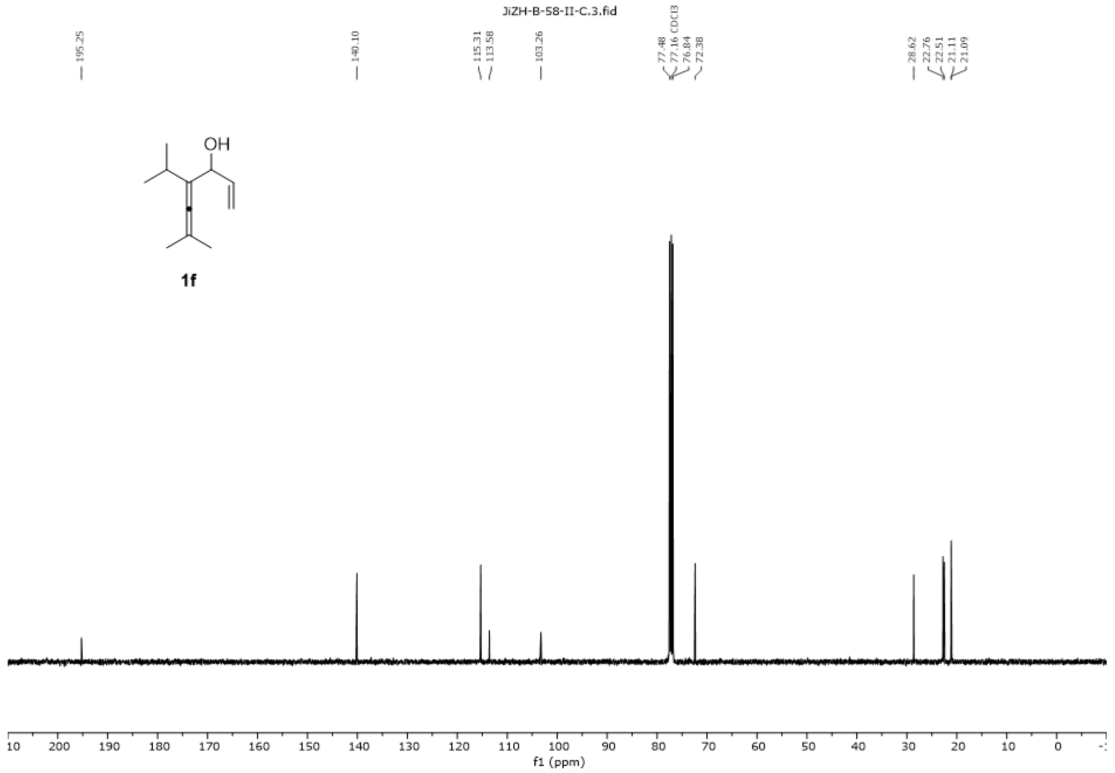
JiZH-B-58-II-C.3.fid
OH
1f
f1 (ppm)

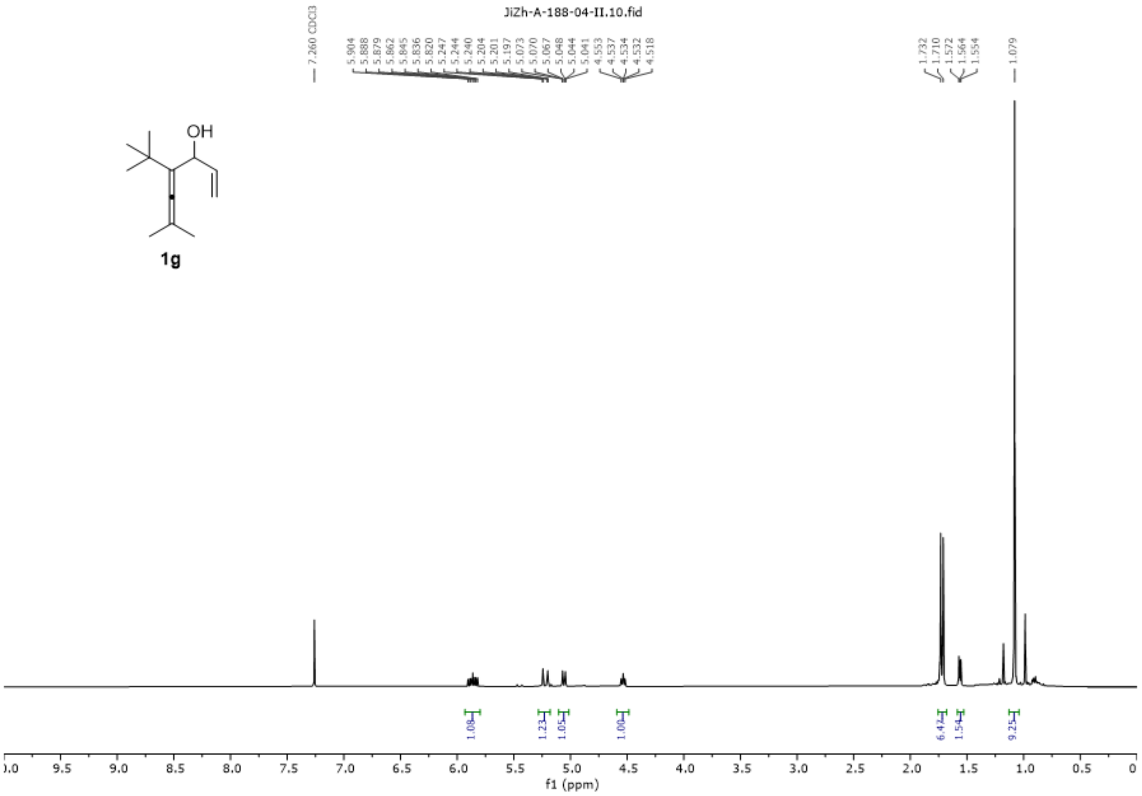
JiZh-A-188-04-II.10.fid
7.260 CDCl3
5.904 5.888 5.879 5.862 5.845 5.836 5.820 5.247 5.244 5.240 5.204 5.201 5.197 5.073 5.070 5.067 5.048 5.044 5.041 4.553 4.537 4.534 4.532 4.518
1.732 1.710 1.572 1.564 1.554 1.079
OH
1g
1.08 1.23 1.05 1.00 6.47 1.54 9.25
f1 (ppm)

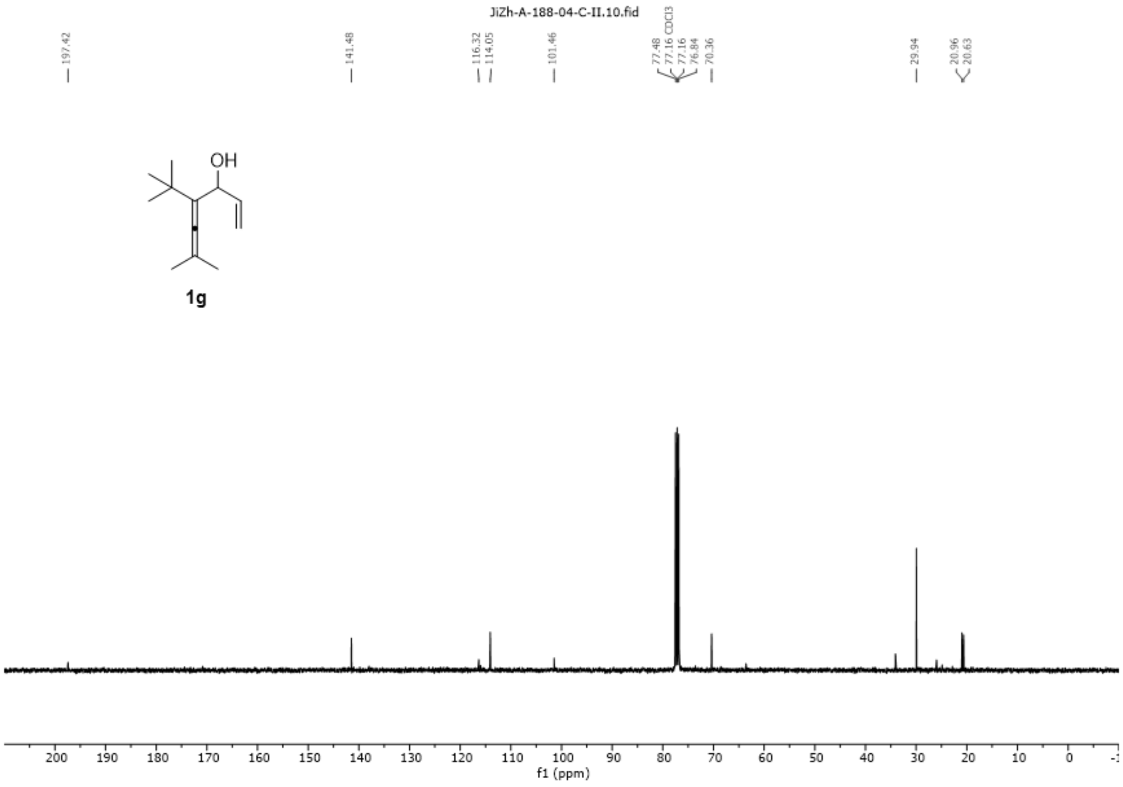
JiZh-A-188-04-C-II.10.fid
197.42 141.48 116.32 114.05 101.46 77.48 77.16 CDCl3 77.16 76.84 70.36 29.94 20.96 20.63
OH
1g
f1 (ppm)

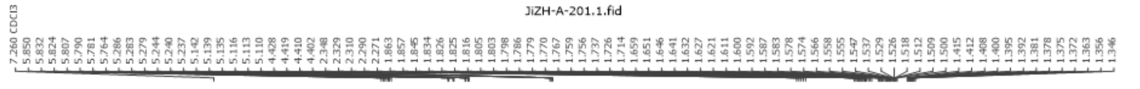
JiZH-A-201.1.fid
7.260 CDCl3
5.850
5.832
5.824
5.807
5.790
5.781
5.764
5.286
5.283
5.279
5.244
5.240
5.237
5.142
5.139
5.135
5.116
5.113
5.110
4.428
4.419
4.410
4.402
2.348
2.329
2.310
2.290
2.271
1.863
1.857
1.845
1.834
1.826
1.825
1.816
1.805
1.803
1.798
1.786
1.779
1.770
1.767
1.759
1.756
1.737
1.726
1.714
1.659
1.651
1.646
1.641
1.632
1.627
1.621
1.611
1.600
1.592
1.587
1.583
1.578
1.574
1.566
1.558
1.555
1.547
1.537
1.529
1.526
1.518
1.512
1.509
1.500
1.415
1.412
1.408
1.400
1.395
1.392
1.381
1.378
1.375
1.372
1.363
1.356
1.346

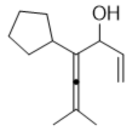
OH

1h

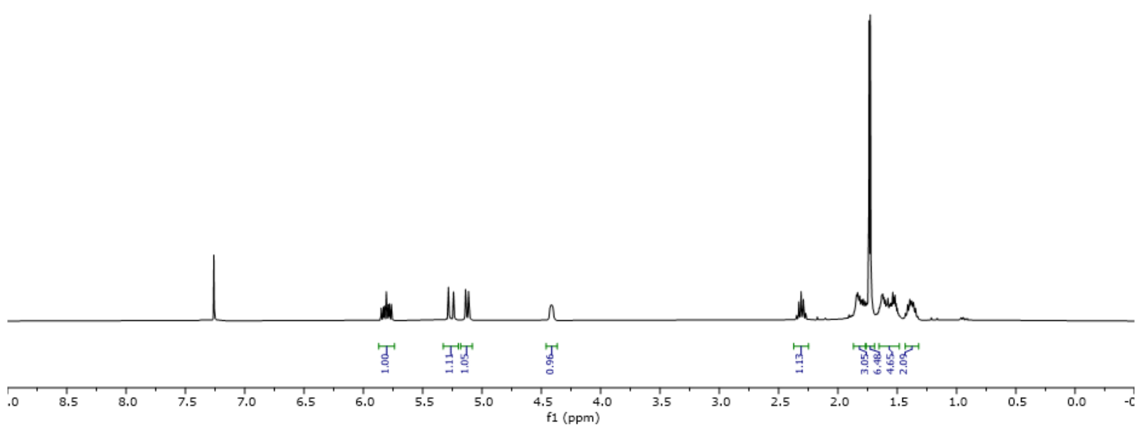
1.00
1.11
1.05
0.96
1.13
3.05
6.48
4.65
2.09
8.5
8.0
7.5
7.0
6.5
6.0
5.5
5.0
4.5
4.0
3.5
3.0
2.5
2.0
1.5
1.0
0.5
0.0
f1 (ppm)

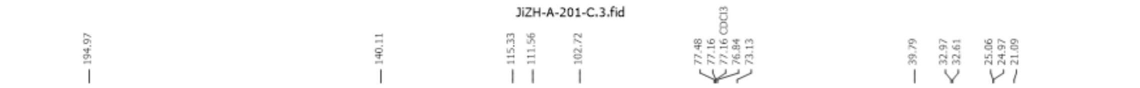
JiZH-A-201-C.3.fid
194.97
140.11
115.33
111.56
102.72
77.48
77.16 CDCl3
76.84
73.13
39.79
32.97
32.61
25.06
24.97
21.09

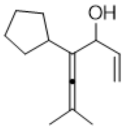
OH

1h

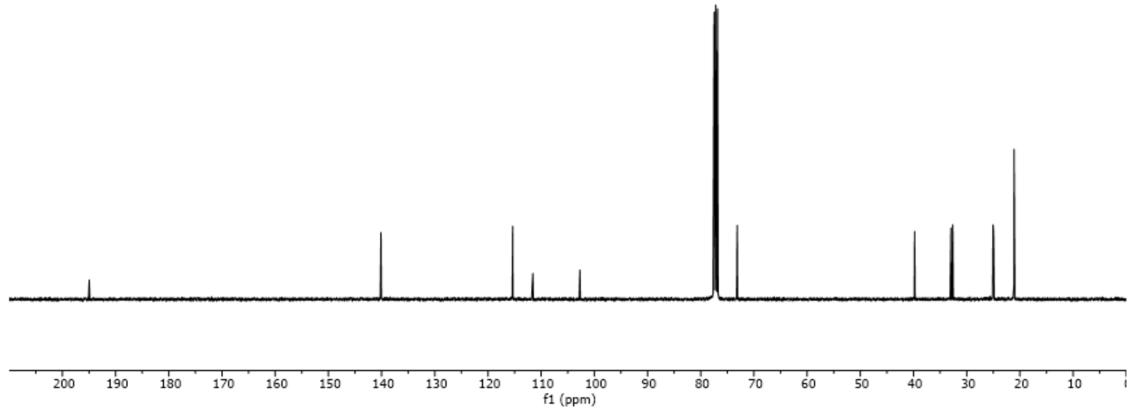
200
190
180
170
160
150
140
130
120
110
100
90
80
70
60
50
40
30
20
10
f1 (ppm)

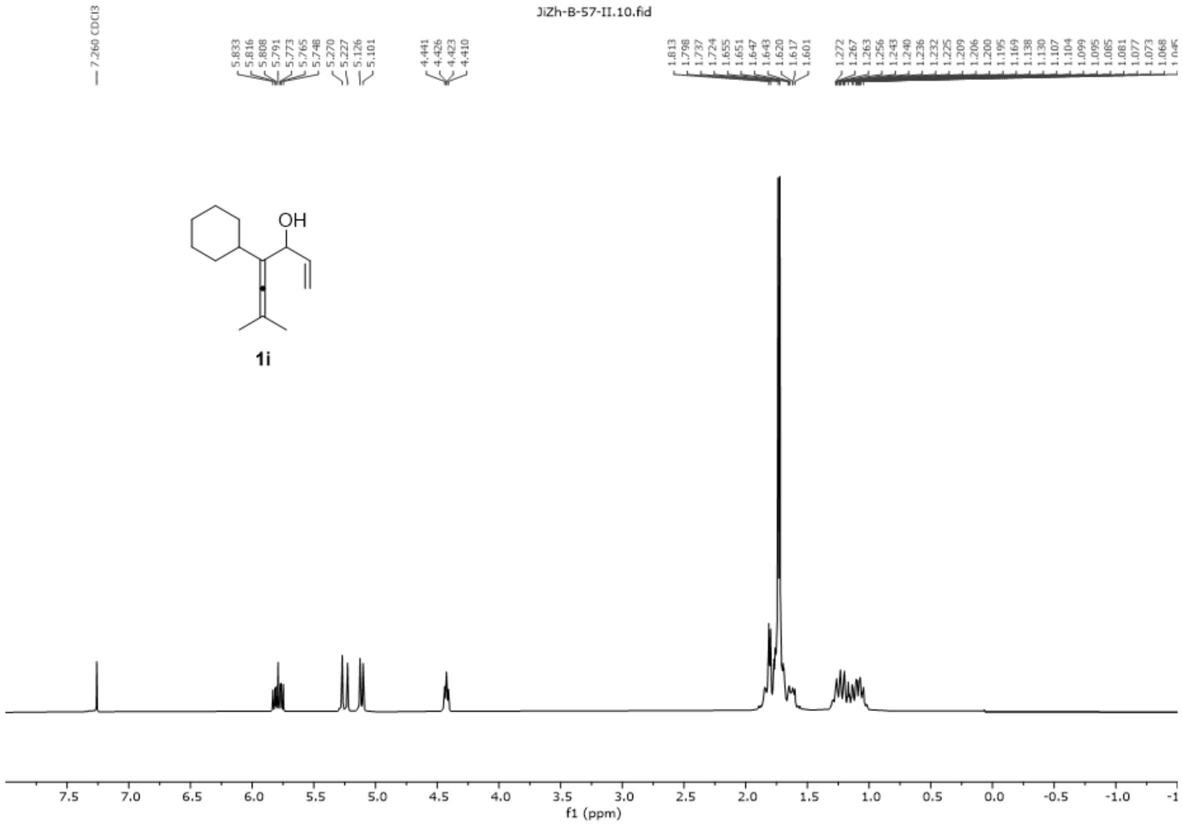
JiZh-B-57-II.10.fid
7.260 CDCl3
5.833
5.816
5.808
5.791
5.773
5.765
5.748
5.270
5.227
5.126
5.101
4.441
4.426
4.423
4.410
1.813
1.798
1.737
1.724
1.655
1.651
1.647
1.643
1.620
1.617
1.601
1.272
1.267
1.263
1.256
1.243
1.240
1.236
1.232
1.225
1.209
1.206
1.200
1.195
1.169
1.138
1.130
1.107
1.104
1.099
1.095
1.085
1.081
1.077
1.073
1.068
OH
1i
f1 (ppm)

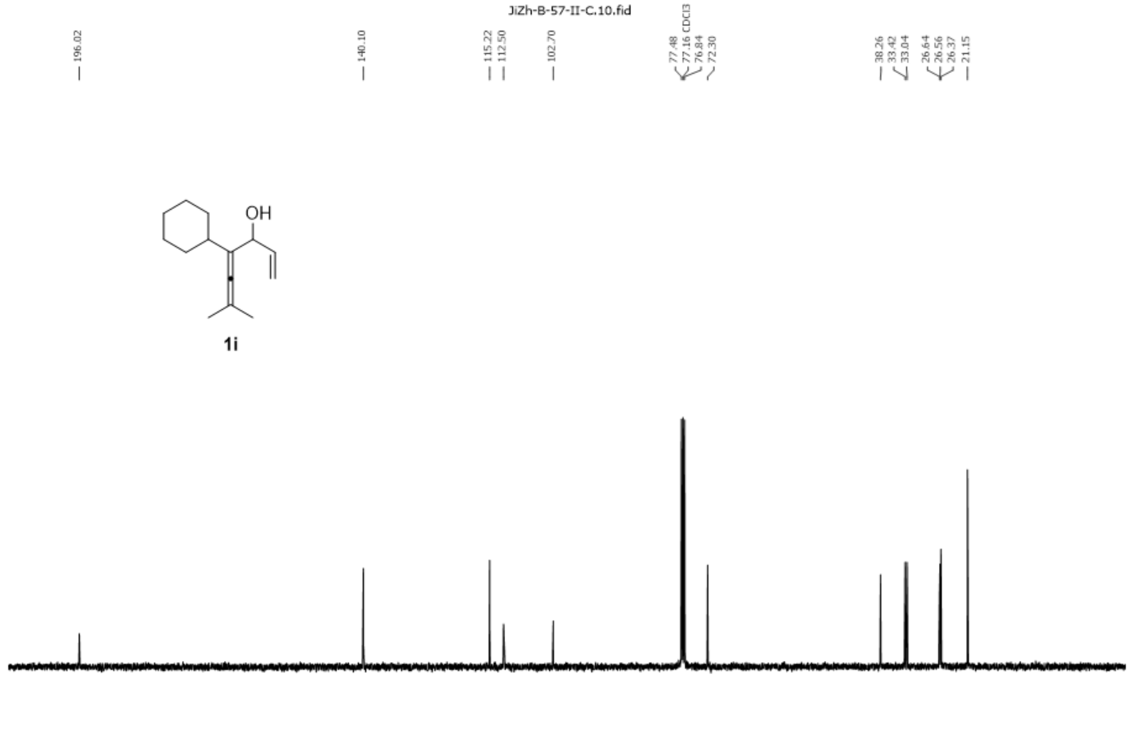
JiZh-B-57-II-C.10.fid
196.02
140.10
115.22
112.50
102.70
77.48
77.16 CDCl3
76.84
72.30
38.26
33.42
33.04
26.64
26.56
26.37
21.15
OH
1i
f1 (ppm)

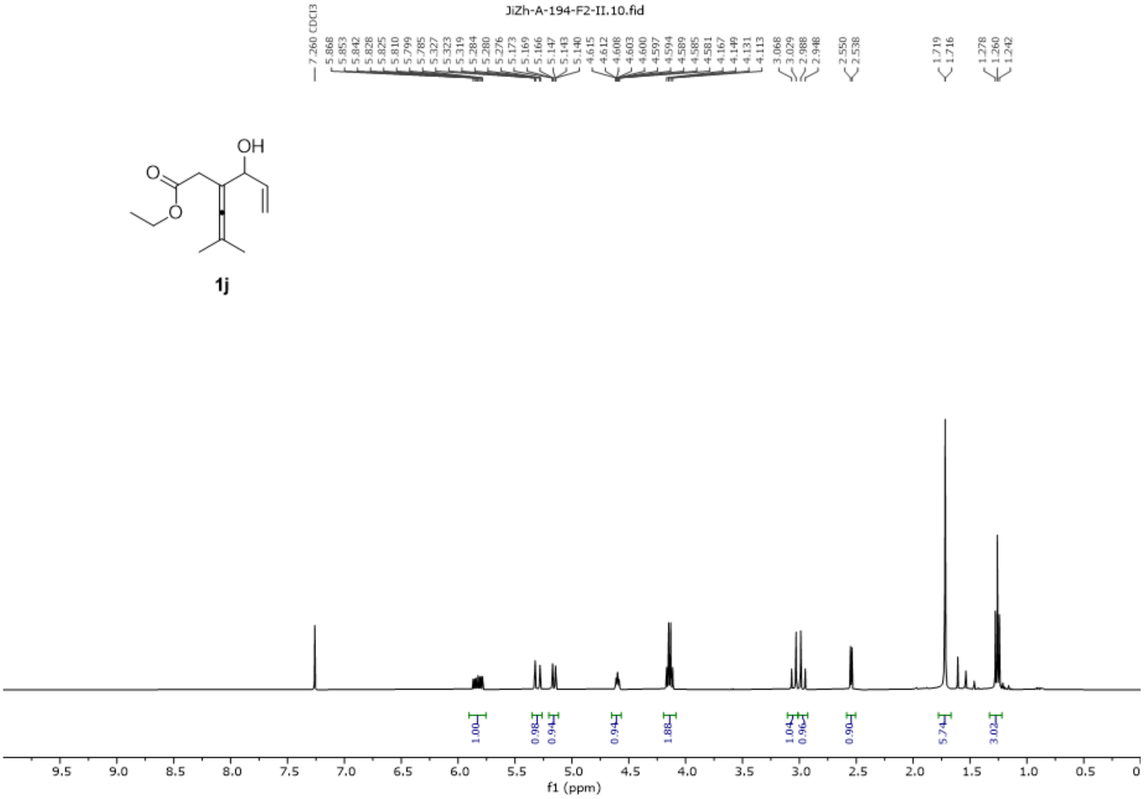
JiZh-A-194-F2-II.10.fid
7.260 CDCl3
5.868 5.853 5.842 5.828 5.825 5.810 5.799 5.785 5.327 5.323 5.319 5.284 5.280 5.276 5.173 5.169 5.166 5.147 5.143 5.140 4.615 4.612 4.608 4.603 4.600 4.597 4.594 4.589 4.585 4.581 4.167 4.149 4.131 4.113 3.068 3.029 2.988 2.948 2.550 2.538 1.719 1.716 1.278 1.260 1.242
OH
O
O
1j
1.00 0.98 0.94 0.94 1.88 1.04 0.96 0.90 5.74 3.02
9.5 9.0 8.5 8.0 7.5 7.0 6.5 6.0 5.5 5.0 4.5 4.0 3.5 3.0 2.5 2.0 1.5 1.0 0.5 0
f1 (ppm)

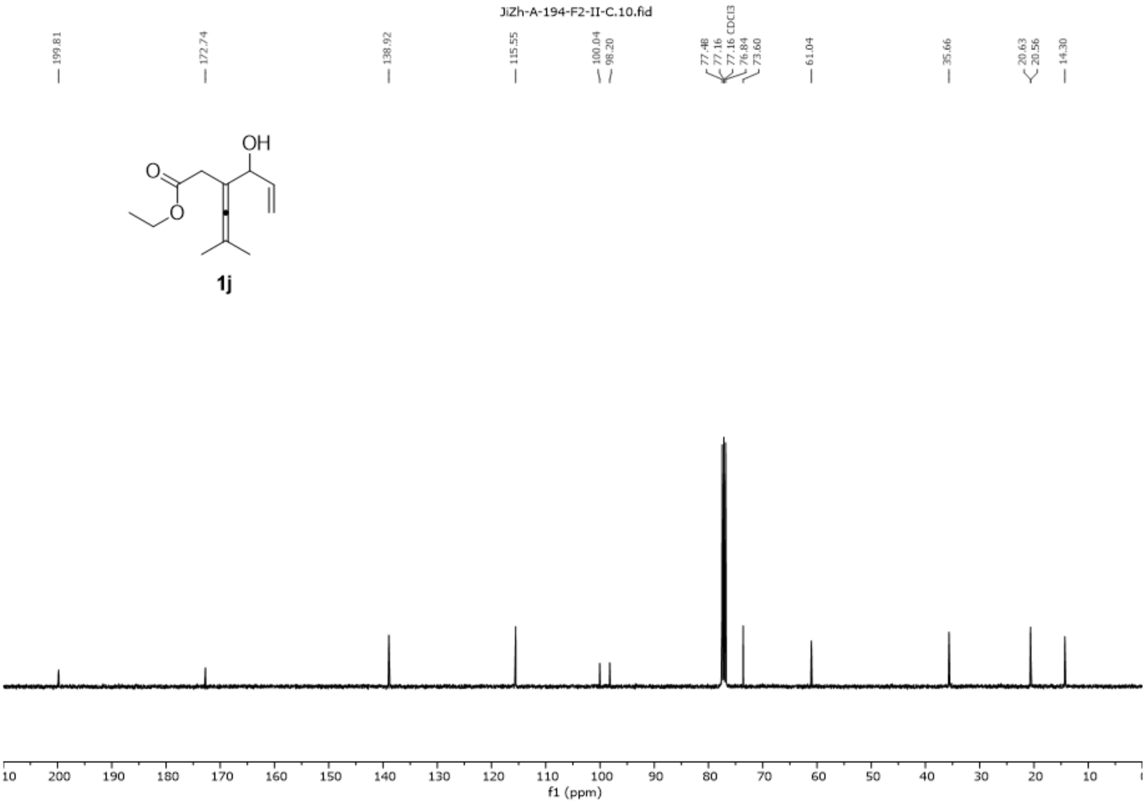
JiZh-A-194-F2-II-C.10.fid
199.81 172.74 138.92 115.55 100.04 98.20 77.48 77.16 77.16 CDCl3 76.84 73.60 61.04 35.66 20.63 20.56 14.30
OH
O
O
1j
10 200 190 180 170 160 150 140 130 120 110 100 90 80 70 60 50 40 30 20 10
f1 (ppm)

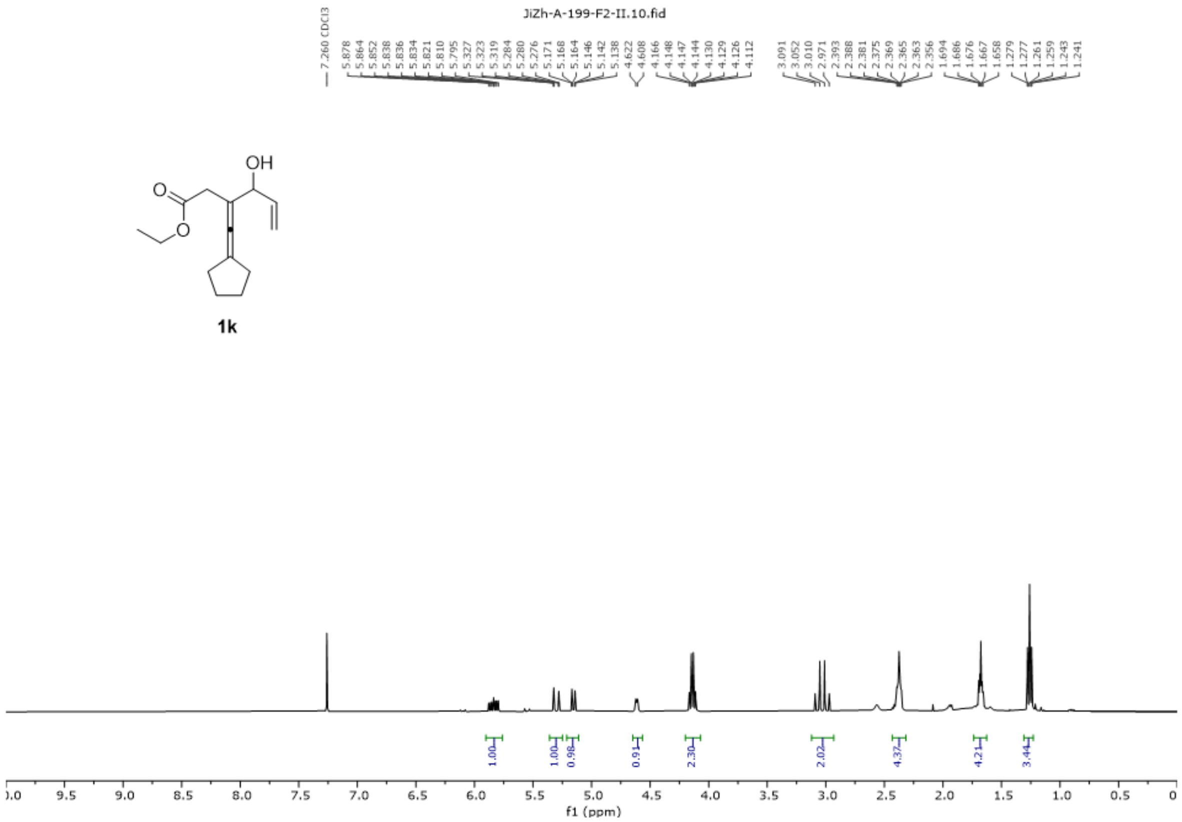
JiZh-A-199-F2-II.10.fid
7.260 CDCl3
5.878 5.864 5.852 5.838 5.836 5.834 5.821 5.810 5.795 5.327 5.323 5.319 5.284 5.280 5.276 5.171 5.168 5.164 5.146 5.142 5.138 4.622 4.608 4.166 4.148 4.147 4.144 4.130 4.129 4.126 4.112 3.091 3.052 3.010 2.971 2.393 2.388 2.381 2.375 2.369 2.365 2.363 2.356 1.694 1.686 1.676 1.667 1.658 1.279 1.277 1.261 1.259 1.243 1.241
OH
O
O
1k
1.00 1.00 0.98 0.91 2.30 2.02 4.37 4.21 3.44
f1 (ppm)

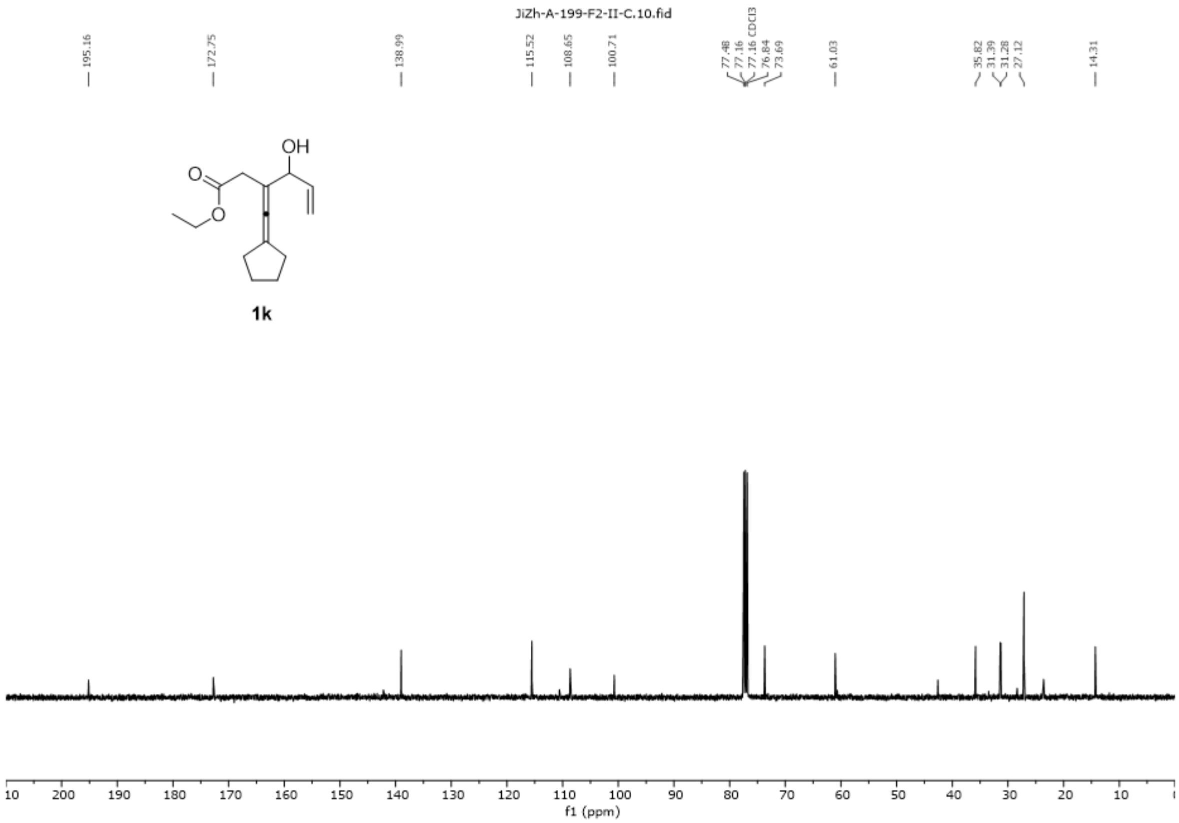
JiZh-A-199-F2-II-C.10.fid
195.16
172.75
138.99
115.52
108.65
100.71
77.48
77.16 CDCl3
76.84
73.69
61.03
35.82
31.39
31.28
27.12
14.31
OH
O
O
1k
f1 (ppm)

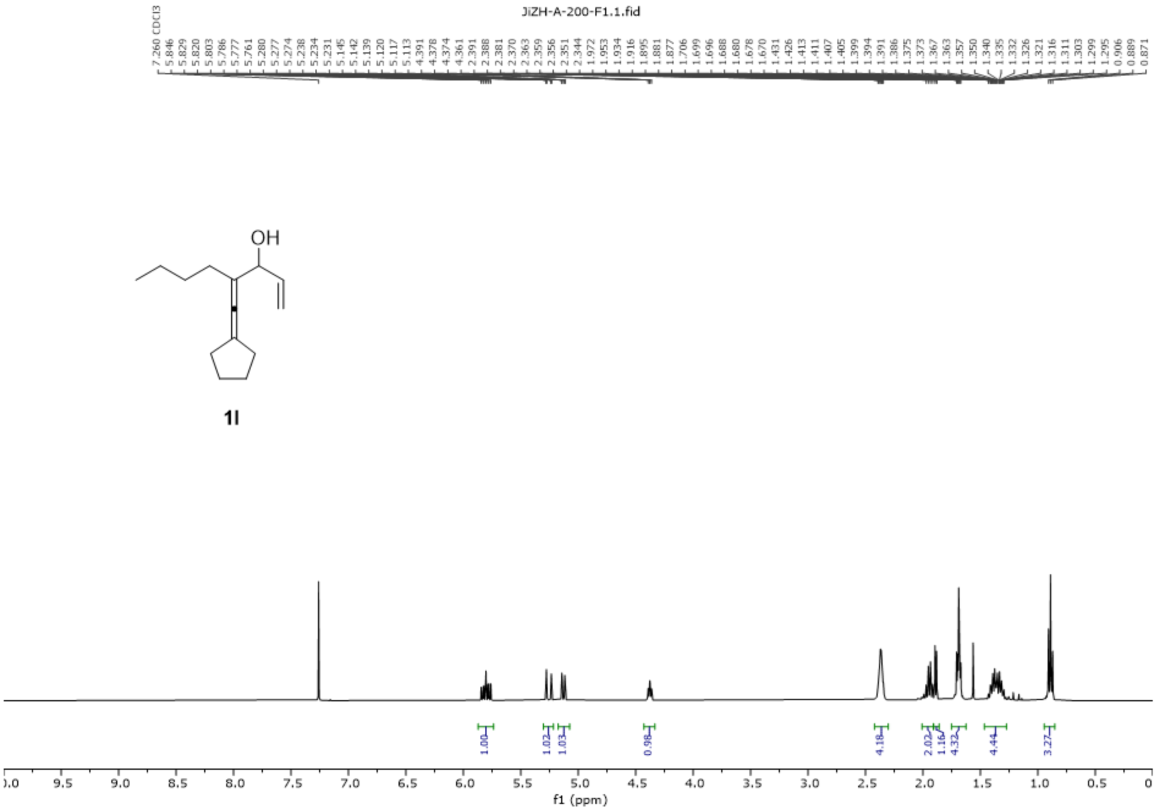
JiZH-A-200-F1.1.fid
7.260 CDCl3
5.846
5.829
5.820
5.803
5.786
5.777
5.761
5.280
5.277
5.274
5.238
5.234
5.231
5.145
5.142
5.139
5.120
5.117
5.113
4.391
4.378
4.374
4.361
2.391
2.388
2.381
2.370
2.363
2.359
2.356
2.351
2.344
1.972
1.953
1.934
1.916
1.895
1.881
1.877
1.706
1.699
1.696
1.688
1.680
1.678
1.670
1.431
1.426
1.413
1.411
1.407
1.405
1.399
1.394
1.391
1.386
1.375
1.373
1.367
1.363
1.357
1.350
1.340
1.335
1.332
1.326
1.321
1.316
1.311
1.303
1.299
1.295
0.906
0.889
0.871
1.00
1.02
1.03
0.98
4.18
2.02
1.16
4.32
4.44
3.27
f1 (ppm)

1l

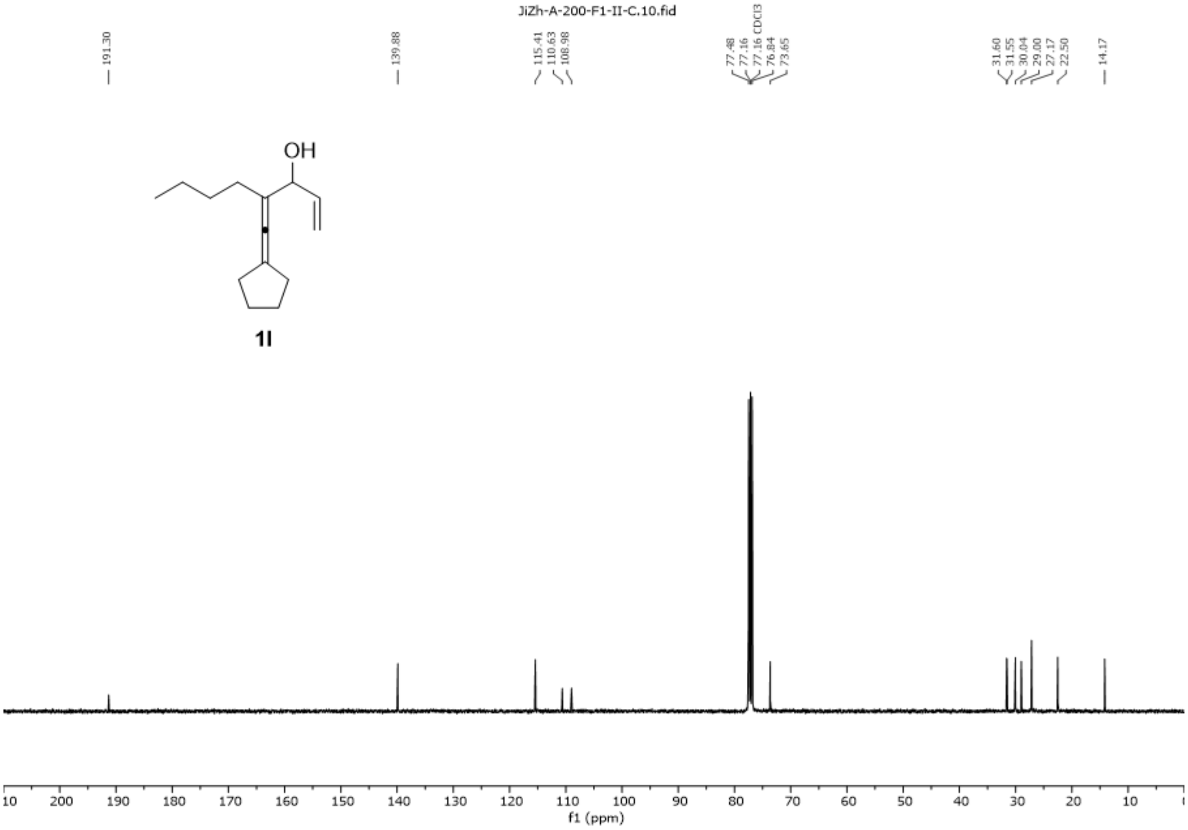
JiZh-A-200-F1-II-C.10.fid
191.30
139.88
115.41
110.63
108.98
77.48
77.16
77.16 CDCl3
76.84
73.65
31.60
31.55
30.04
29.00
27.17
22.50
14.17
f1 (ppm)

1l

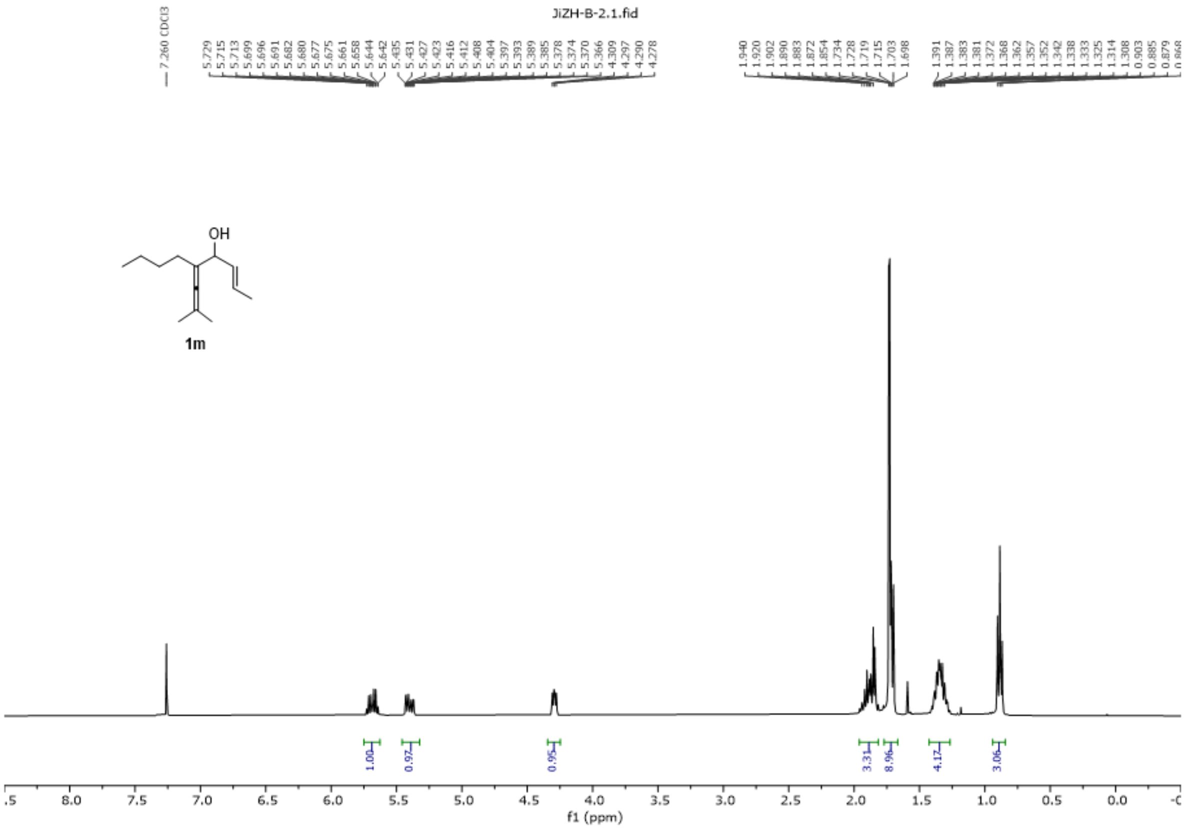
JiZH-B-2.1.fid
7.260 CDCl3
5.729
5.715
5.713
5.699
5.696
5.691
5.682
5.680
5.677
5.675
5.661
5.658
5.644
5.642
5.435
5.431
5.427
5.423
5.416
5.412
5.408
5.404
5.397
5.393
5.389
5.385
5.378
5.374
5.370
5.366
4.309
4.297
4.290
4.278
1.940
1.920
1.902
1.890
1.883
1.872
1.854
1.734
1.728
1.719
1.715
1.703
1.698
1.391
1.387
1.383
1.381
1.372
1.368
1.362
1.357
1.352
1.342
1.338
1.333
1.325
1.314
1.308
0.903
0.885
0.879
OH
1m
1.00
0.97
0.95
3.31
8.96
4.17
3.06
f1 (ppm)

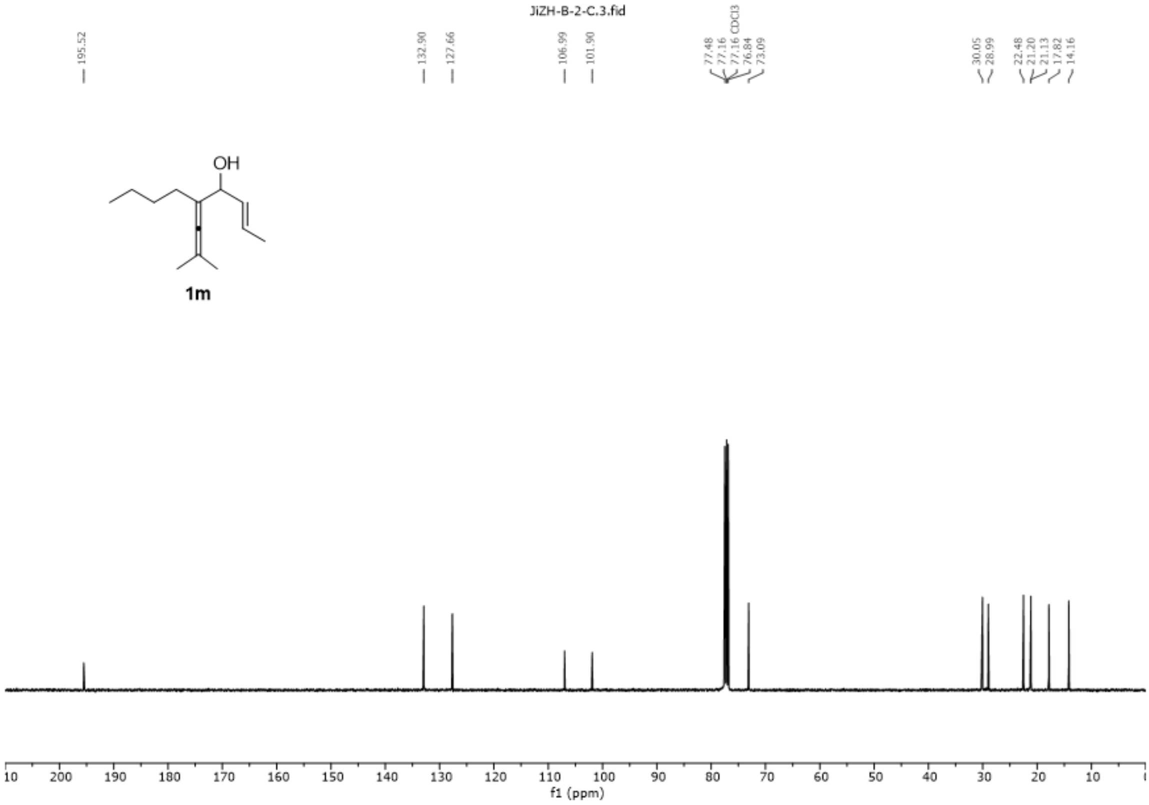
JiZH-B-2-C.3.fid
195.52
132.90
127.66
106.99
101.90
77.48
77.16
77.16 CDCl3
76.84
73.09
30.05
28.99
22.48
21.20
21.13
17.82
14.16
OH
1m
f1 (ppm)

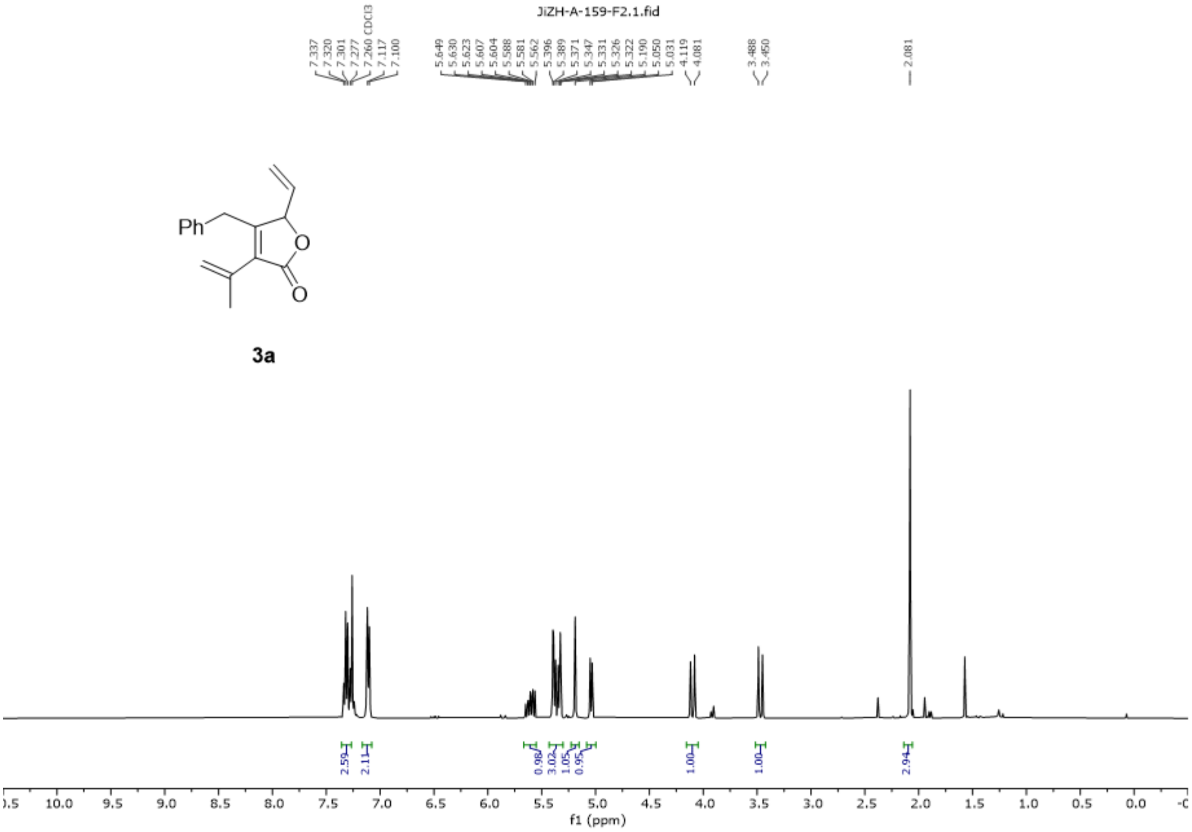
JiZH-A-159-F2.1.fid
7.337 7.320 7.301 7.277 7.260 CDCl3 7.117 7.100
5.649 5.630 5.623 5.607 5.604 5.588 5.581 5.562 5.396 5.389 5.371 5.347 5.331 5.326 5.322 5.190 5.050 5.031 4.119 4.081
3.488 3.450
2.081
Ph
O
O
3a
2.59 2.11 0.98 3.02 1.05 0.95 1.00 1.00 2.94
f1 (ppm)

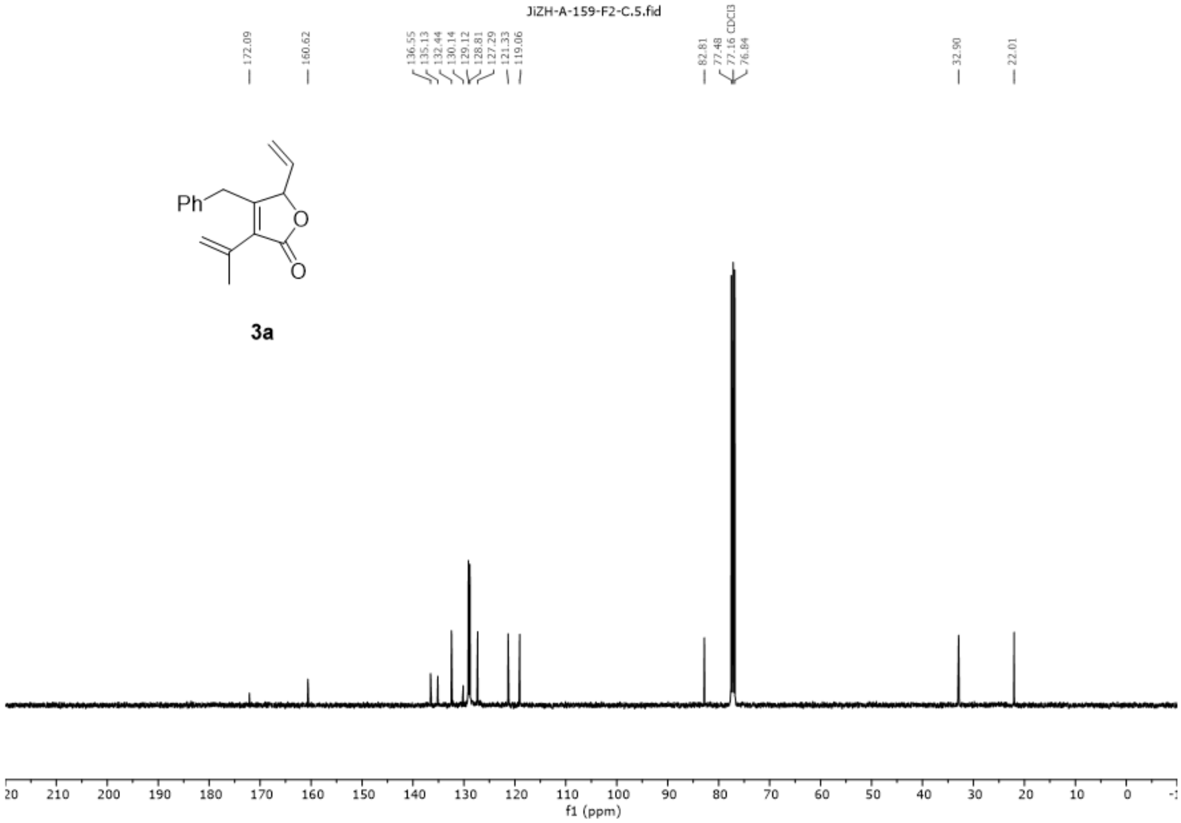
JiZH-A-159-F2-C.5.fid
172.09
160.62
136.55 135.13 132.44 130.14 129.12 128.81 127.29 121.33 119.06
82.81 77.48 77.16 CDCl3 76.84
32.90
22.01
Ph
O
O
3a
f1 (ppm)

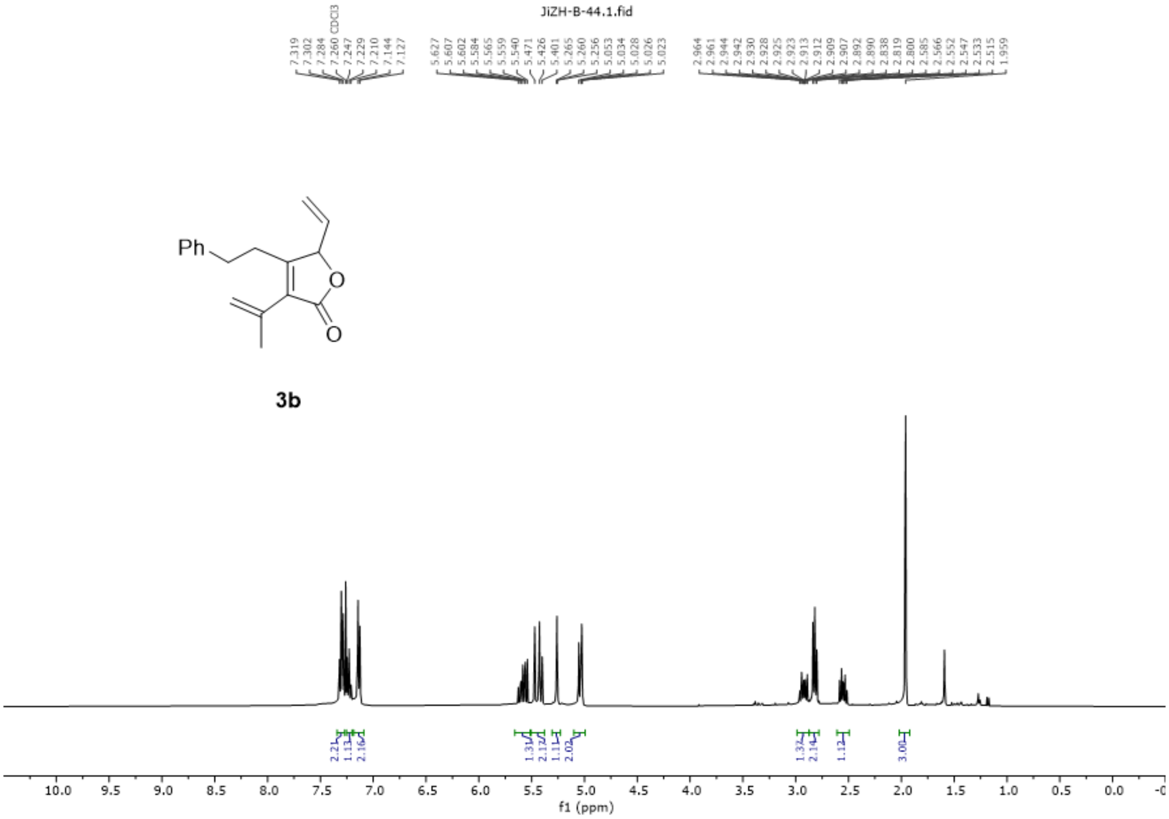
JiZH-B-44.1.fid
7.319 7.302 7.284 7.260 CDCl3 7.247 7.229 7.210 7.144 7.127
5.627 5.607 5.602 5.584 5.565 5.559 5.540 5.471 5.426 5.401 5.265 5.260 5.256 5.053 5.034 5.028 5.026 5.023
2.964 2.961 2.944 2.942 2.930 2.928 2.925 2.923 2.913 2.912 2.909 2.907 2.892 2.890 2.838 2.819 2.800 2.585 2.566 2.552 2.547 2.533 2.515 1.959
Ph
O
O
3b
2.21 1.13 2.16 1.31 2.12 1.11 2.03 1.32 2.14 1.12 3.04
f1 (ppm)

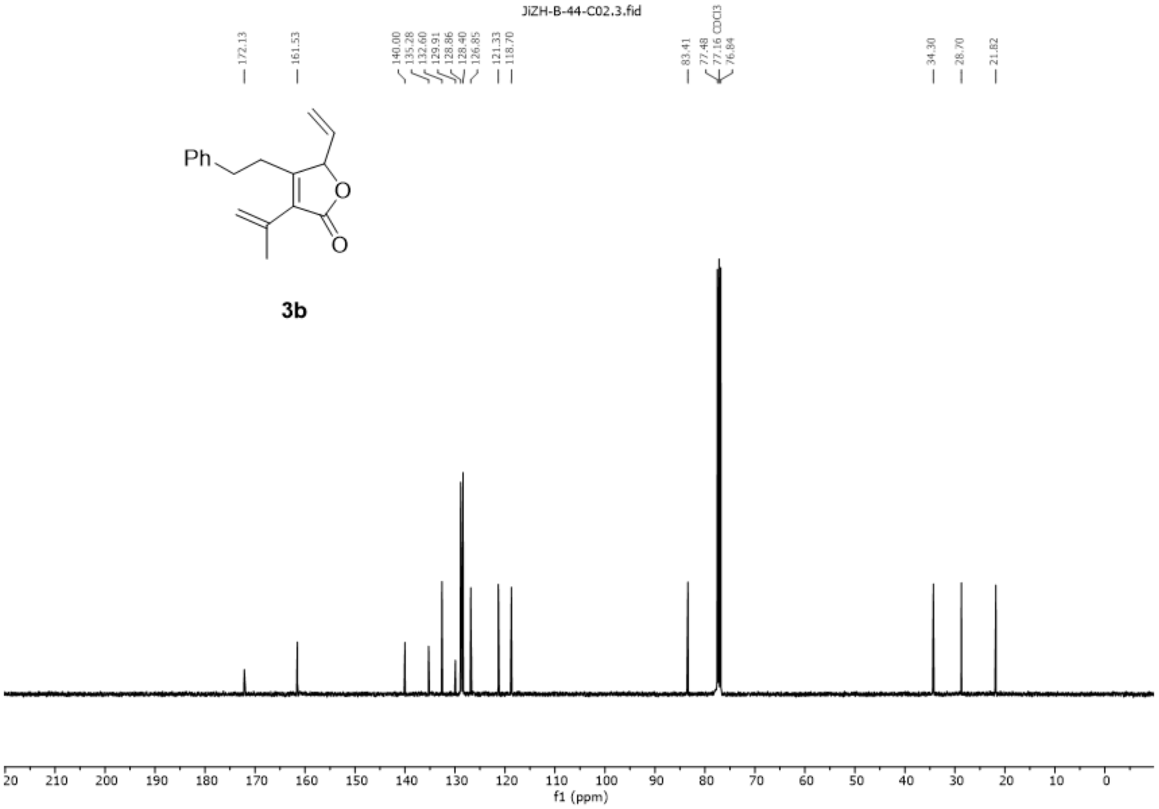
JiZH-B-44-C02.3.fid
172.13
161.53
140.00 135.28 132.60 129.91 128.86 128.40 126.85
121.33 118.70
83.41
77.48 CDCl3 77.16 76.84
34.30
28.70
21.82
Ph
O
O
3b
f1 (ppm)

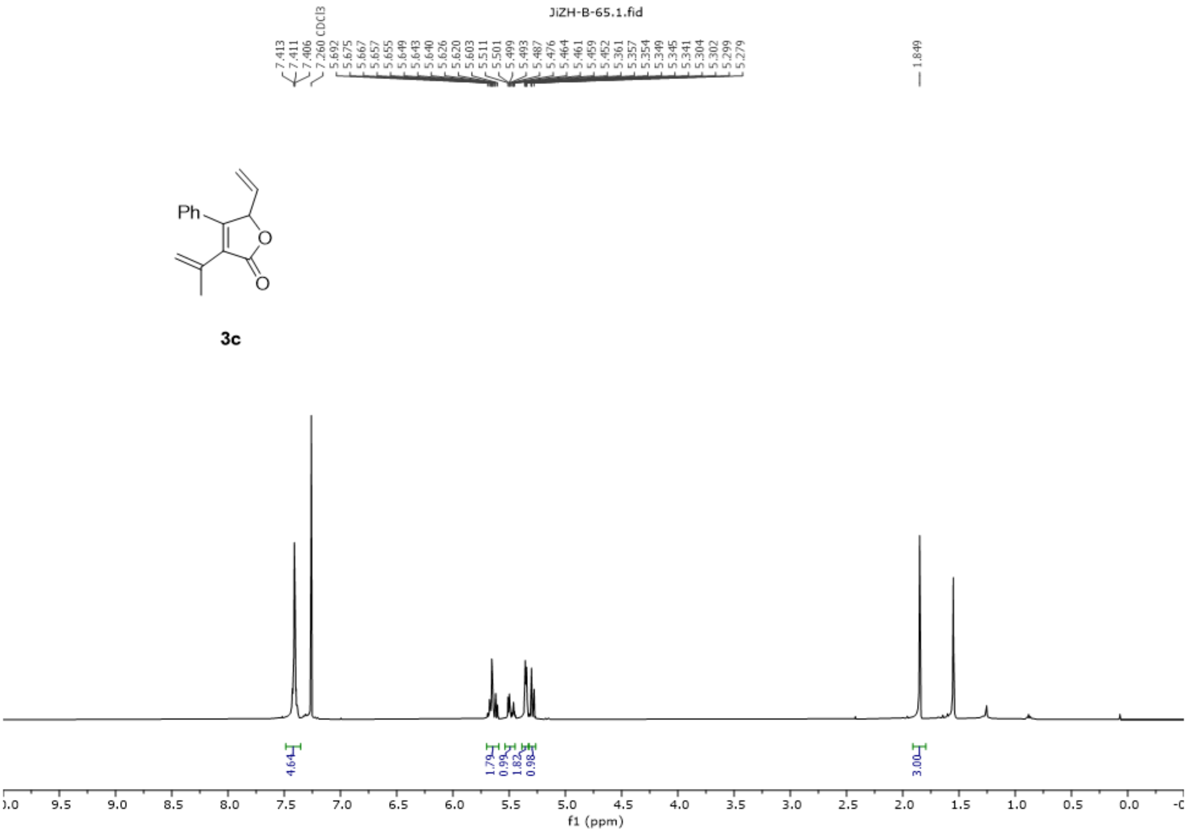

3c

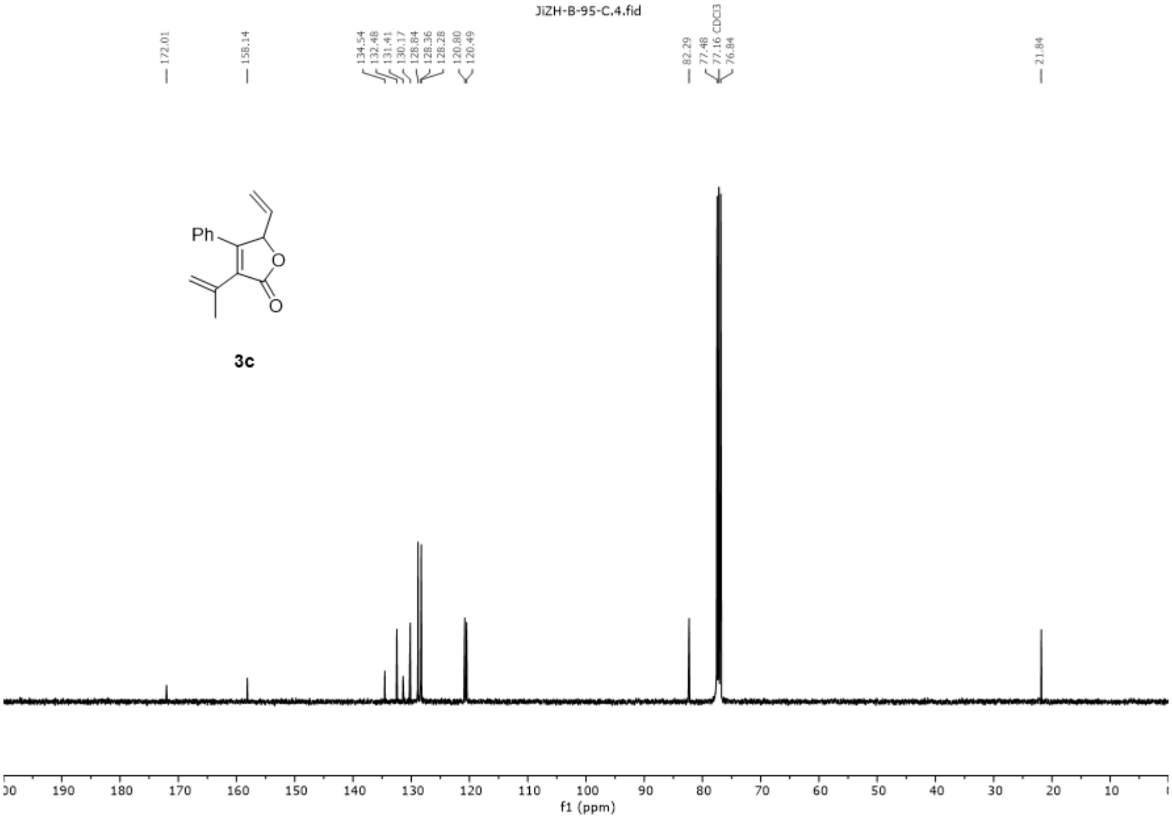

3c

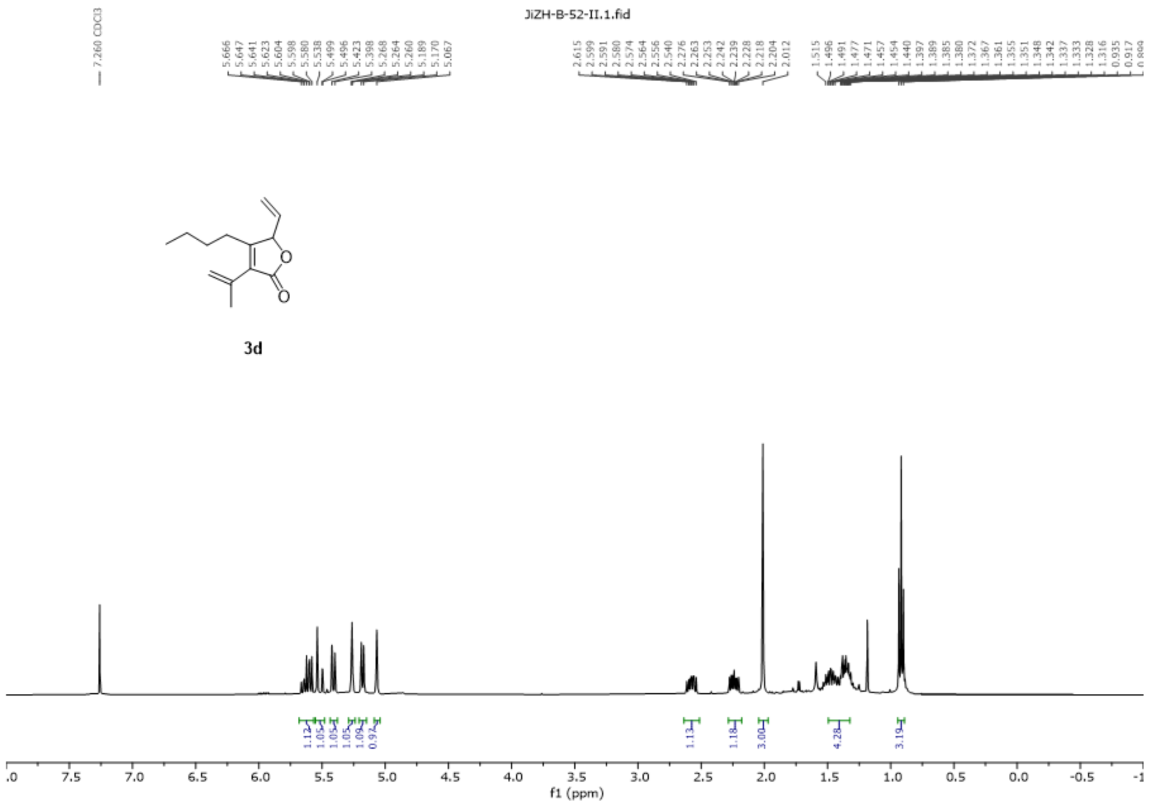
JiZH-B-52-II.1.fid
7.260 CDCl3
5.666
5.647
5.641
5.623
5.604
5.598
5.580
5.538
5.499
5.496
5.423
5.398
5.268
5.264
5.260
5.189
5.170
5.067
2.615
2.599
2.591
2.580
2.574
2.564
2.556
2.540
2.276
2.263
2.253
2.242
2.239
2.228
2.218
2.204
2.012
1.515
1.496
1.491
1.477
1.471
1.457
1.454
1.440
1.397
1.389
1.385
1.380
1.372
1.367
1.361
1.355
1.351
1.348
1.342
1.337
1.333
1.328
1.316
0.935
0.917
3d
1.12
1.05
1.05
1.05
1.09
0.97
1.13
1.18
3.00
4.28
3.19
f1 (ppm)

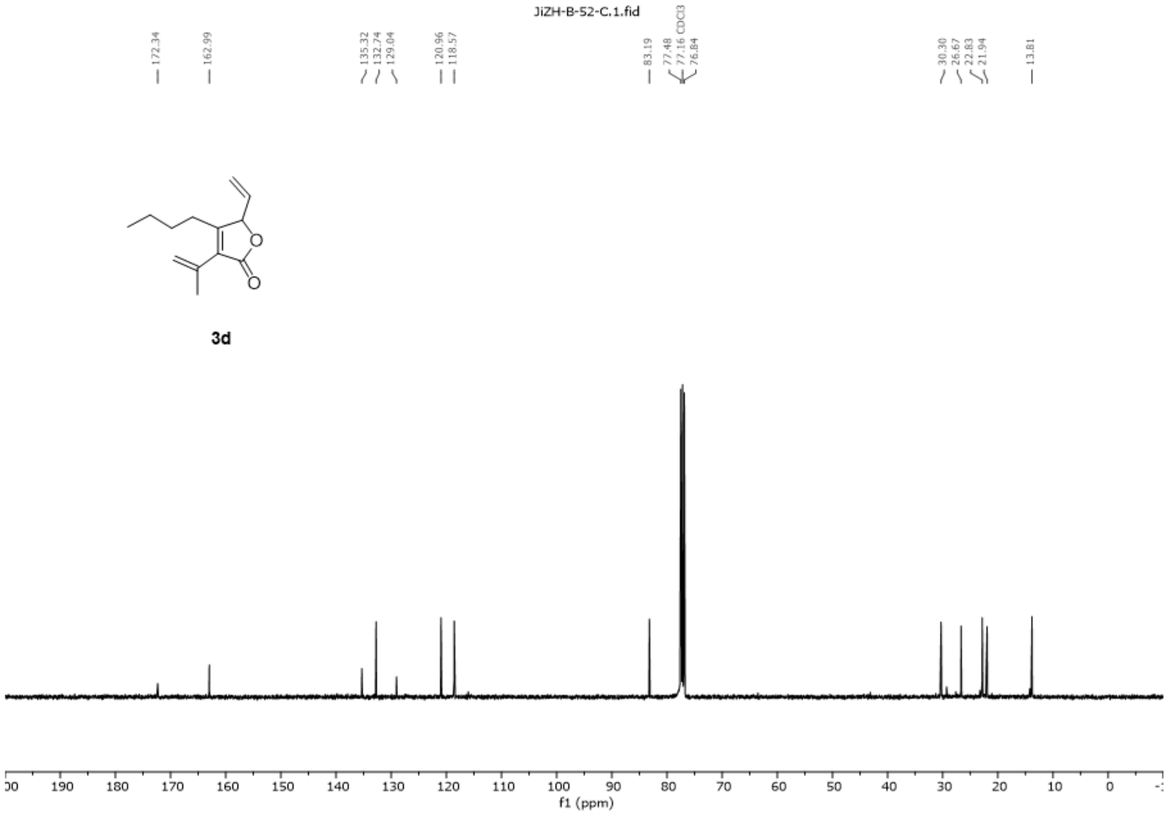
JiZH-B-52-C.1.fid
172.34
162.99
135.32
132.74
129.04
120.96
118.57
83.19
77.48
77.16 CDCl3
76.84
30.30
26.67
22.83
21.94
13.81
3d
f1 (ppm)

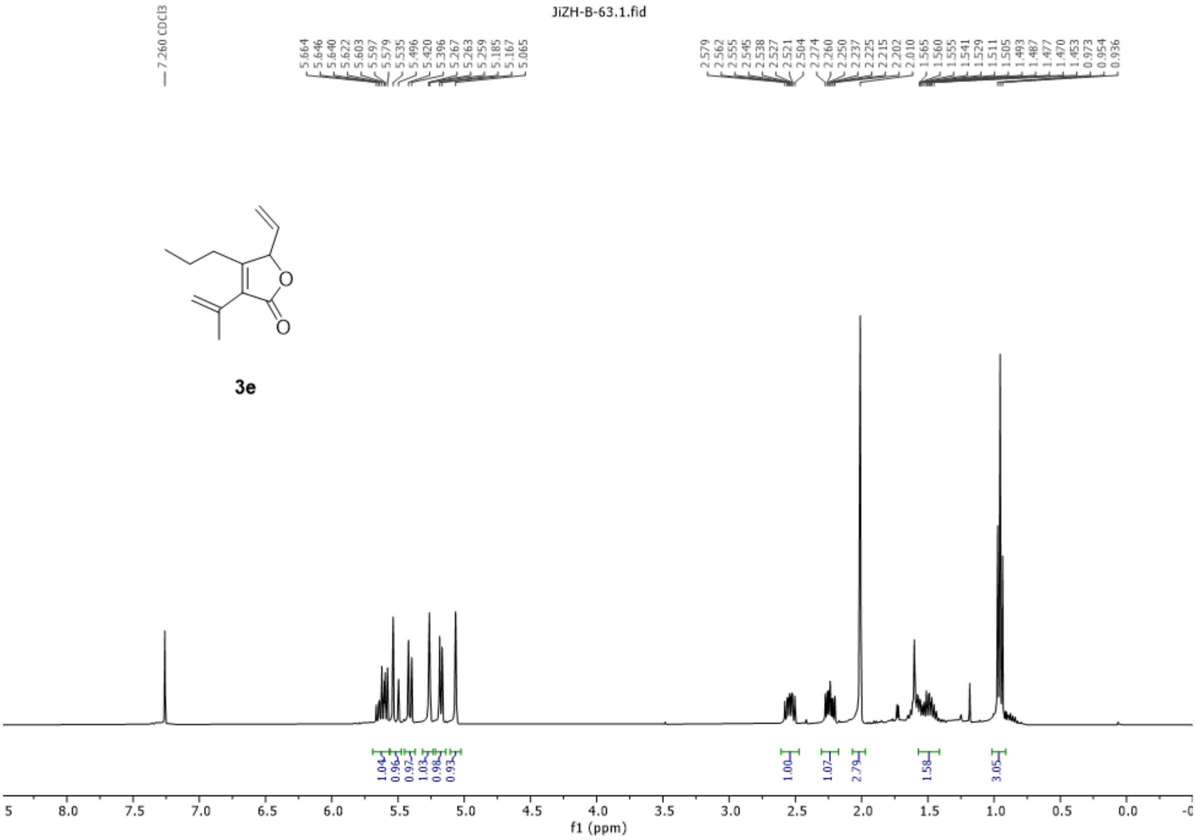

JiZH-B-63.1.fid
7.260 CDCl3
5.664 5.646 5.640 5.622 5.603 5.597 5.579 5.535 5.496 5.420 5.396 5.267 5.263 5.259 5.185 5.167 5.065
2.579 2.562 2.555 2.545 2.538 2.527 2.521 2.504 2.274 2.260 2.250 2.237 2.225 2.215 2.202 2.010 1.565 1.560 1.555 1.541 1.529 1.511 1.505 1.493 1.487 1.477 1.470 1.453 0.973 0.954 0.936
O
3e
1.04 0.96 0.97 1.03 0.98 0.93 1.00 1.07 2.79 1.58 3.05
f1 (ppm)

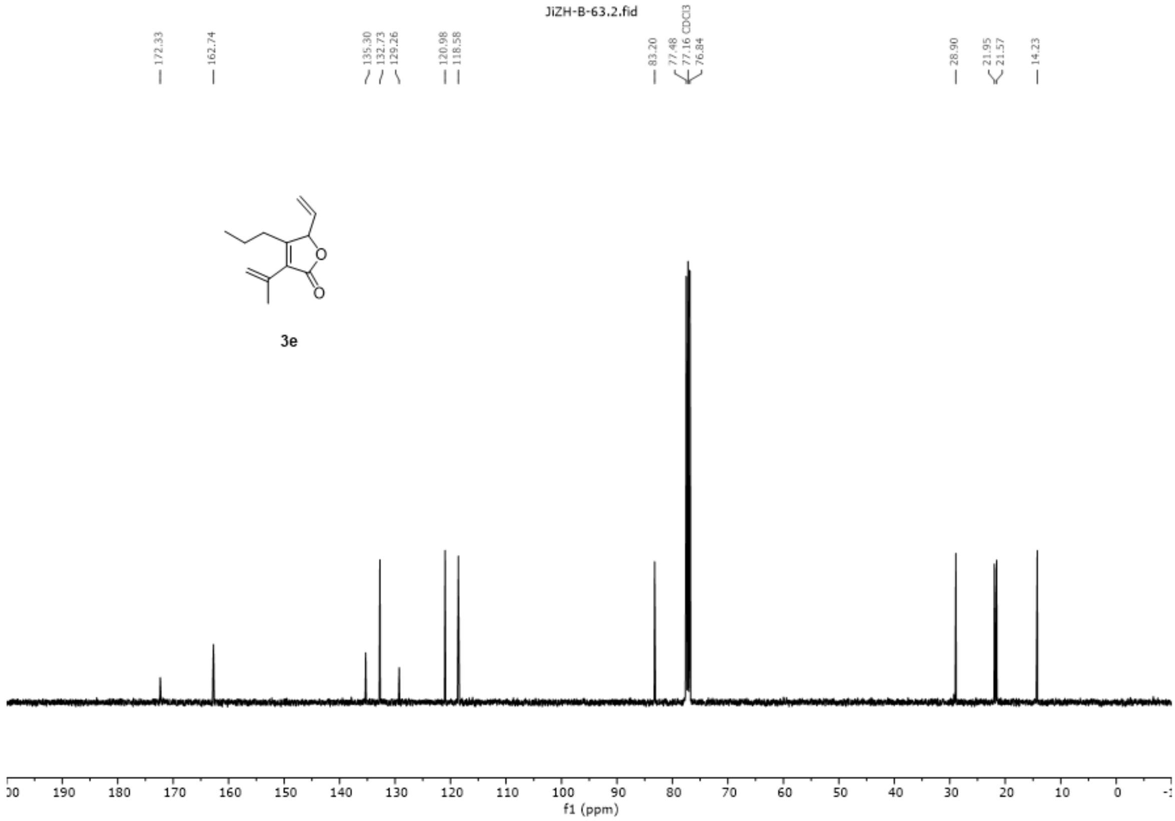

JiZH-B-63.2.fid
172.33
162.74
135.30 132.73 129.26
120.98 118.58
83.20
77.48 77.16 CDCl3 76.84
28.90
21.95 21.57
14.23
O
3e
f1 (ppm)

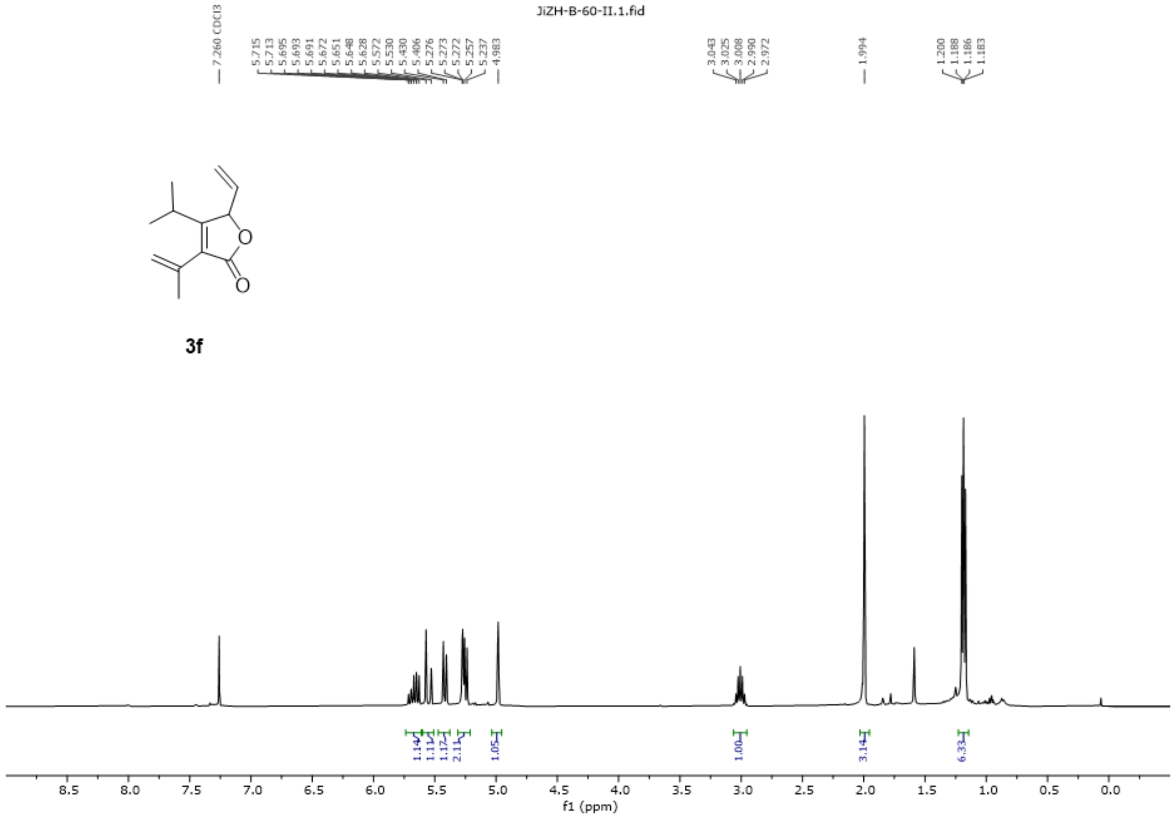
JiZH-B-60-II.1.fid
7.260 CDCl3
5.715
5.713
5.695
5.693
5.691
5.672
5.651
5.648
5.628
5.572
5.530
5.430
5.406
5.276
5.273
5.272
5.257
5.237
4.983
3.043
3.025
3.008
2.990
2.972
1.994
1.200
1.188
1.186
1.183
O
O
3f
1.14
1.11
1.12
2.11
1.05
1.00
3.14
6.33
8.5 8.0 7.5 7.0 6.5 6.0 5.5 5.0 4.5 4.0 3.5 3.0 2.5 2.0 1.5 1.0 0.5 0.0
f1 (ppm)

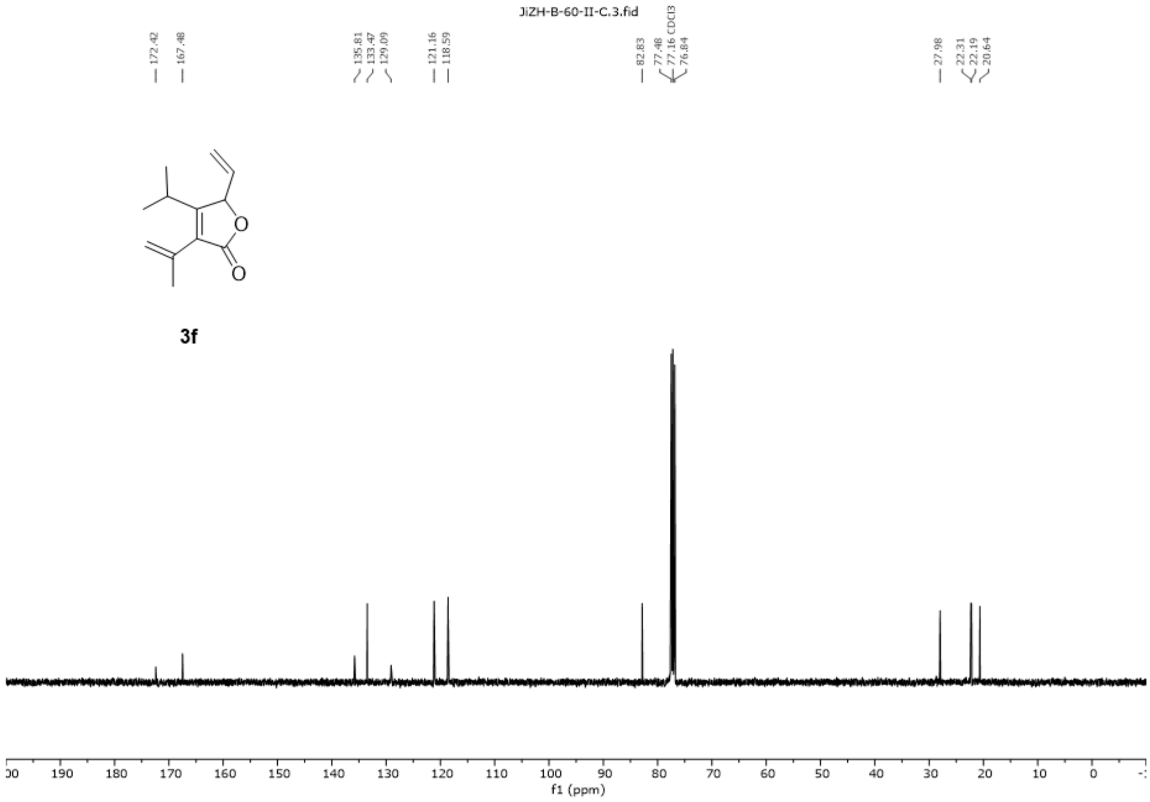
JiZH-B-60-II-C.3.fid
172.42
167.48
135.81
133.47
129.09
121.16
118.59
82.83
77.48
77.16 CDCl3
76.84
27.98
22.31
22.19
20.64
O
O
3f
190 180 170 160 150 140 130 120 110 100 90 80 70 60 50 40 30 20 10 0
f1 (ppm)

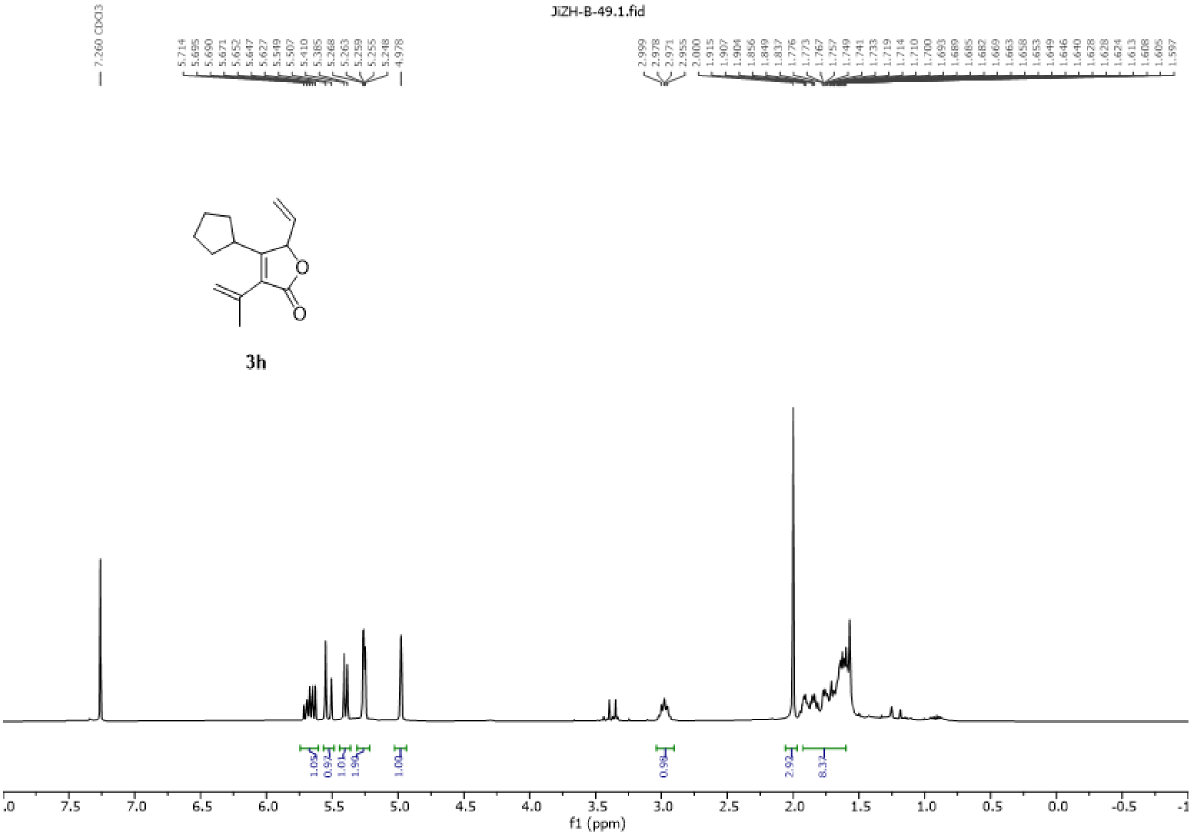
JiZH-B-49.1.fid
7.260 CDCl3
5.714 5.695 5.690 5.671 5.652 5.647 5.627 5.549 5.507 5.410 5.385 5.268 5.263 5.259 5.255 5.248 4.978
2.999 2.978 2.971 2.955 2.000 1.915 1.907 1.904 1.856 1.849 1.837 1.776 1.773 1.767 1.757 1.749 1.741 1.733 1.719 1.714 1.710 1.700 1.693 1.689 1.685 1.682 1.669 1.663 1.658 1.653 1.649 1.646 1.640 1.628 1.624 1.613 1.608 1.605 1.597
3h
1.05 0.97 1.01 1.90 1.00 0.98 2.92 8.37
f1 (ppm)

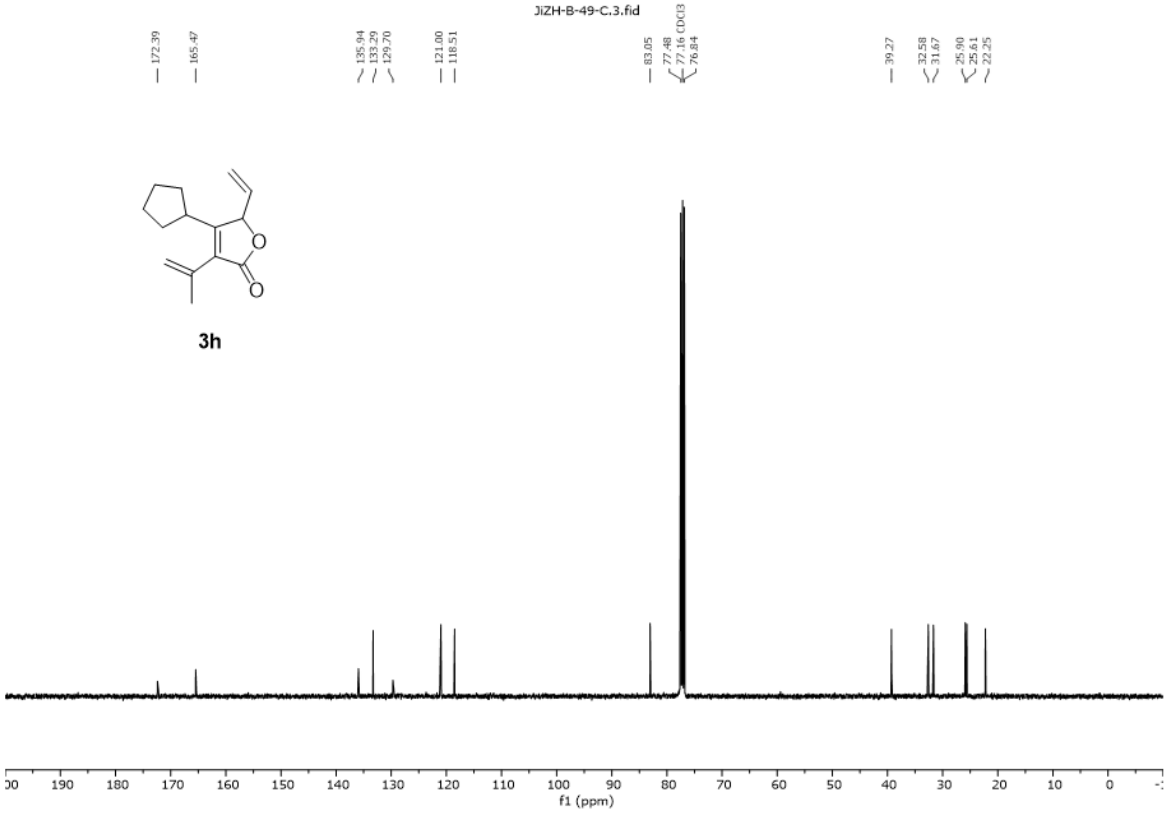
JiZH-B-49-C.3.fid
172.39 165.47 135.94 133.29 129.70 121.00 118.51 83.05 77.48 77.16 CDCl3 76.84 39.27 32.58 31.67 25.90 25.61 22.25
3h
f1 (ppm)

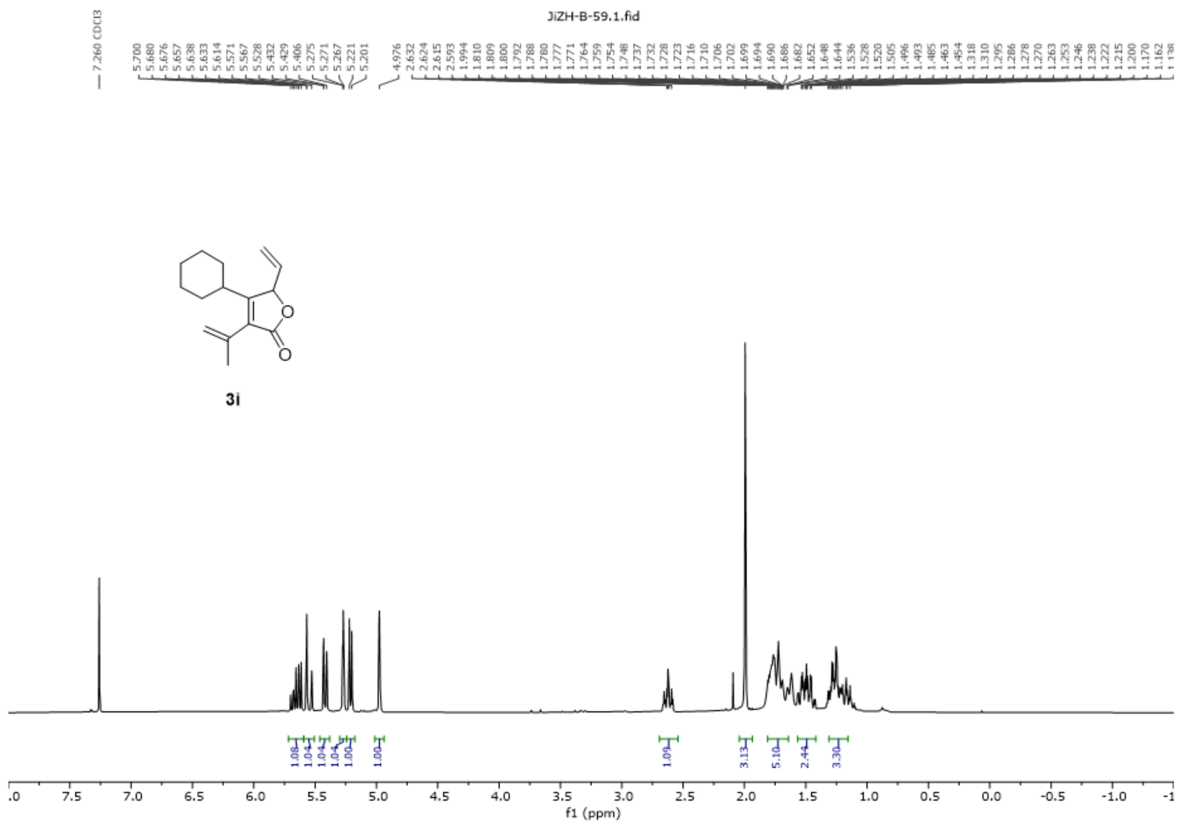
JiZH-B-59.1.fid
7.260 CDCl3
5.700
5.680
5.676
5.657
5.638
5.633
5.614
5.571
5.567
5.528
5.432
5.429
5.406
5.275
5.271
5.267
5.221
5.201
4.976
2.632
2.624
2.615
2.593
1.994
1.810
1.809
1.800
1.792
1.788
1.780
1.777
1.771
1.764
1.759
1.754
1.748
1.737
1.732
1.728
1.723
1.716
1.710
1.706
1.702
1.699
1.694
1.690
1.686
1.682
1.652
1.648
1.644
1.536
1.528
1.520
1.505
1.496
1.493
1.485
1.463
1.454
1.318
1.310
1.295
1.286
1.278
1.270
1.263
1.253
1.246
1.238
1.222
1.215
1.200
1.170
1.162
3i
1.08
1.04
1.04
1.04
1.00
1.00
1.09
3.13
5.10
2.44
3.30
f1 (ppm)

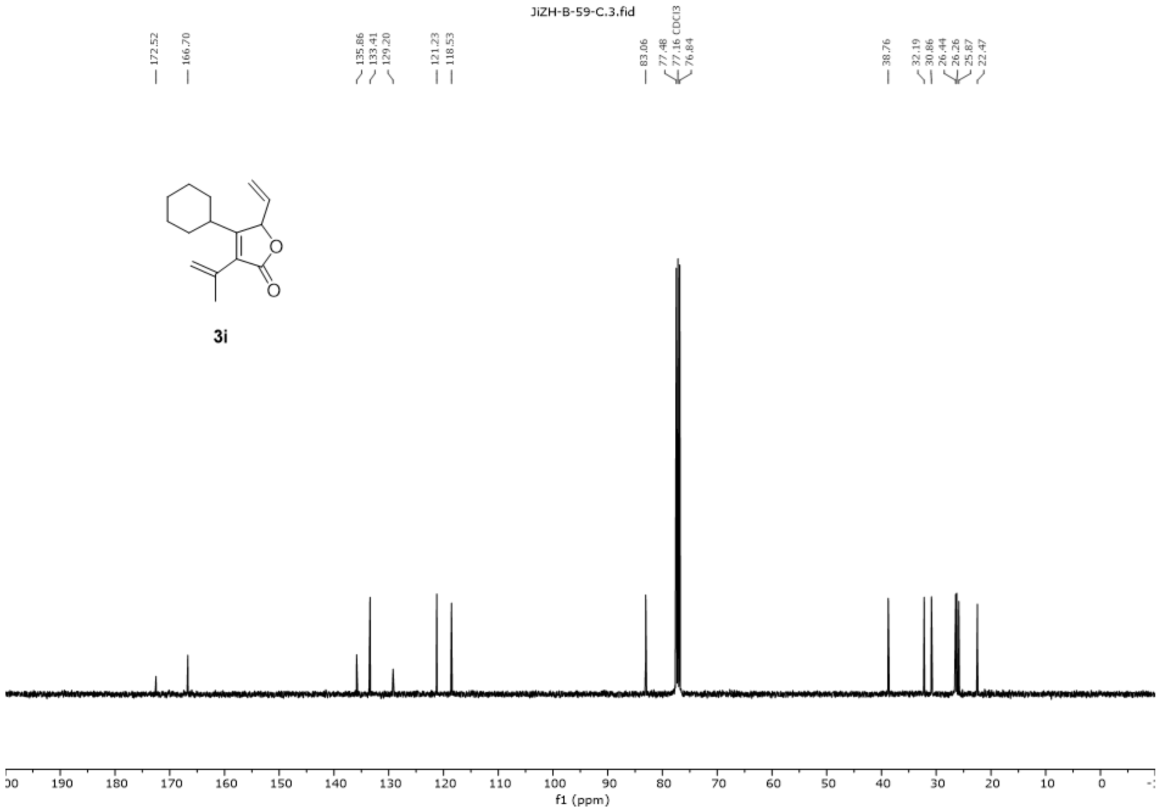
JiZH-B-59-C.3.fid
172.52
166.70
135.86
133.41
129.20
121.23
118.53
83.06
77.48
77.16 CDCl3
76.84
38.76
32.19
30.86
26.44
26.26
25.87
22.47
3i
f1 (ppm)

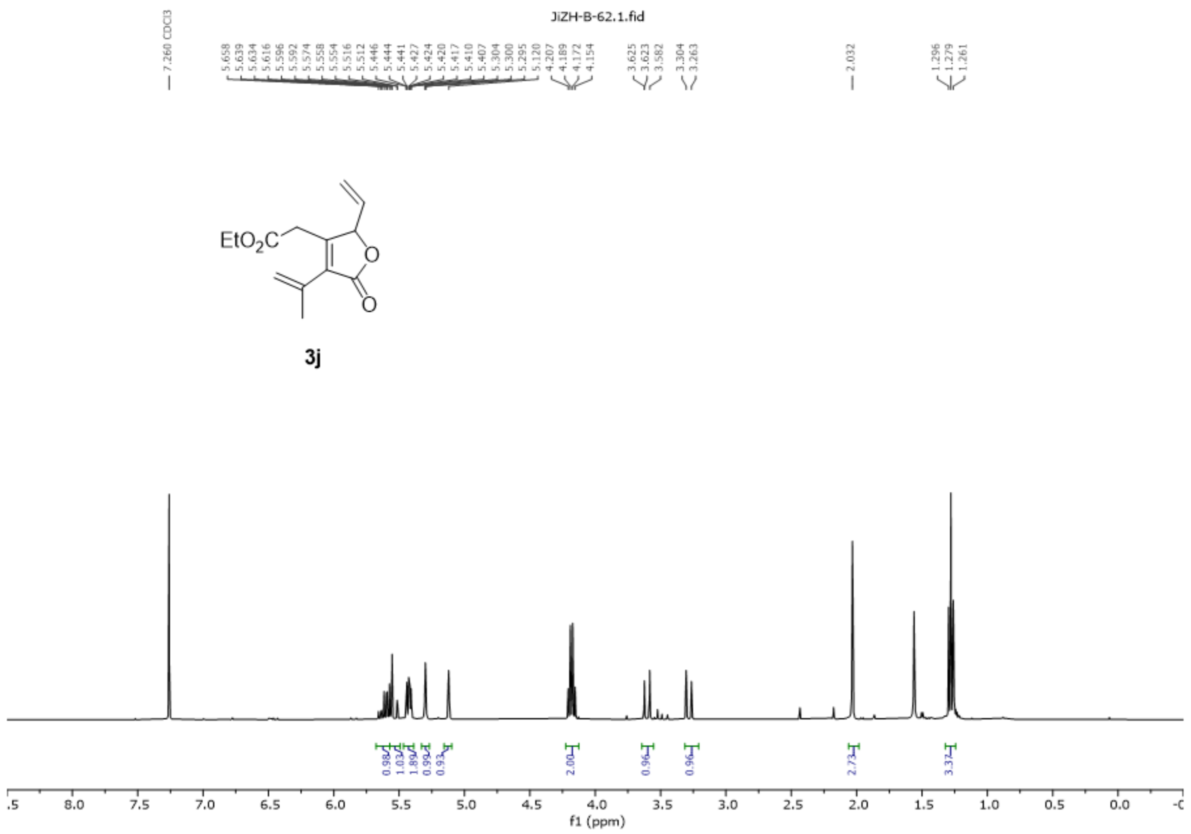

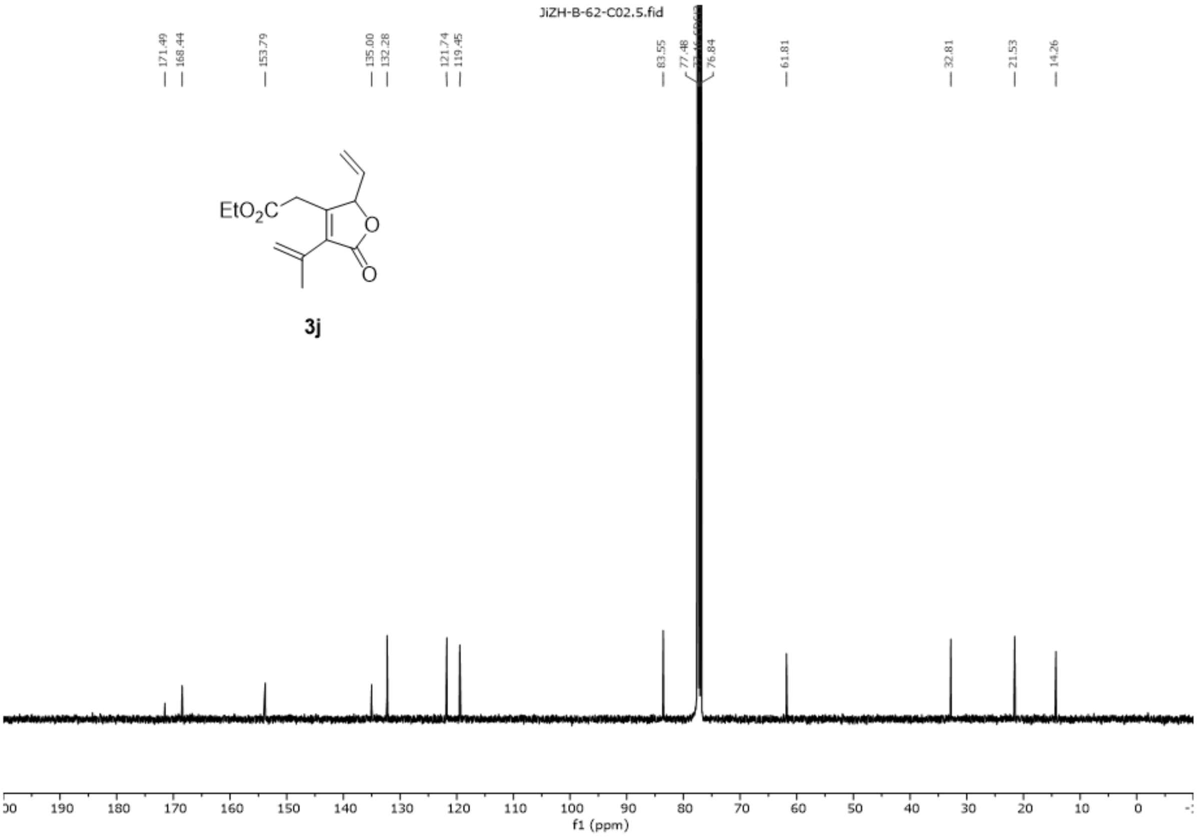

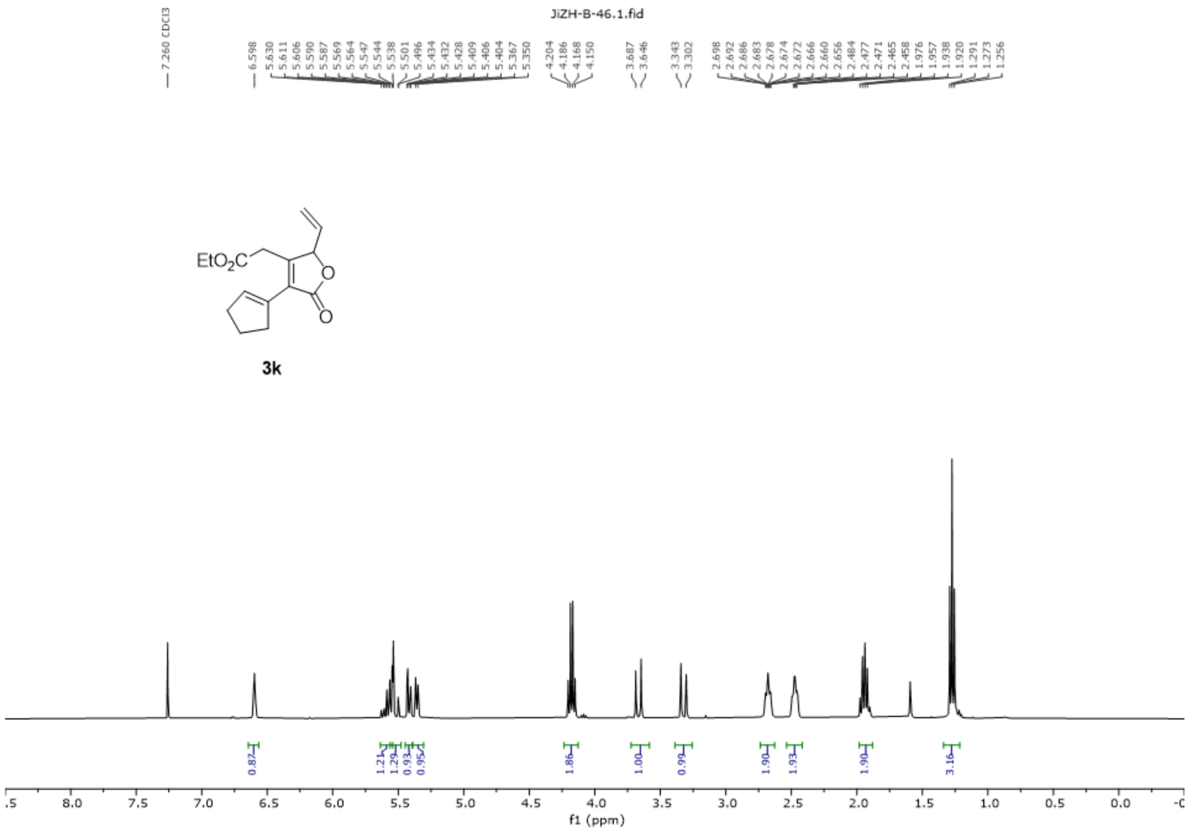
JiZH-B-46.1.fid
7.260 CDCl3
6.598
5.630 5.611 5.606 5.590 5.587 5.569 5.564 5.547 5.544 5.538 5.501 5.496 5.434 5.432 5.428 5.409 5.406 5.404 5.367 5.350
4.204 4.186 4.168 4.150
3.687 3.646
3.343 3.302
2.698 2.692 2.686 2.683 2.678 2.674 2.672 2.666 2.660 2.656 2.484 2.477 2.471 2.465 2.458 1.976 1.957 1.938 1.920 1.291 1.273 1.256
EtO2C
O
3k
0.87
1.21
1.29
0.93
0.95
1.86
1.00
0.99
1.90
1.93
1.90
3.16
f1 (ppm)

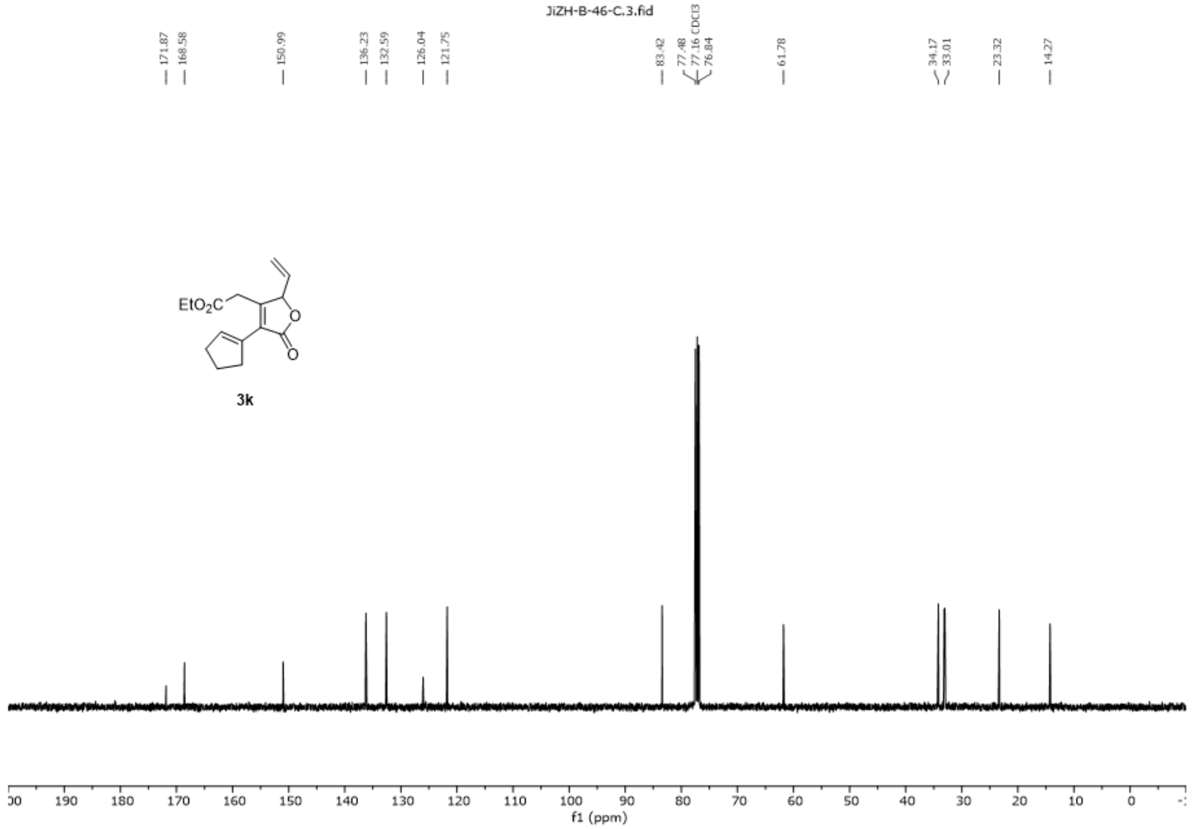
JiZH-B-46-C.3.fid
171.87
168.58
150.99
136.23
132.59
126.04
121.75
83.42
77.48
77.16 CDCl3
76.84
61.78
34.17
33.01
23.32
14.27
EtO2C
O
3k
f1 (ppm)

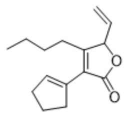

3l

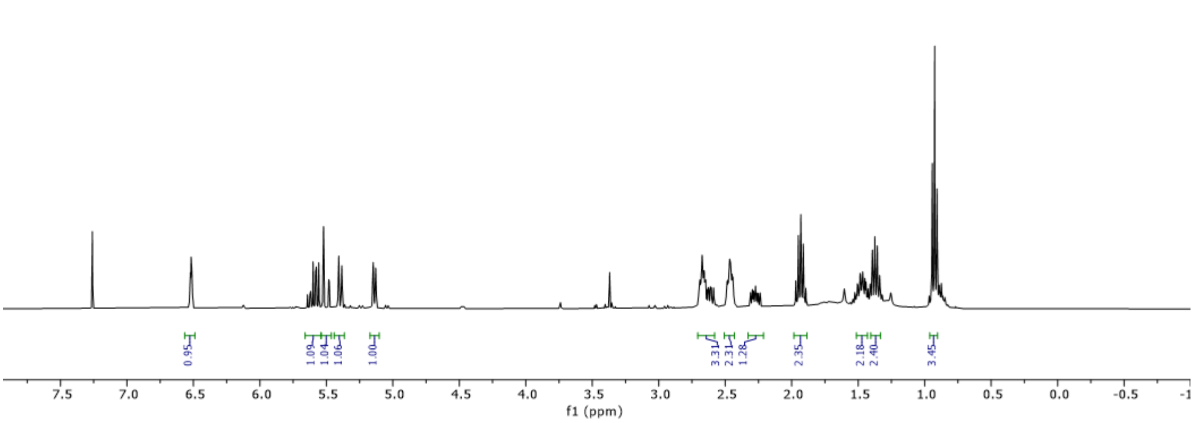

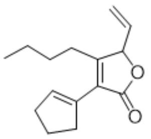

3l

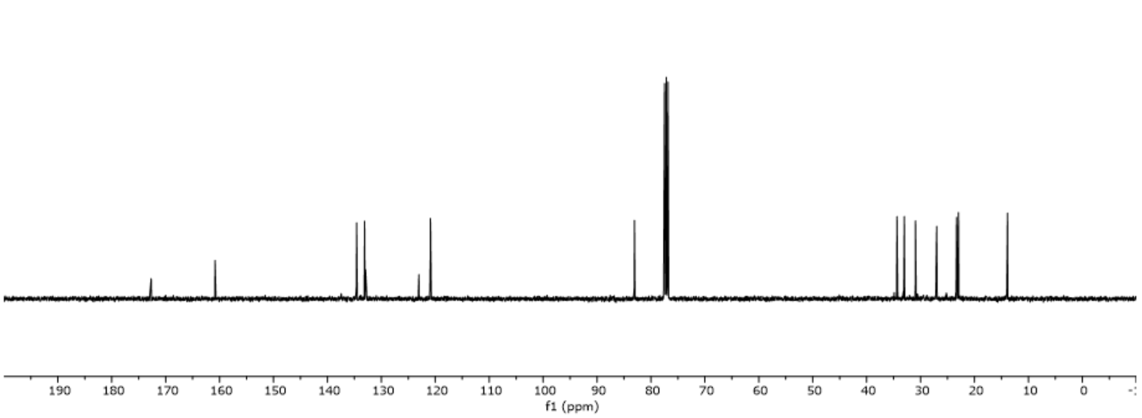

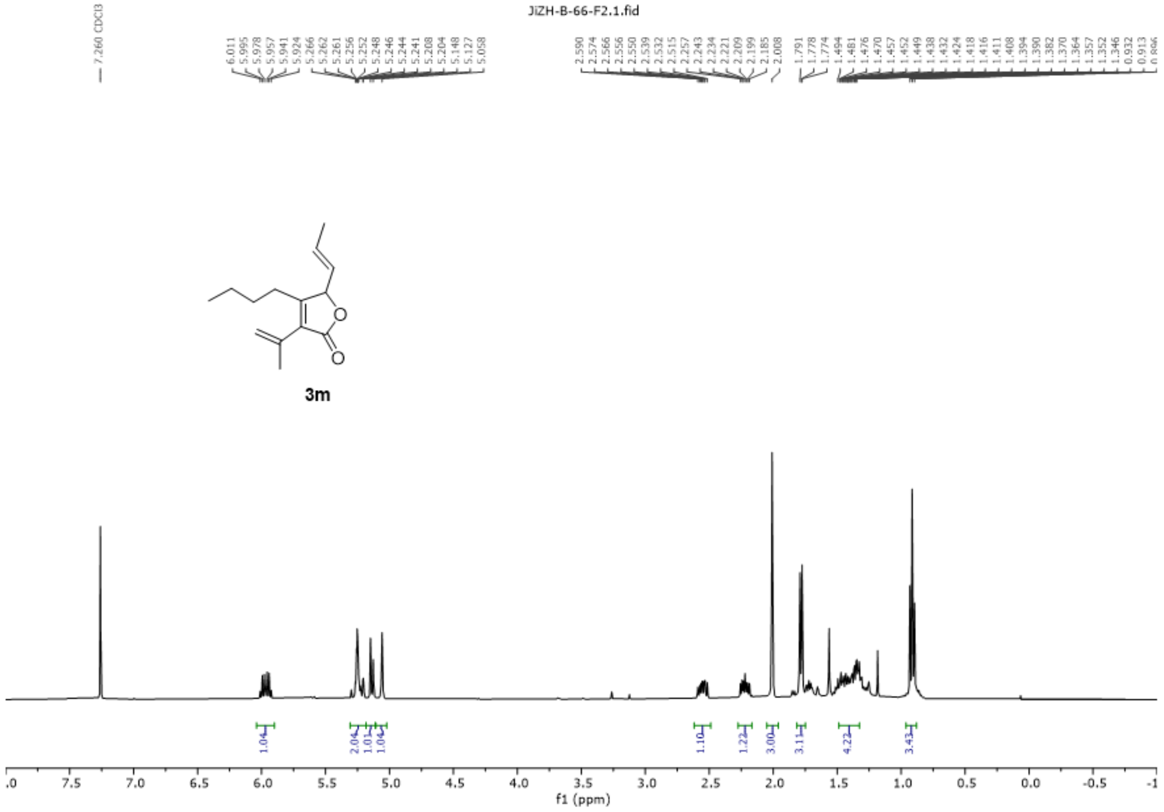
JiZH-B-66-F2.1.fid
3m
f1 (ppm)

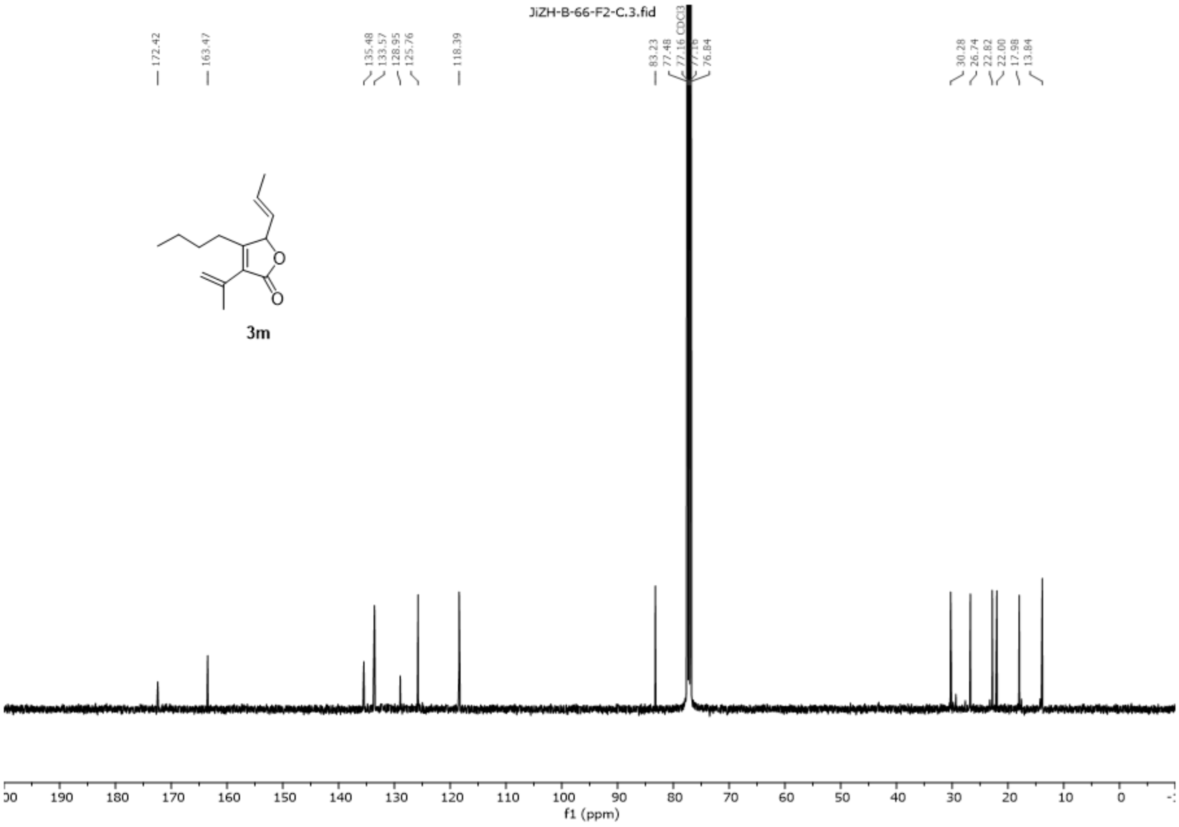
JiZH-B-66-F2-C.3.fid
3m
f1 (ppm)

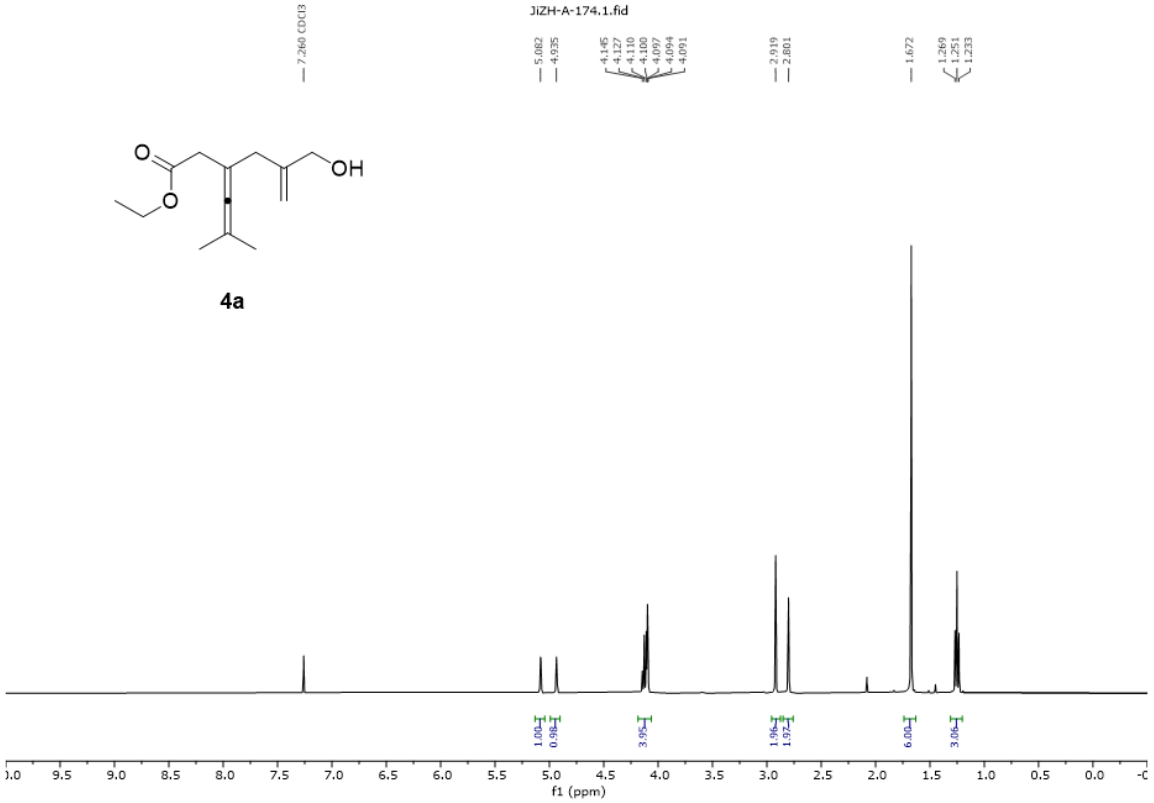

JiZH-A-174.1.fid
7.260 CDCl3
5.082
4.935
4.145
4.127
4.110
4.100
4.097
4.094
4.091
2.919
2.801
1.672
1.269
1.251
1.233
OH
O
O
4a
1.00
0.98
3.95
1.96
1.97
6.00
3.06
f1 (ppm)

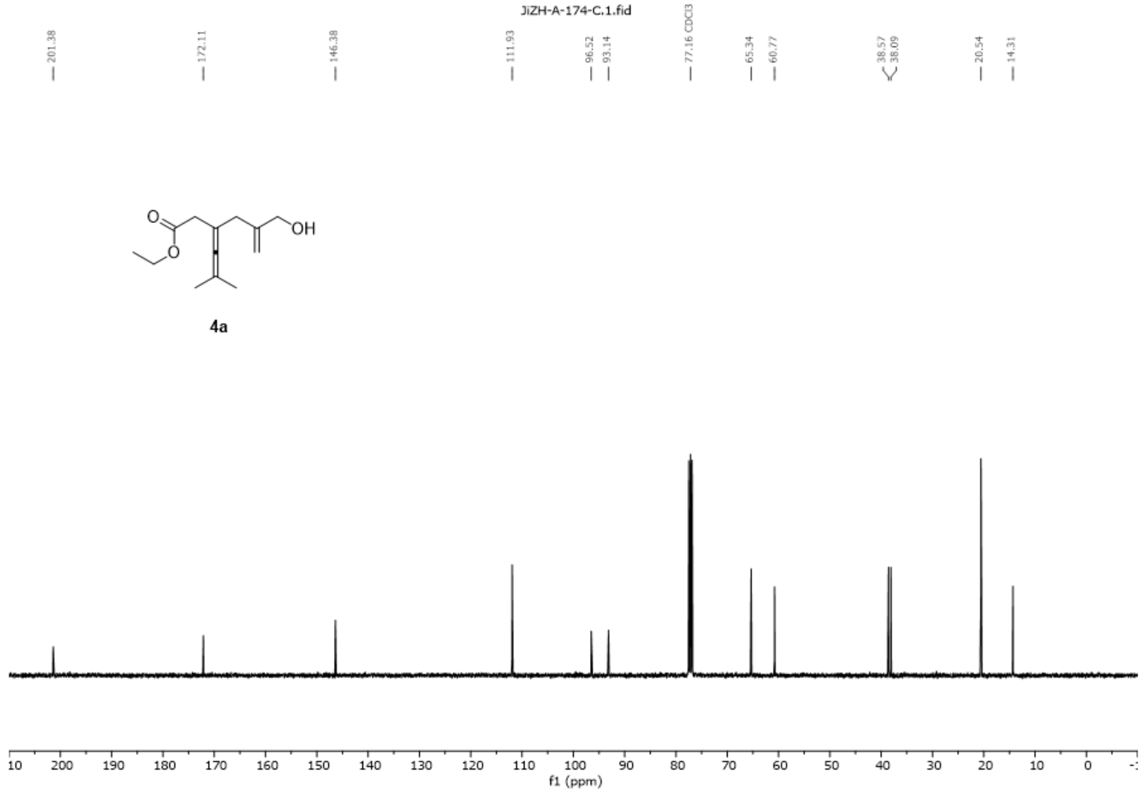

JiZH-A-174-C.1.fid
201.38
172.11
146.38
111.93
96.52
93.14
77.16 CDCl3
65.34
60.77
38.57
38.09
20.54
14.31
OH
O
O
4a
f1 (ppm)

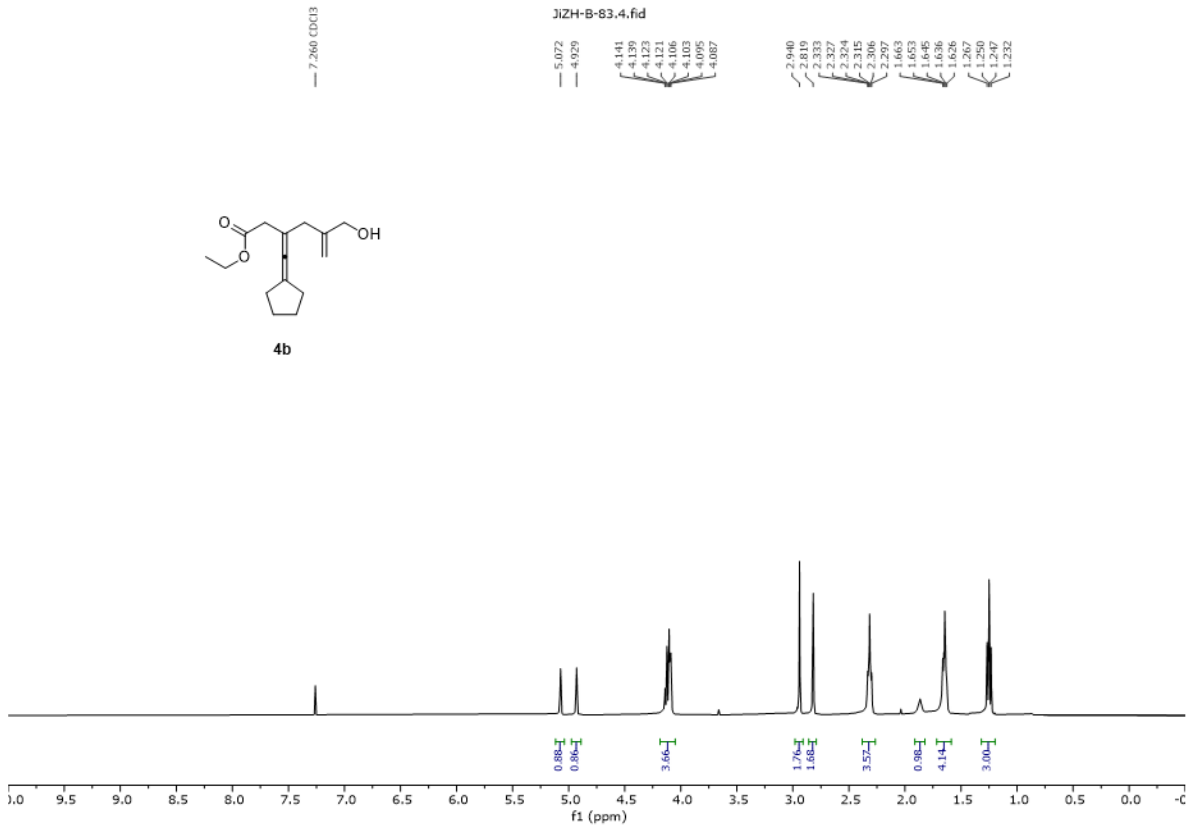
JiZH-B-83.4.fid
7.260 CDCl3
5.072
4.929
4.141
4.139
4.123
4.121
4.106
4.103
4.095
4.087
2.940
2.819
2.333
2.327
2.324
2.315
2.306
2.297
1.663
1.653
1.645
1.636
1.626
1.267
1.250
1.247
1.232
OH
O
O
4b
0.88
0.86
3.66
1.76
1.68
3.57
0.98
4.14
3.00
f1 (ppm)

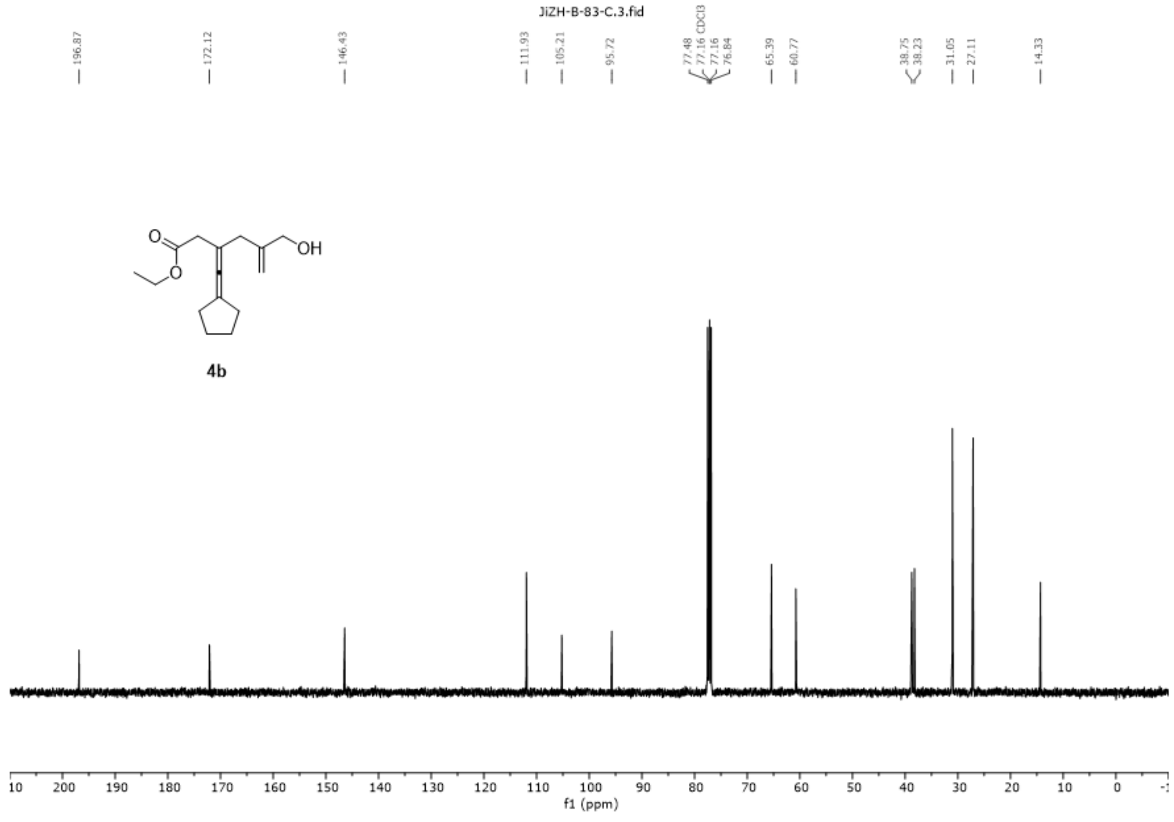
JiZH-B-83-C.3.fid
196.87
172.12
146.43
111.93
105.21
95.72
77.48
77.16 CDCl3
77.16
76.84
65.39
60.77
38.75
38.23
31.05
27.11
14.33
OH
O
O
4b
f1 (ppm)

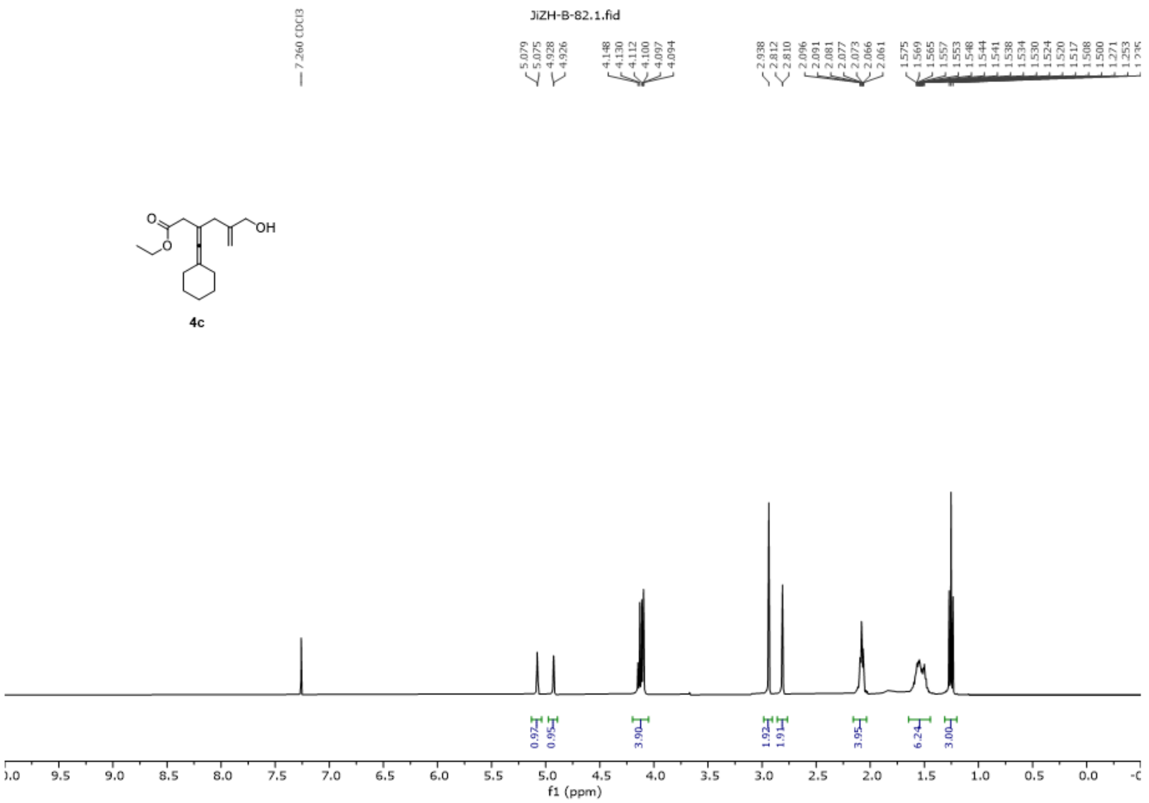
JiZH-B-82.1.fid
7.260 CDCl3
5.079
5.075
4.928
4.926
4.148
4.130
4.112
4.100
4.097
4.094
2.938
2.812
2.810
2.096
2.091
2.081
2.077
2.073
2.066
2.061
1.575
1.569
1.565
1.557
1.553
1.548
1.544
1.541
1.538
1.534
1.530
1.524
1.520
1.517
1.508
1.500
1.271
1.253
OH
4c
0.97
0.95
3.90
1.92
1.91
3.95
6.24
3.00
f1 (ppm)

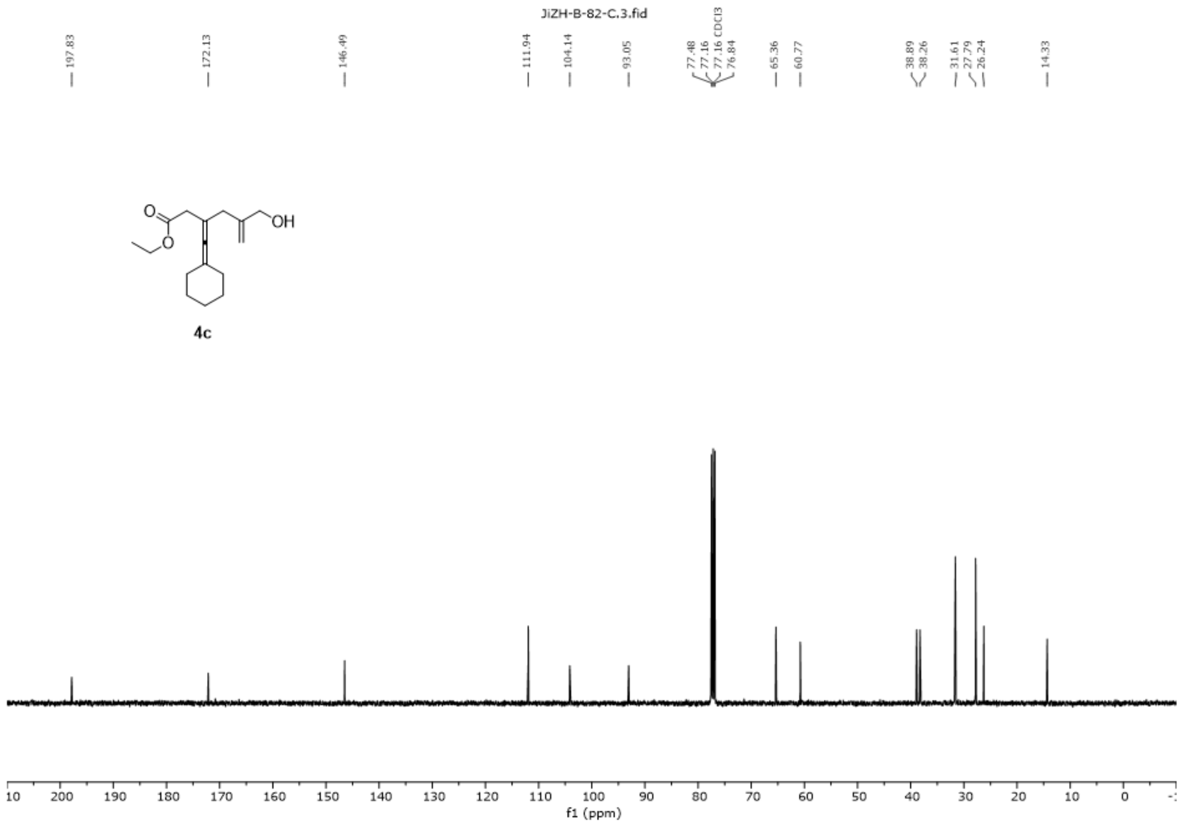
JiZH-B-82-C.3.fid
197.83
172.13
146.49
111.94
104.14
93.05
77.48
77.16
77.16 CDCl3
76.84
65.36
60.77
38.89
38.26
31.61
27.79
26.24
14.33
OH
4c
f1 (ppm)

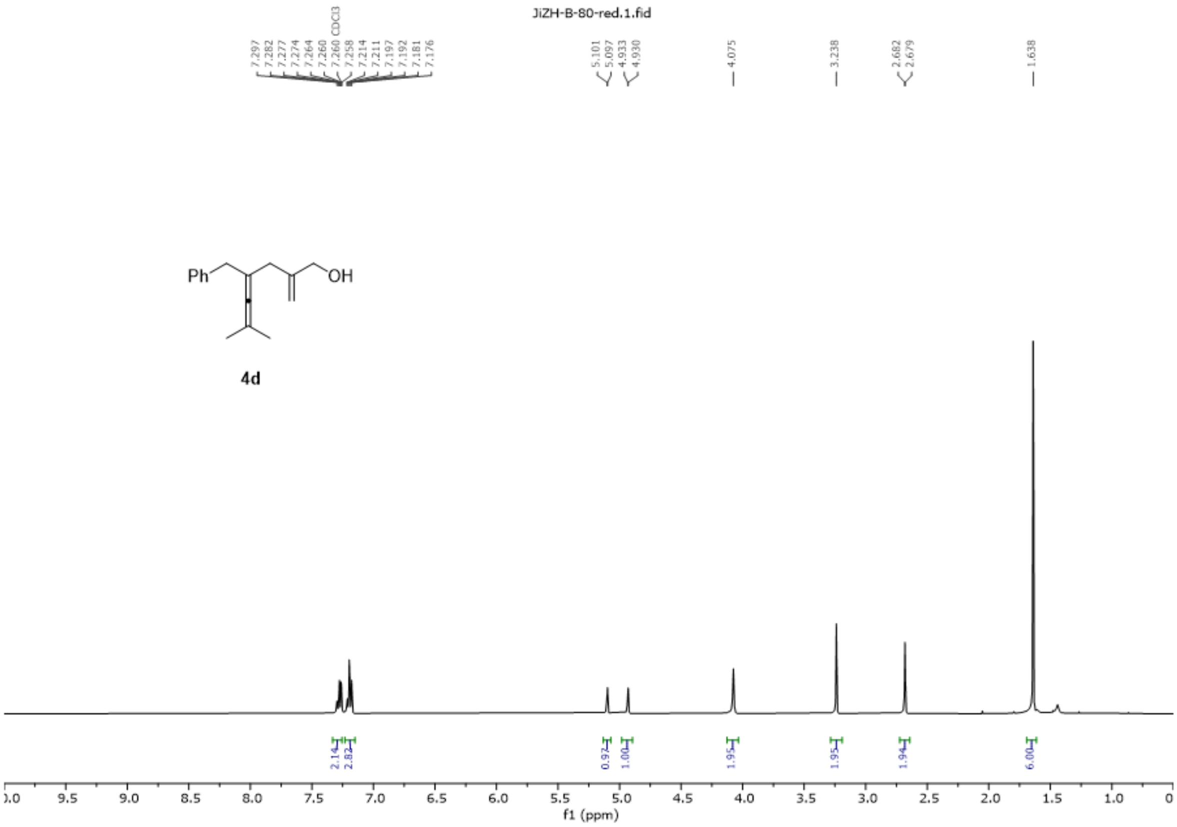
JiZH-B-80-red.1.fid
7.297 7.282 7.277 7.274 7.264 7.260 7.260 CDCl3 7.258 7.214 7.211 7.197 7.192 7.181 7.176
5.101 5.097 4.933 4.930
4.075
3.238
2.682 2.679
1.638
Ph
OH
4d
2.14 2.82 0.97 1.00 1.95 1.95 1.94 6.00
f1 (ppm)

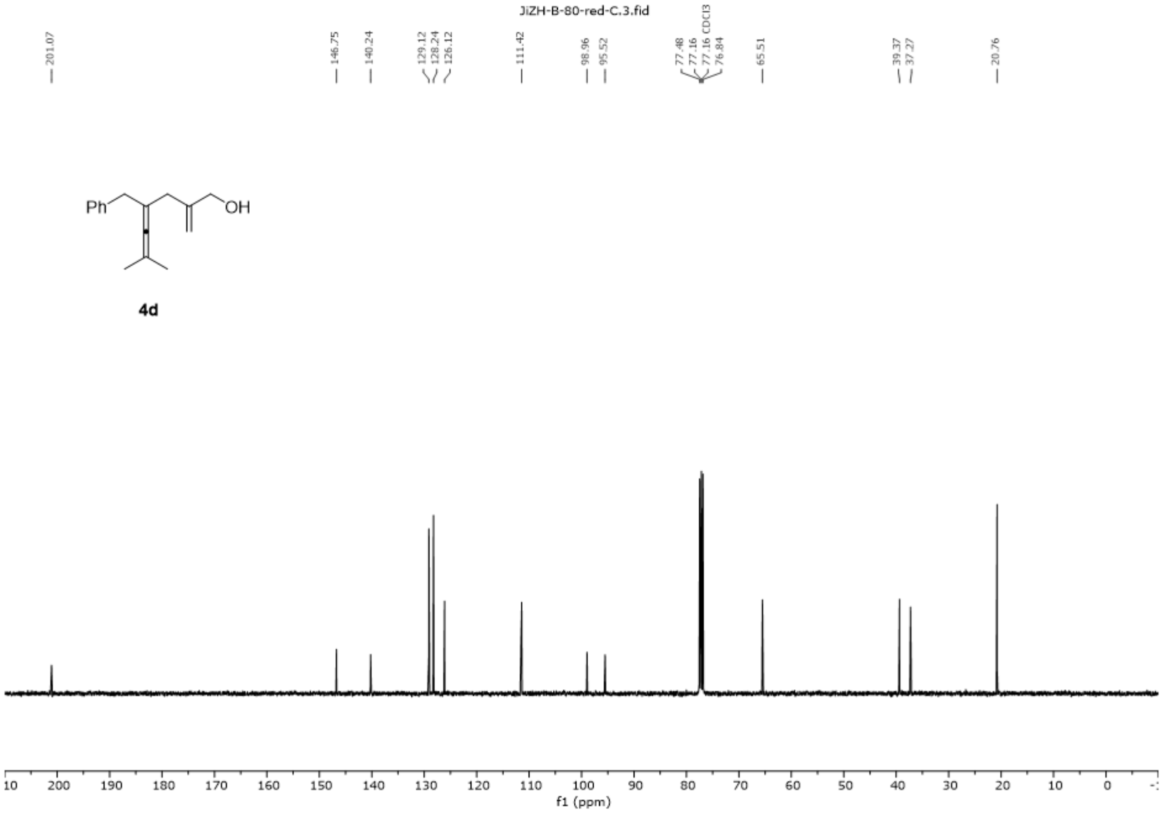
JiZH-B-80-red-C.3.fid
201.07
146.75 140.24 129.12 128.24 126.12 111.42 98.96 95.52
77.48 77.16 77.16 CDCl3 76.84
65.51
39.37 37.27
20.76
Ph
OH
4d
f1 (ppm)

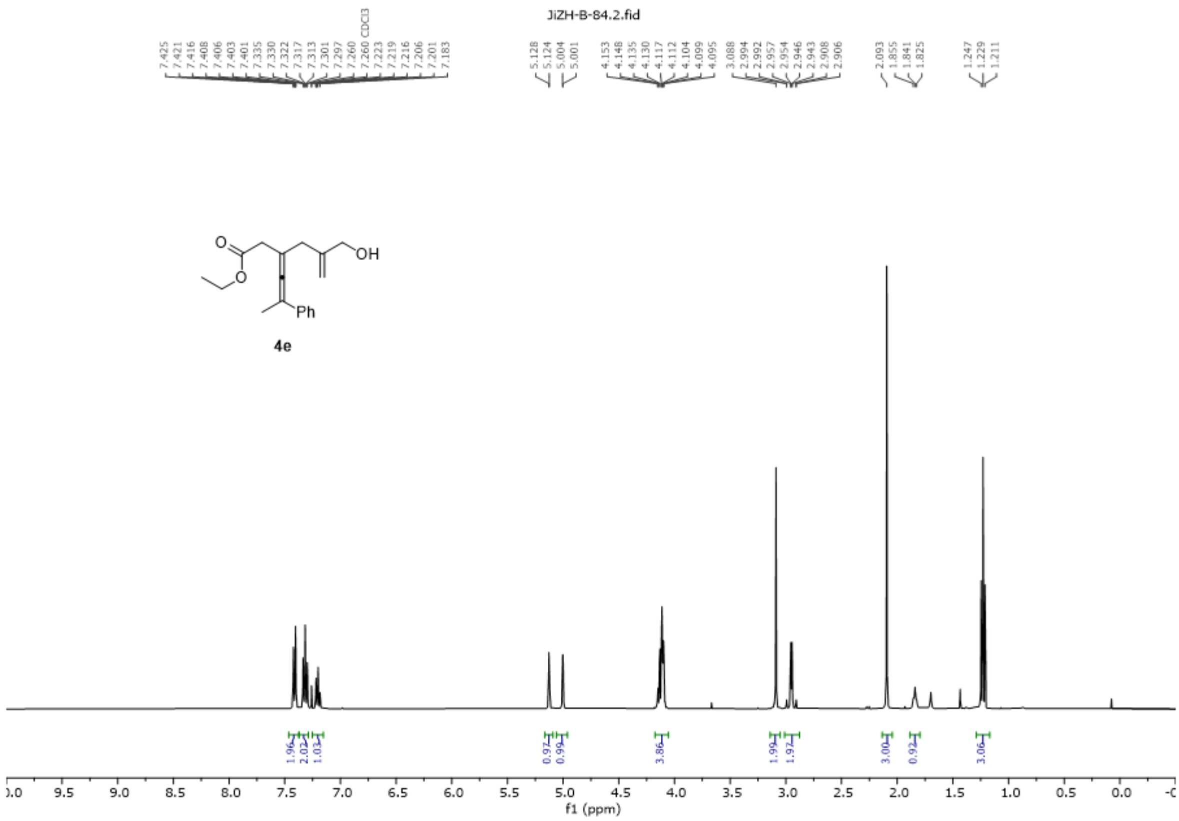
JiZH-B-84.2.fid
7.425 7.421 7.416 7.408 7.406 7.403 7.401 7.335 7.330 7.322 7.317 7.313 7.301 7.297 7.260 7.260 CDCl3 7.223 7.219 7.216 7.206 7.201 7.183
5.128 5.124 5.004 5.001
4.153 4.148 4.135 4.130 4.117 4.112 4.104 4.099 4.095
3.088 2.994 2.992 2.957 2.954 2.946 2.943 2.908 2.906
2.093 1.855 1.841 1.825
1.247 1.229 1.211
OH
O
O
Ph
4e
1.96 2.02 1.03 0.97 0.99 3.86 1.99 1.97 3.00 0.92 3.06
f1 (ppm)

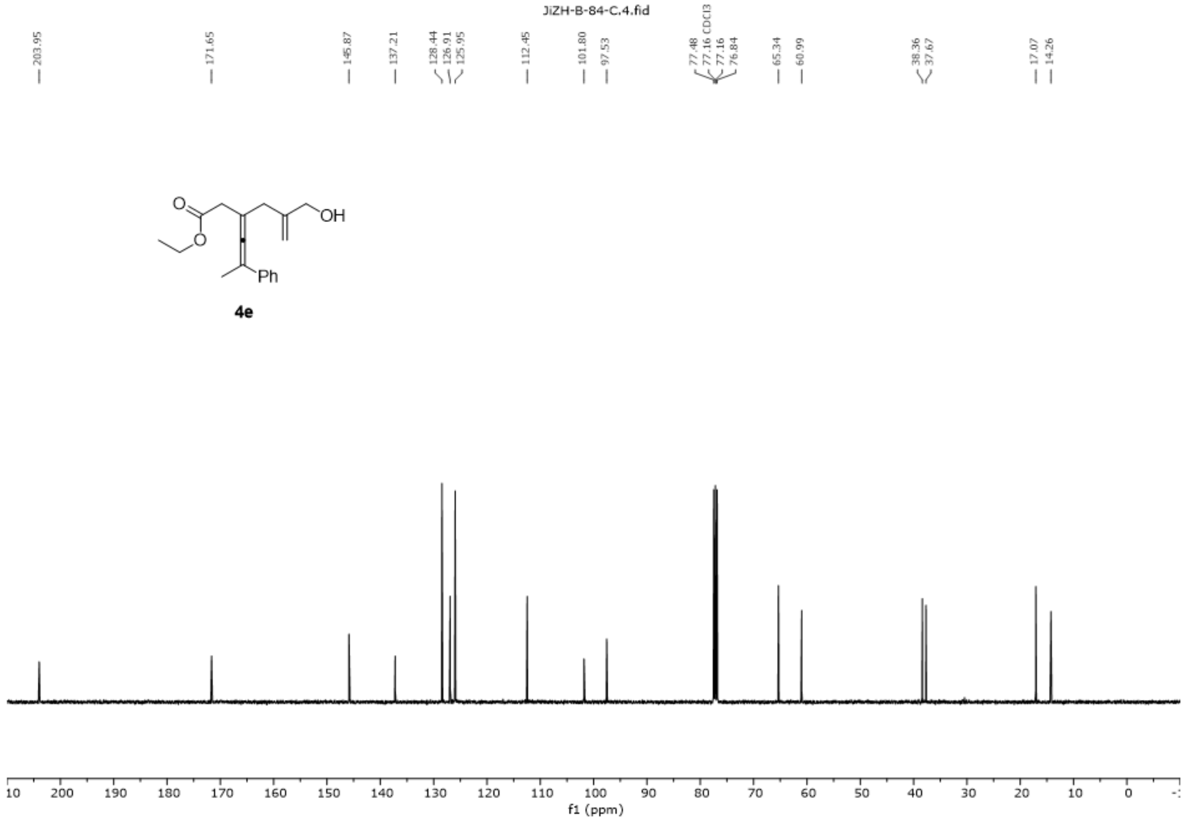
JiZH-B-84-C.4.fid
203.95
171.65
145.87
137.21
128.44 126.91 125.95
112.45
101.80
97.53
77.48 77.16 CDCl3 77.16 76.84
65.34
60.99
38.36 37.67
17.07
14.26
OH
O
O
Ph
4e
f1 (ppm)

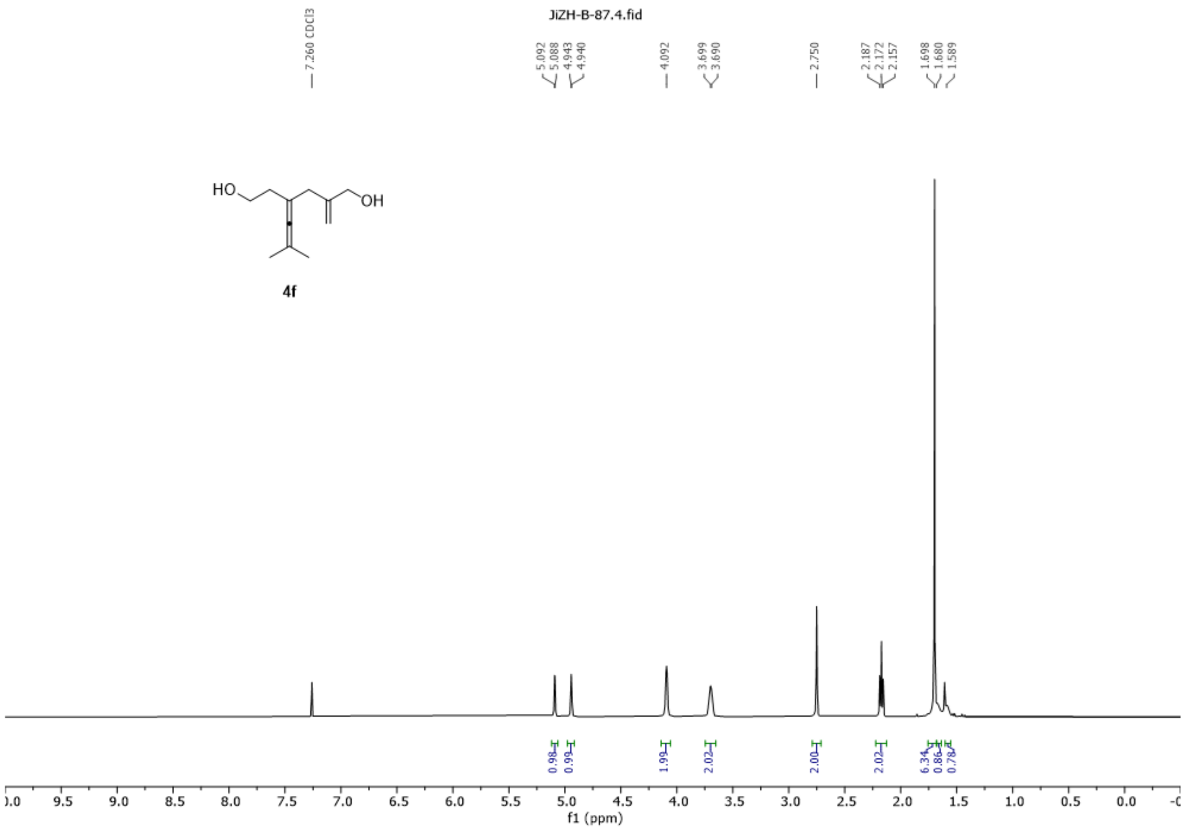
JiZH-B-87.4.fid
7.260 CDCl3
5.092
5.088
4.943
4.940
4.092
3.699
3.690
2.750
2.187
2.172
2.157
1.698
1.680
1.589
HO
OH
4f
0.98
0.99
1.99
2.02
2.00
2.02
6.34
0.86
0.78
9.5 9.0 8.5 8.0 7.5 7.0 6.5 6.0 5.5 5.0 4.5 4.0 3.5 3.0 2.5 2.0 1.5 1.0 0.5 0.0
f1 (ppm)

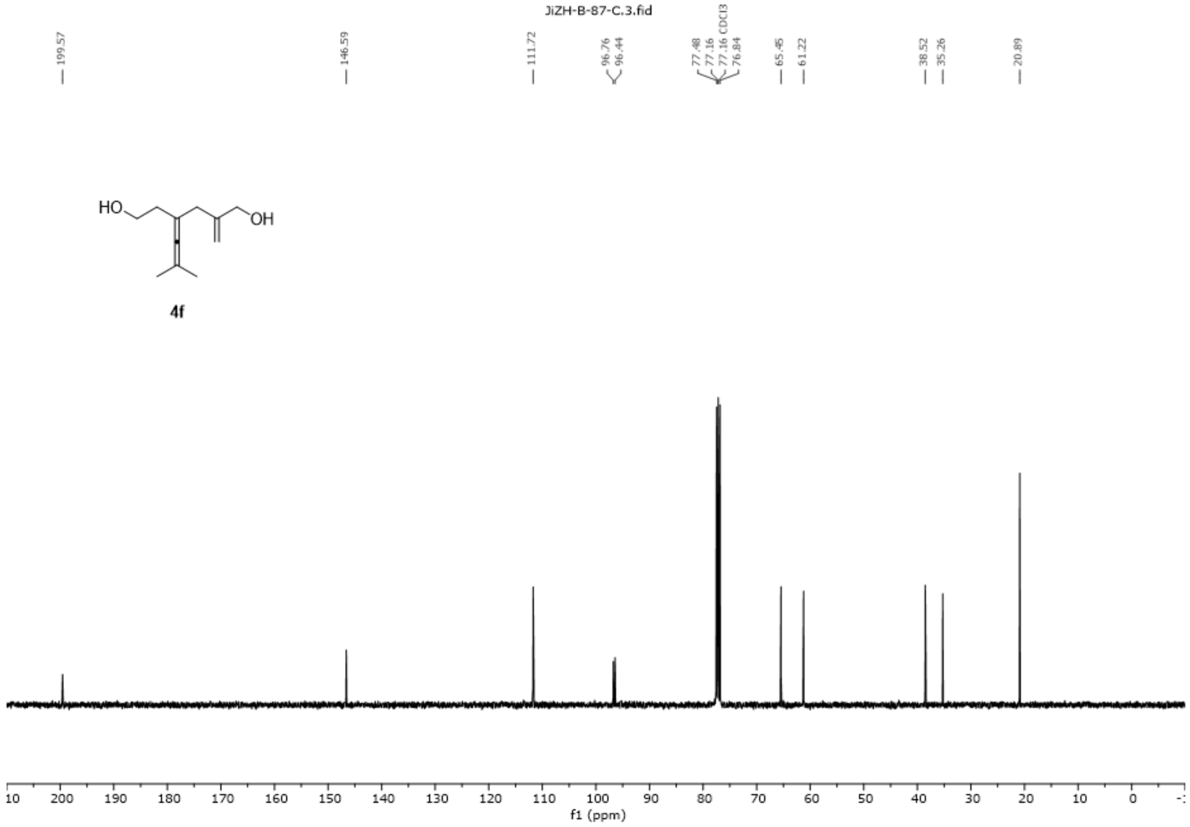
JiZH-B-87-C.3.fid
199.57
146.59
111.72
96.76
96.44
77.48
77.16
77.16 CDCl3
76.84
65.45
61.22
38.52
35.26
20.89
HO
OH
4f
200 190 180 170 160 150 140 130 120 110 100 90 80 70 60 50 40 30 20 10 0
f1 (ppm)

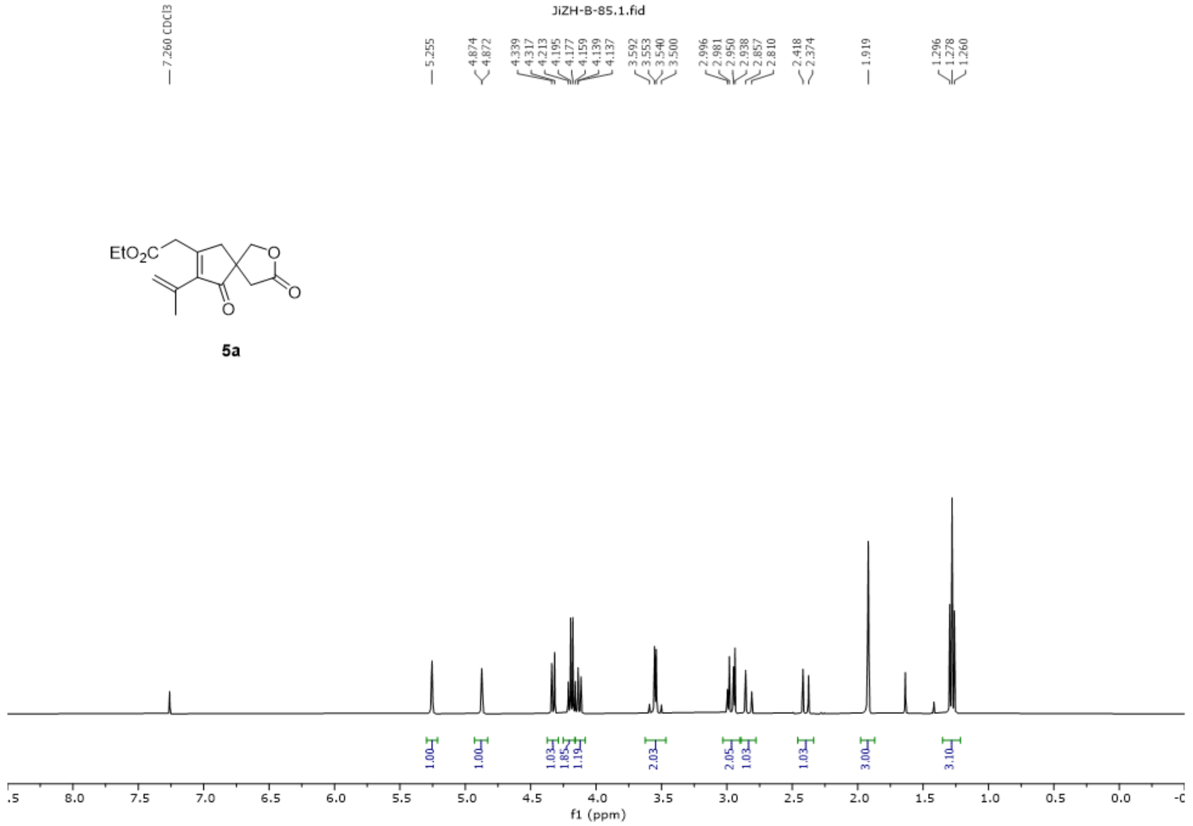
JiZH-B-85.1.fid
7.260 CDCl3
5.255
4.874
4.872
4.339
4.317
4.213
4.195
4.177
4.159
4.139
4.137
3.592
3.553
3.540
3.500
2.996
2.981
2.950
2.938
2.857
2.810
2.418
2.374
1.919
1.296
1.278
1.260
EtO2C
O
O
O
5a
1.00
1.00
1.03
1.85
1.19
2.03
2.05
1.03
1.03
3.00
3.10
f1 (ppm)

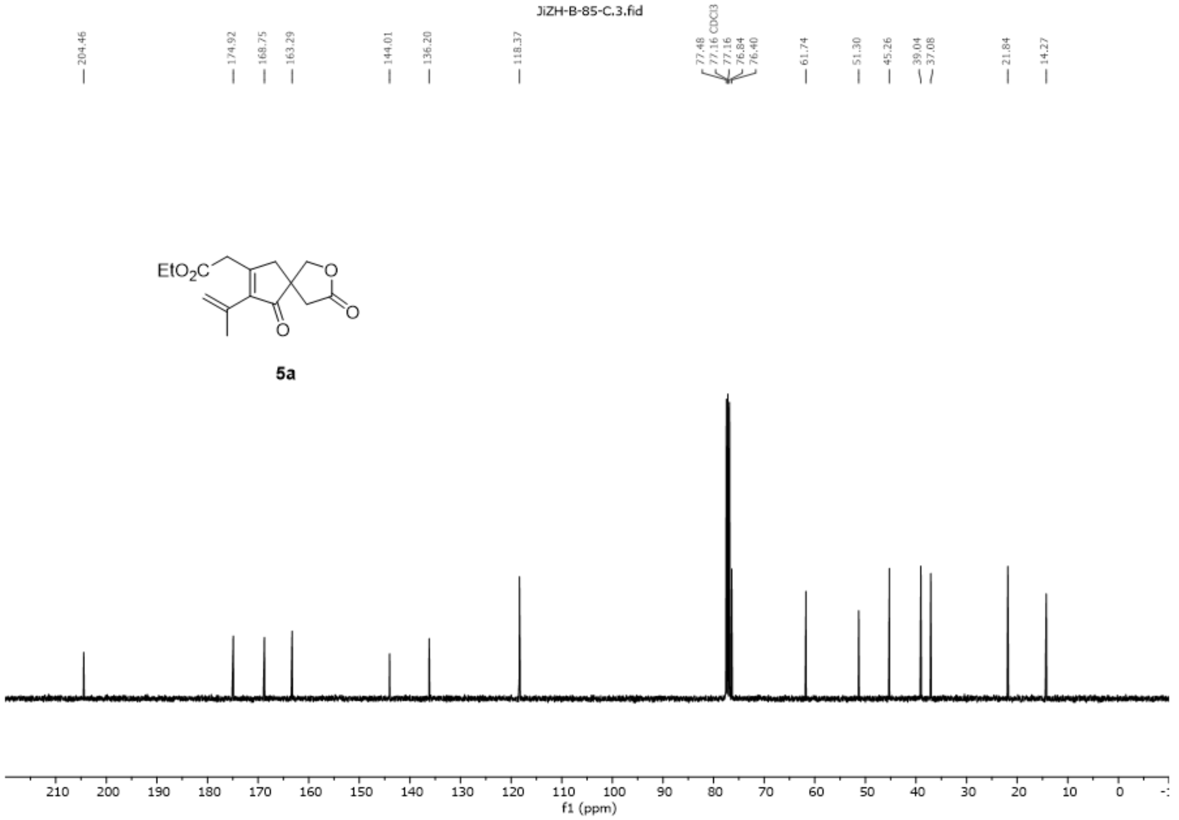
JiZH-B-85-C.3.fid
204.46
174.92
168.75
163.29
144.01
136.20
118.37
77.48
77.16 CDCl3
77.16
76.84
76.40
61.74
51.30
45.26
39.04
37.08
21.84
14.27
EtO2C
O
O
O
5a
f1 (ppm)

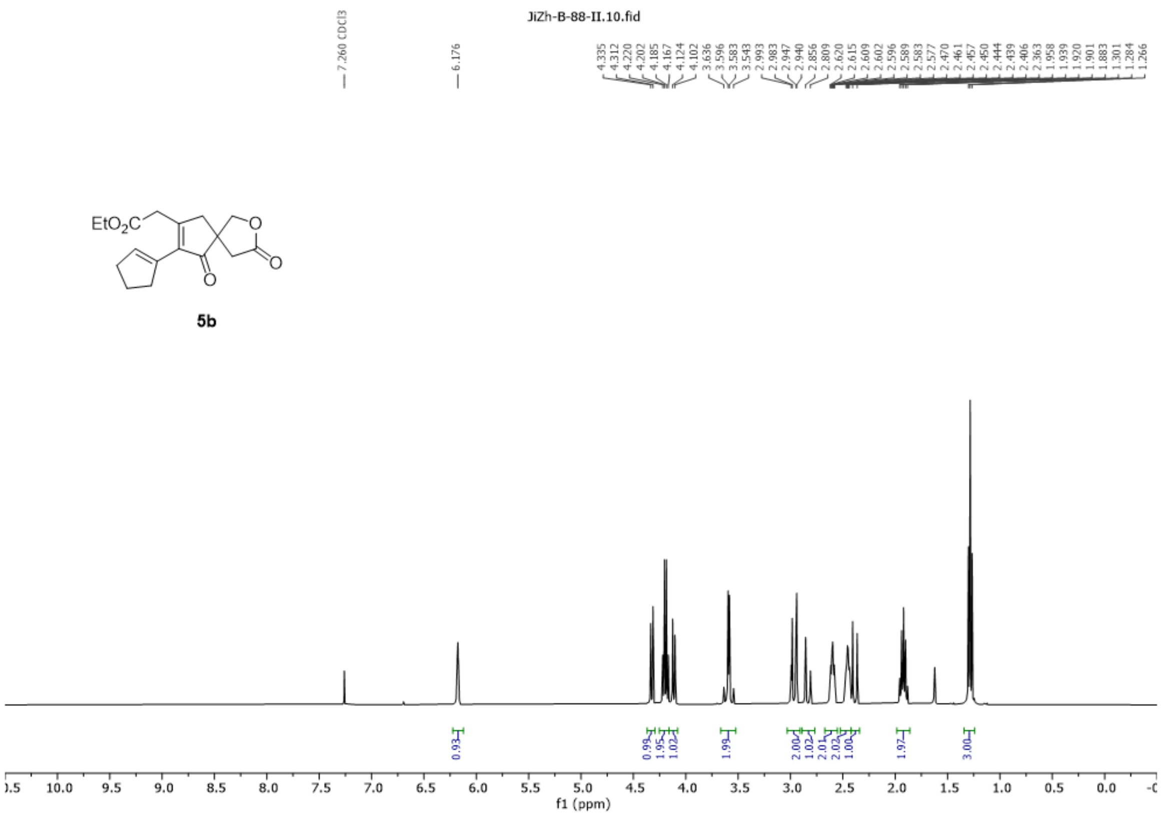
JiZh-B-88-II.10.fid
7.260 CDCl3
6.176
4.335 4.312 4.220 4.202 4.185 4.167 4.124 4.102 3.636 3.596 3.583 3.543 2.993 2.983 2.947 2.940 2.856 2.809 2.620 2.615 2.609 2.602 2.596 2.589 2.583 2.577 2.470 2.461 2.457 2.450 2.444 2.439 2.406 2.363 1.958 1.939 1.920 1.901 1.883 1.301 1.284 1.266
EtO2C
O
O
O
5b
0.93
0.99 1.95 1.02
1.99
2.00 1.02 2.01 2.02 1.00
1.97
3.00
f1 (ppm)

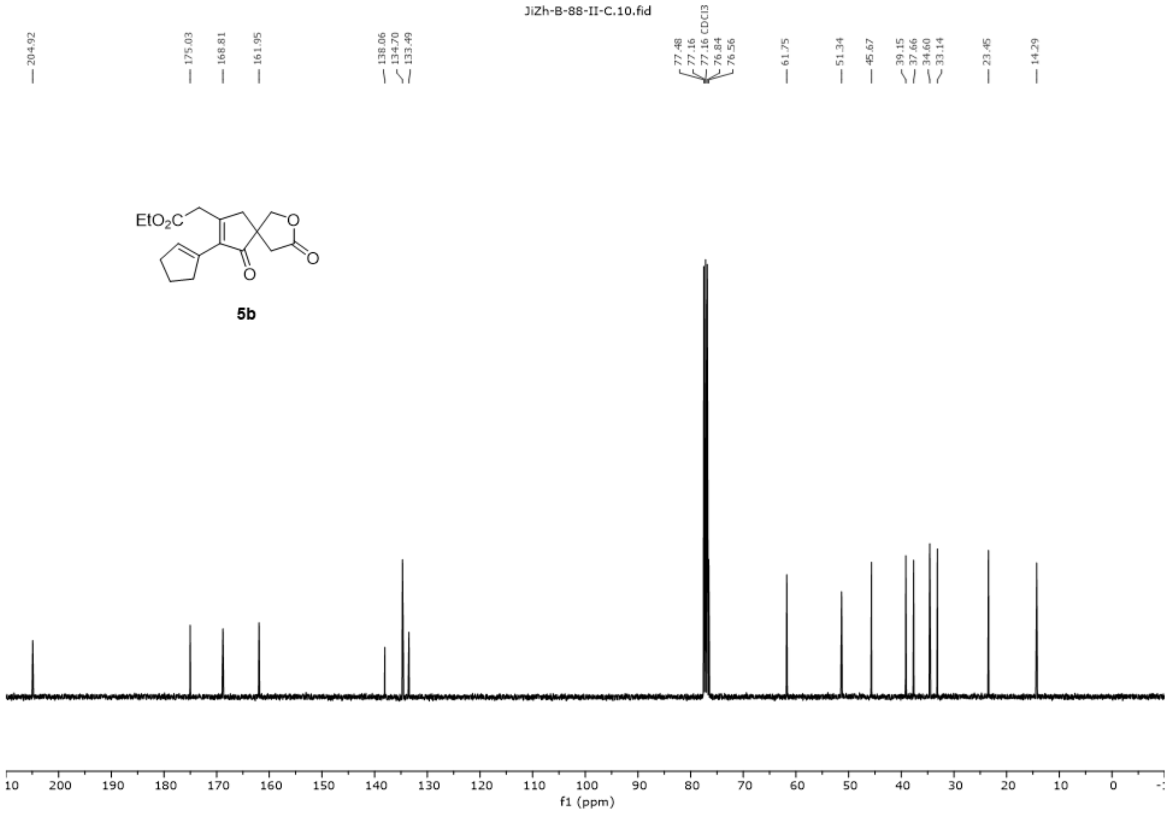
JiZh-B-88-II-C.10.fid
204.92
175.03
168.81
161.95
138.06 134.70 133.49
77.48 77.16 77.16 CDCl3 76.84 76.56
61.75
51.34
45.67
39.15 37.66 34.60 33.14
23.45
14.29
EtO2C
O
O
O
5b
f1 (ppm)

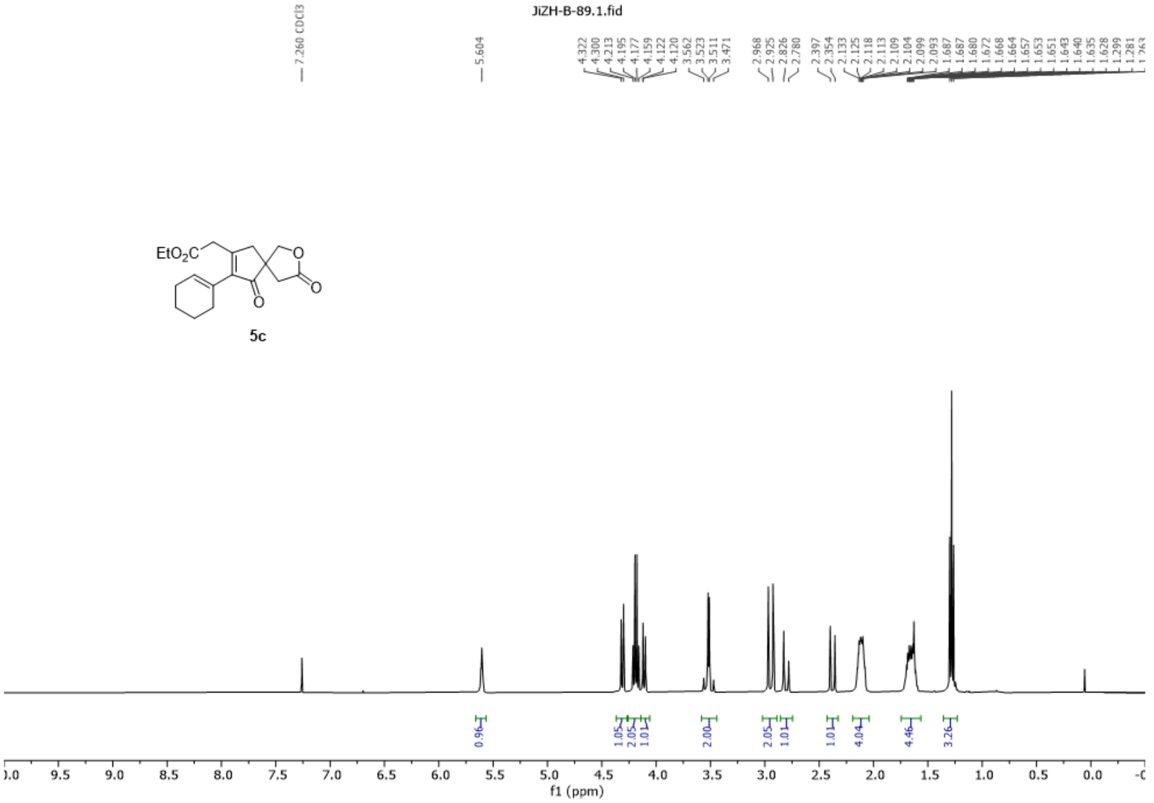
JiZH-B-89.1.fid
7.260 CDCl3
5.604
4.322
4.300
4.213
4.195
4.177
4.159
4.122
4.120
3.562
3.523
3.511
3.471
2.968
2.925
2.826
2.780
2.397
2.354
2.133
2.125
2.118
2.113
2.109
2.104
2.099
2.093
1.687
1.687
1.680
1.672
1.668
1.664
1.657
1.653
1.651
1.643
1.640
1.635
1.628
1.299
1.281
EtO_2C
O
O
O
5c
0.96
1.05
2.05
1.01
2.00
2.05
1.01
1.01
4.04
4.46
3.26
9.5
9.0
8.5
8.0
7.5
7.0
6.5
6.0
5.5
5.0
4.5
4.0
3.5
3.0
2.5
2.0
1.5
1.0
0.5
0.0
f1 (ppm)

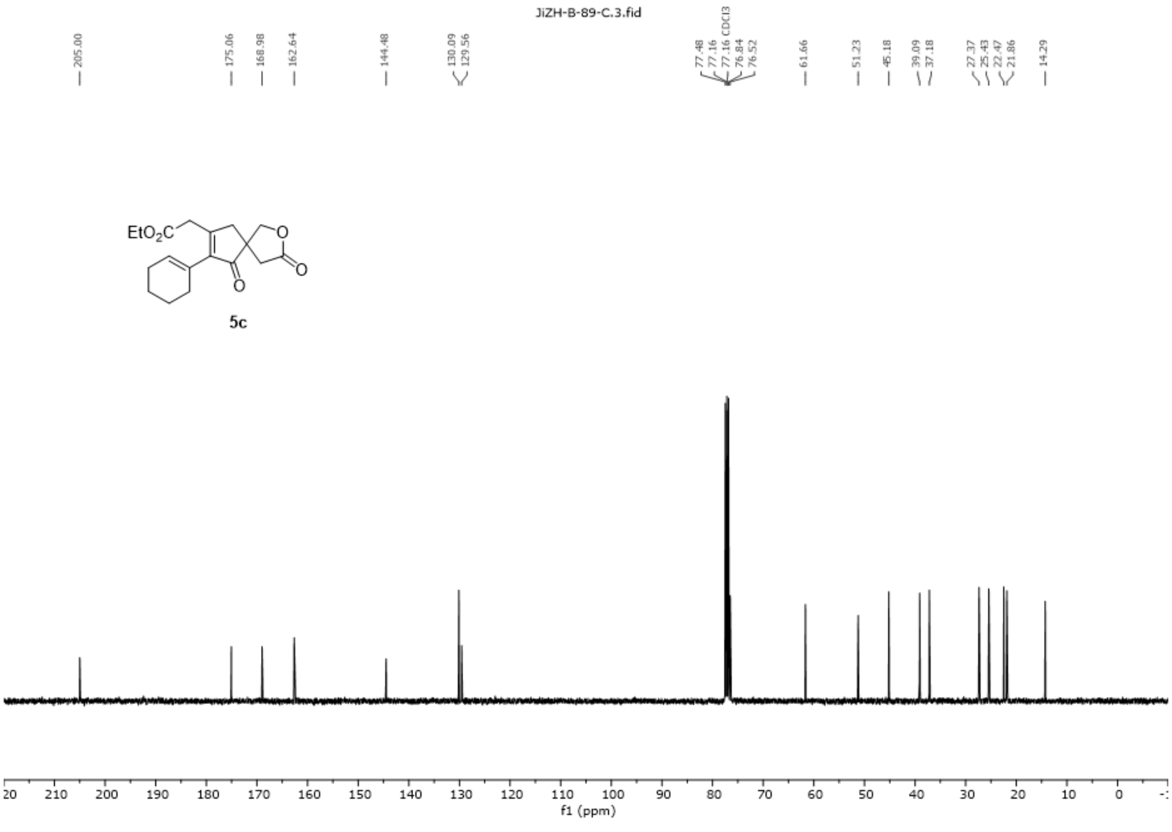
JiZH-B-89-C.3.fid
205.00
175.06
168.98
162.64
144.48
130.09
129.56
77.48
77.16
77.16 CDCl3
76.84
76.52
61.66
51.23
45.18
39.09
37.18
27.37
25.43
22.47
21.86
14.29
EtO_2C
O
O
O
5c
210
200
190
180
170
160
150
140
130
120
110
100
90
80
70
60
50
40
30
20
10
0
f1 (ppm)

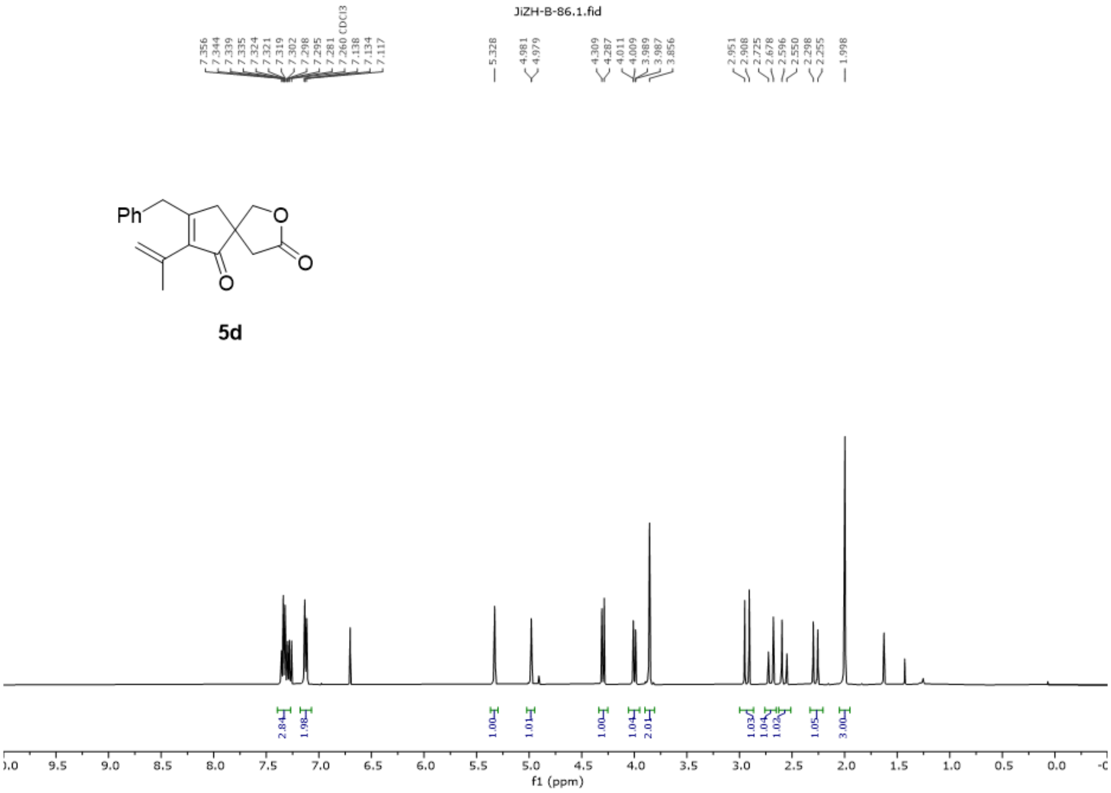
JiZH-B-86.1.fid
7.356
7.344
7.339
7.335
7.324
7.321
7.319
7.302
7.298
7.295
7.281
7.260 CDCl3
7.138
7.134
7.117
5.328
4.981
4.979
4.309
4.287
4.011
4.009
3.989
3.987
3.856
2.951
2.908
2.725
2.678
2.596
2.550
2.298
2.255
1.998
Ph
O
O
O
5d
2.84
1.98
1.00
1.01
1.00
1.04
2.01
1.03
1.04
1.02
1.05
3.00
f1 (ppm)

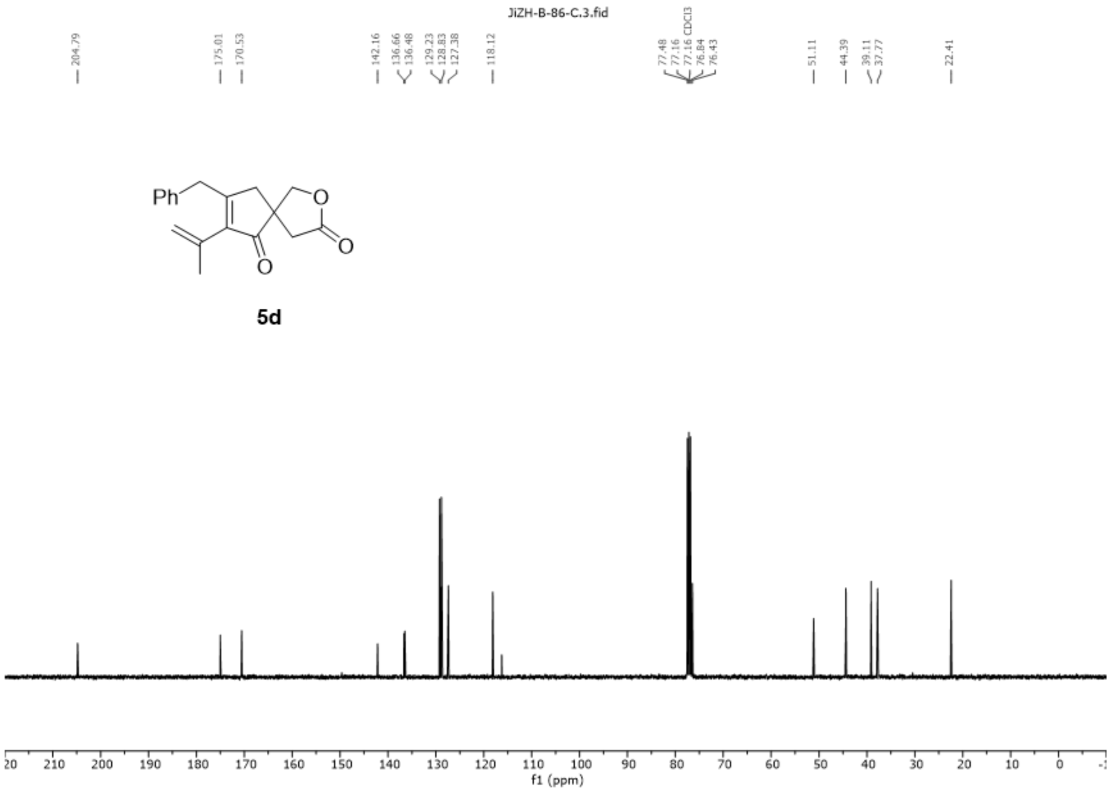
JiZH-B-86-C.3.fid
204.79
175.01
170.53
142.16
136.66
136.48
129.23
128.83
127.38
118.12
77.48
77.16 CDCl3
77.16
76.84
76.43
51.11
44.39
39.11
37.77
22.41
Ph
O
O
O
5d
f1 (ppm)

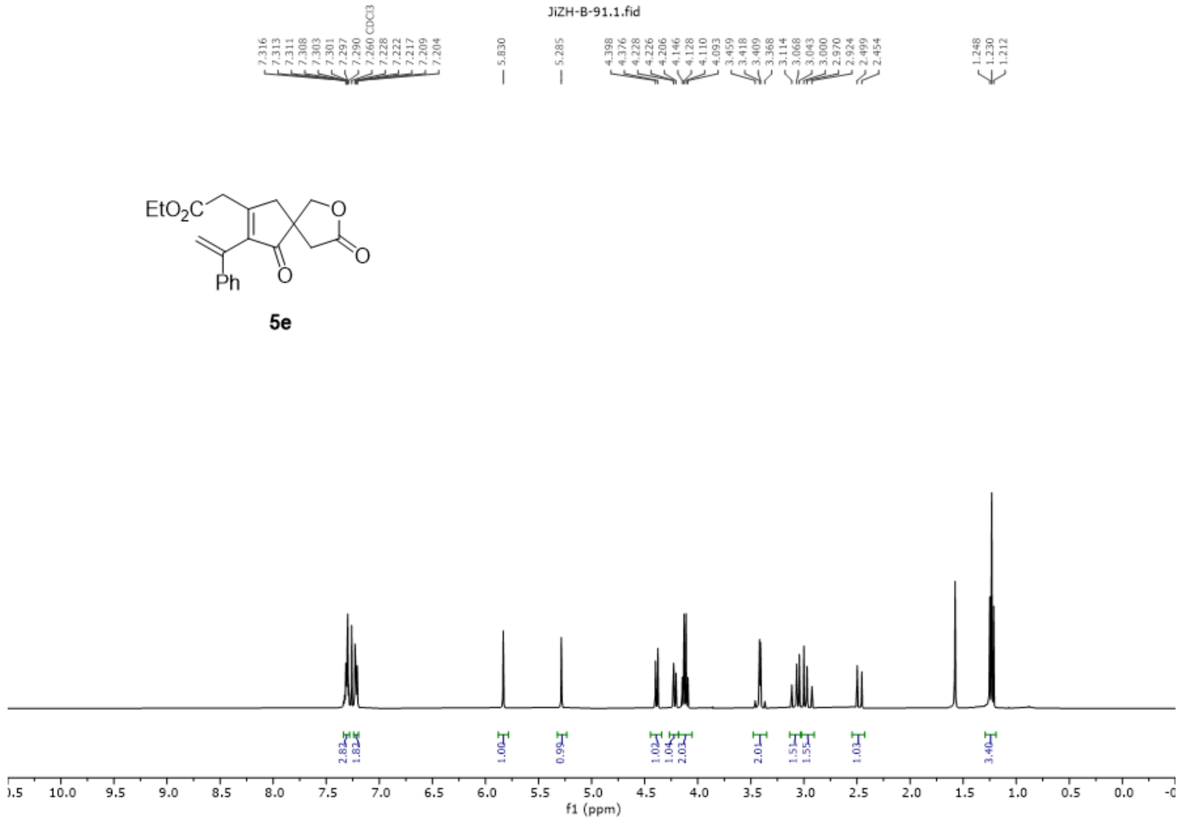
JiZH-B-91.1.fid
7.316 7.313 7.311 7.308 7.303 7.301 7.297 7.290 7.260 CDCl3 7.228 7.222 7.217 7.209 7.204
5.830
5.285
4.398 4.376 4.228 4.226 4.206 4.146 4.128 4.110 4.093 3.459 3.418 3.409 3.368 3.114 3.068 3.043 3.000 2.970 2.924 2.499 2.454
1.248 1.230 1.212
EtO2C
Ph
O
5e
2.82 1.82 1.00 0.99 1.02 1.04 2.03 2.01 1.51 1.55 1.03 3.40
f1 (ppm)

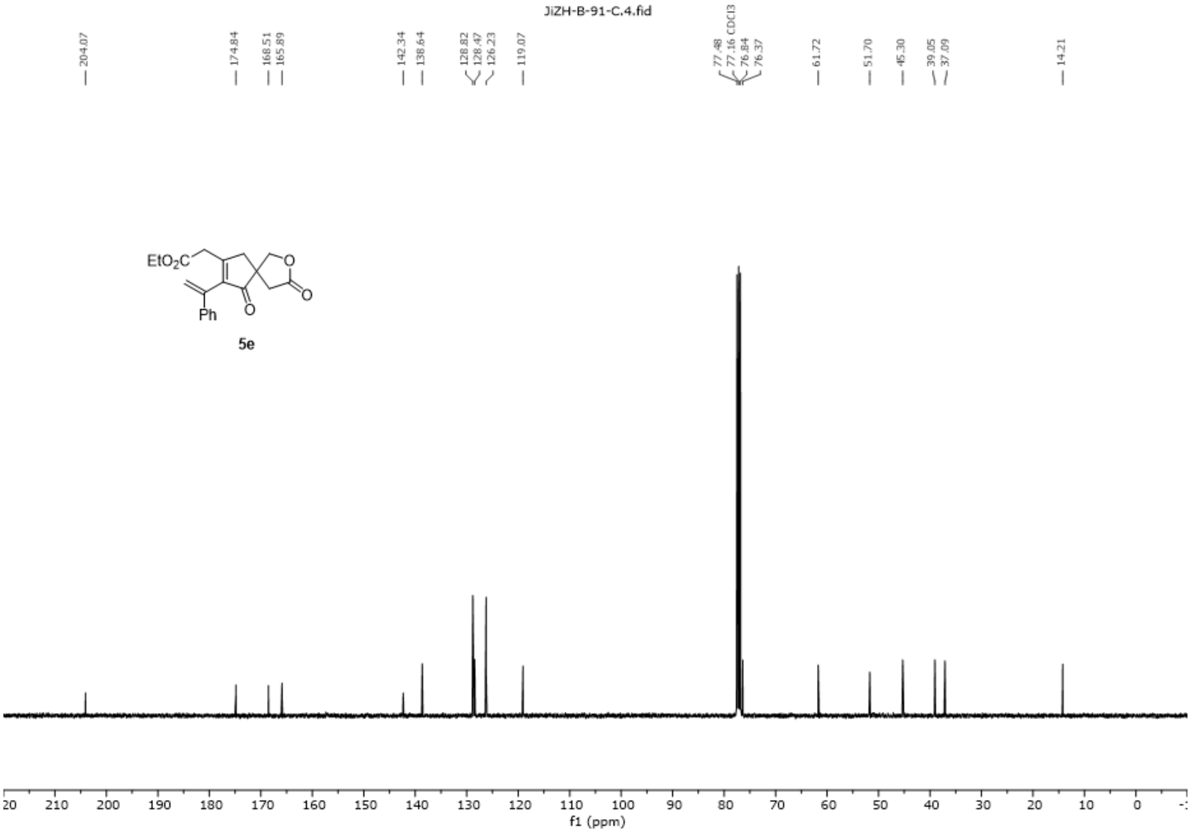
JiZH-B-91-C.4.fid
204.07
174.84 168.51 165.89
142.34 138.64
128.82 128.47 126.23
119.07
77.48 77.16 CDCl3 76.84 76.37
61.72
51.70
45.30
39.05 37.09
14.21
EtO2C
Ph
O
5e
f1 (ppm)

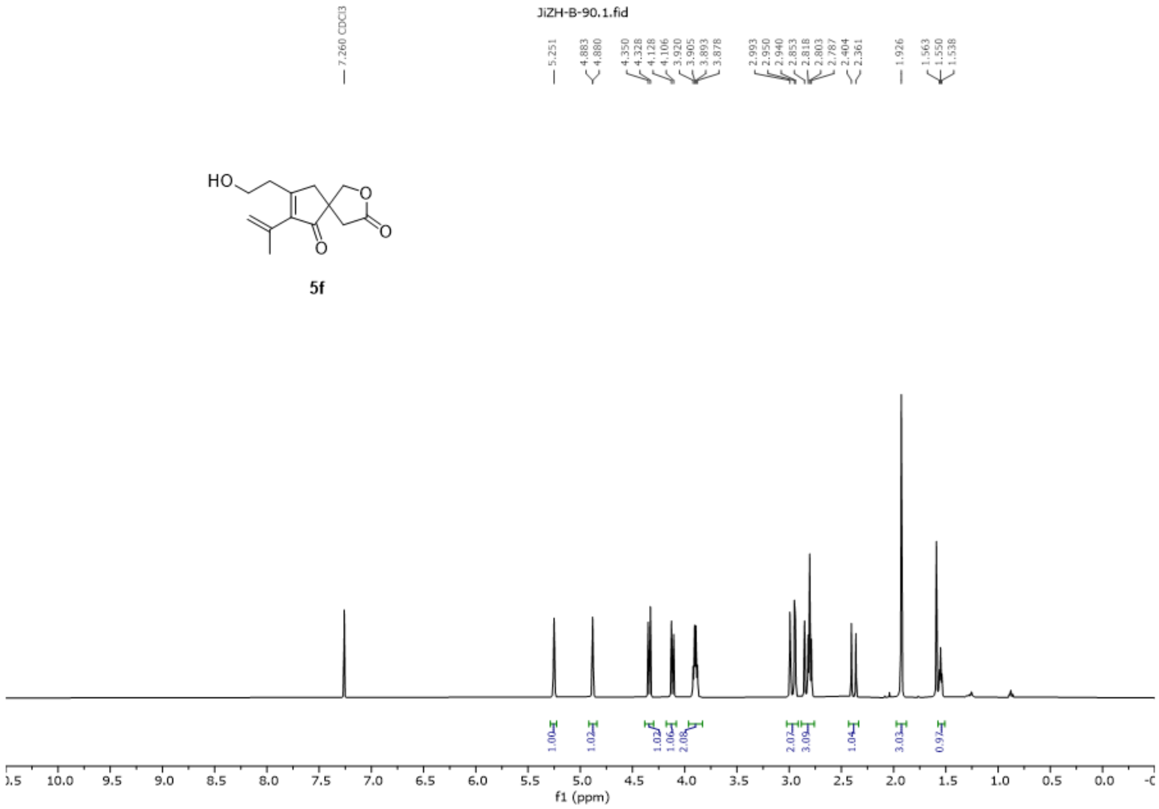
JiZH-B-90.1.fid
7.260 CDCl3
5.251
4.883
4.880
4.350
4.328
4.128
4.106
3.920
3.905
3.893
3.878
2.993
2.950
2.940
2.853
2.818
2.803
2.787
2.404
2.361
1.926
1.563
1.550
1.538
HO
O
O
O
5f
1.00
1.02
1.02
1.06
2.08
2.02
3.09
1.04
3.02
0.97
f1 (ppm)

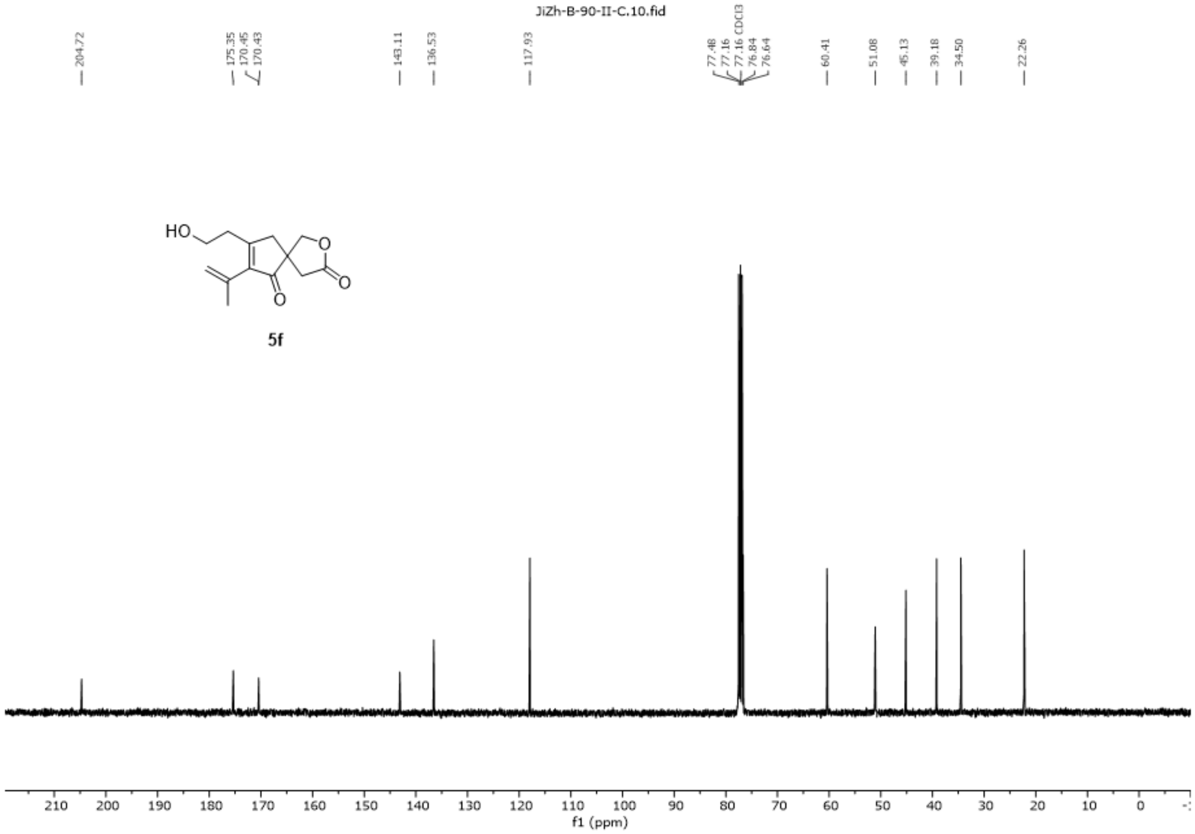
JiZh-B-90-II-C.10.fid
204.72
175.35
170.45
170.43
143.11
136.53
117.93
77.48
77.16
77.16 CDCl3
76.84
76.64
60.41
51.08
45.13
39.18
34.50
22.26
HO
O
O
O
5f
f1 (ppm)
